# Diversity-oriented synthesis of spirothiazolidinediones and their biological evaluation

**DOI:** 10.3762/bjoc.15.269

**Published:** 2019-11-18

**Authors:** Sambasivarao Kotha, Gaddamedi Sreevani, Lilya U Dzhemileva, Milyausha M Yunusbaeva, Usein M Dzhemilev, Vladimir A D’yakonov

**Affiliations:** 1 Department of Chemistry, Indian Institute of Technology Bombay, Powai, Mumbai 400 076, India; 2 Laboratory of Catalytic Synthesis, Institute of Petrochemistry and Catalysis, Russian Academy of Sciences, Prospect Octyabrya, 141, 450075, Ufa, Russian Federation,; 3 Department of Immunology and Human Reproductive Health Bashkir State Medical University, Lenin Street, 3, 450003, Ufa, Russian Federation

**Keywords:** apoptosis, biologically active, [2 + 2 + 2] cycloaddition, flow cytometry, spiro thiazolidinedione

## Abstract

We report a new synthetic approach to assemble spirothiazolidinediones via a [2 + 2 + 2] cyclotrimerization reaction and the derivatives were further functionalized through DA chemistry and click reaction. Using flow cytometry, it was shown for the first time that the new benzyl alcohol derivatives of thiazolidine-2,4-dione generated here are efficient apoptosis inducers in the HeLa, Hek293, U937, Jurkat, and K562 cell lines.

## Introduction

**Scheme 1:**
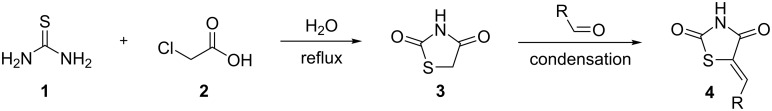
Conventional method of synthesis of thiazolidine-2,4-dione derivatives.

Heterocyclic compounds play a vital role in the metabolism of all living cells. Thus, most of the biologically active and pharmaceutically important compounds consist of heterocycles [1–4]. Especially, thiazolidinediones (TZDs) are important heterocyclic compounds. In 1954, Visentini reported the first ever pharmacological evaluation of a thiazolidinedione derivative, i.e., its anti-TB activity [3]. The promising activity shown by the compounds containing a thiazolidinedione nucleus cover numerous categories such as antihyperglycaemics [5], aldose reductase inhibitors (ARI) [6–7], anti-inflammatory [8–9], anti-arthritics [10], anticancer [11–13] and antimicrobial [14–18], etc., has made them an indispensable anchor for the development of new therapeutic agents [19–24]. Thiazolidinedione derivatives, such as rosiglitazone, pioglitazone and troglitazone, etc., are members of the glitazone family of drugs, used for the treatment of diabetes as potent agonists of the γ-peroxisome proliferator activated receptor (PPARγ) [23–24]. The discovery of biological activities of TZDs and the development of medicinal and pharmaceutical chemistry has led to an enhanced interest in the design of new synthetic methods for the preparation of various TZDs. The most conventional method of synthesis of thiazolidinedione derivatives is refluxing chloroacetic acid (**2**) with thiourea (**1**), followed by a Knoevenagel condensation with an aldehyde (Scheme 1) [25].

## Results and Discussion

Limited reports are available dealing with the synthesis of spiro derivatives of thiazolidine-2,4-diones [26–28]. In view of the importance of thiazolidine-2,4-dione derivatives, we conceived a new synthetic strategy to diverse spirocyclic thiazolidinediones based on a [2 + 2 + 2] cyclotrimerization [29–51] as a key step.

**Scheme 2:**
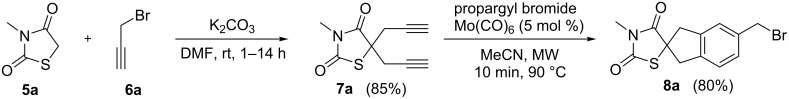
[2 + 2 + 2] Cyclotrimerization of *N*-methylthiazolidinedione.

In this regard, the required diyne precursor of thiazolidinedione was prepared from *N*-methylthiazolidine-2,4-dione (**5a**) and propargyl bromide (**6a**) in the presence of K_2_CO_3_ in DMF to afford the dipropargylated intermediate thiazolidinedione **7a** in 85% yield. Diyne **7a** was then reacted with propargyl bromide (**6a**) in the presence of Mo(CO)_6_ in acetonitrile at 90 °C under microwave irradiation (MWI) conditions to give the co-trimerized spiro derivative **8a** (Scheme 2).

**Scheme 3:**
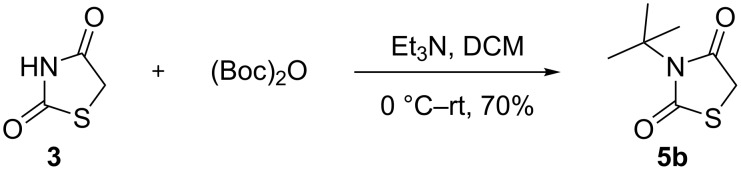
Unexpected product **5b** obtained in the attempted NH-protection of thiazolidinedione with (Boc)_2_O.

**Figure 1:**
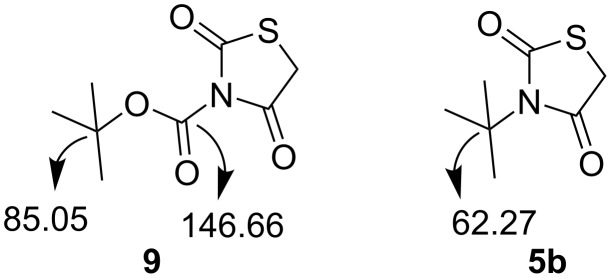
Comparison of ^13^C NMR values of **9** and **5b**.

**Scheme 4:**
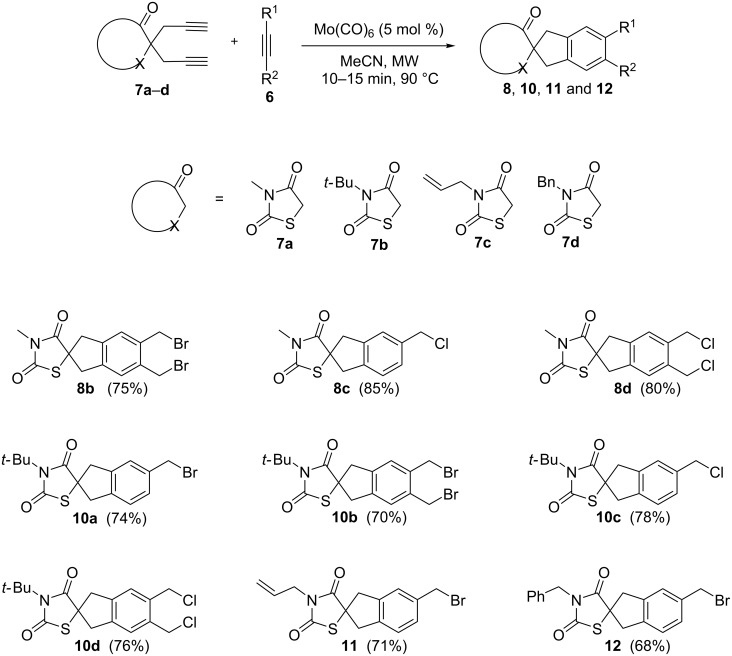
[2 + 2 + 2] Cyclotrimerization of dipropargylthiazolidinediones with propargyl halides.

The free NH moiety of thiazolidinedione **3** was alkylated using alkyl or aryl halides in the presence of Et_3_N using DCM as solvent. To our surprise, during an attempted protection with (Boc)_2_O, we obtained *N*-*tert*-butylthiazolidinedione **5b** rather than the expected *N*-Boc-protected thiazolidinedione **9** as reported by Paladhi et al. [52] (Scheme 3). The formation of compound **5b** was confirmed on the basis of spectral data. We observed only two peaks in the carbonyl region of the ^13^C NMR spectrum and no third carbonyl peak (Figure 1). Finally, mass spectral (HRMS) data confirmed the molecular formula. Next, we prepared the diyne precursors and examined the [2 + 2 + 2] cyclotrimerization strategy with different propargyl halides **6** to obtain spirothiazolidinedione derivatives (Scheme 4).

**Scheme 5:**
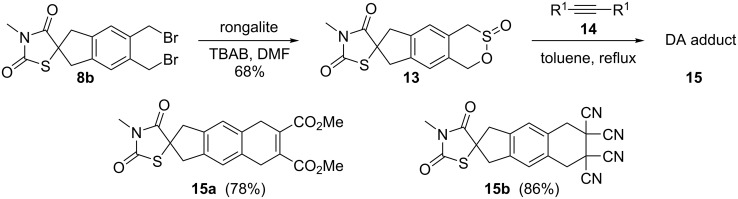
Formation of sultine **13** from compound **8b** followed by DA reaction.

Further, the dibromo *N*-methyl derivative of thiazolidinedione **8b** was converted to sultine **13** using rongalite and tetra-*n*-butylammonium bromide (TBAB) in DMF. Then, sultine **13** was treated with different dienophiles **14** in a DA fashion and linearly fused spirocyclic derivatives **15** were isolated in good yields (Scheme 5).

Since TZDs are pharmaceutically important targets, we intended to prepare spirocyclic derivatives of thiazolidinedione. In this context, *N*-carboxy ester and *N*-propargyl derivatives of thiazolidinedione were prepared, which on further modification, can generate new derivatives suitable for the multiple interaction sites. Further, the antitumour activity of these compounds were studied.

Therefore, we synthesized the required diyne derivatives of thiazolidinedione for [2 + 2 + 2] cycloaddition, in the presence of propargyl bromide and K_2_CO_3_ in DMF. The dipropargyl derivatives **7e** and **7f** were then treated with different propargyl alcohols **16** in the presence of Wilkinson’s catalyst/Ti(OiPr)_4_ under ethanol reflux conditions [53] to obtain the corresponding [2 + 2 + 2] cyclotrimerized alcohols **17–19** and **21** in good yields. During the [2 + 2 + 2] cycloaddition of compound **7e** with 3-butyn-1-ol (**16b**) we were able to isolate the self trimerized derivative **20** as a minor product (Scheme 7).

**Scheme 6:**
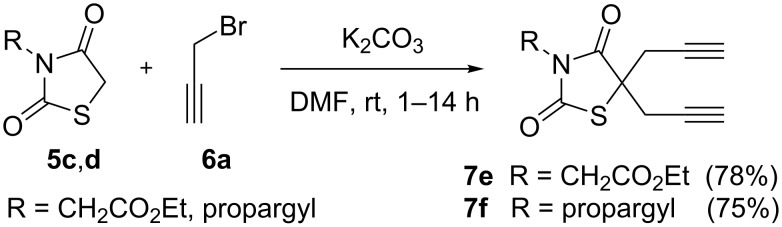
Dipropargylation of 2,4-thiazolidinedione derivatives.

**Scheme 7:**
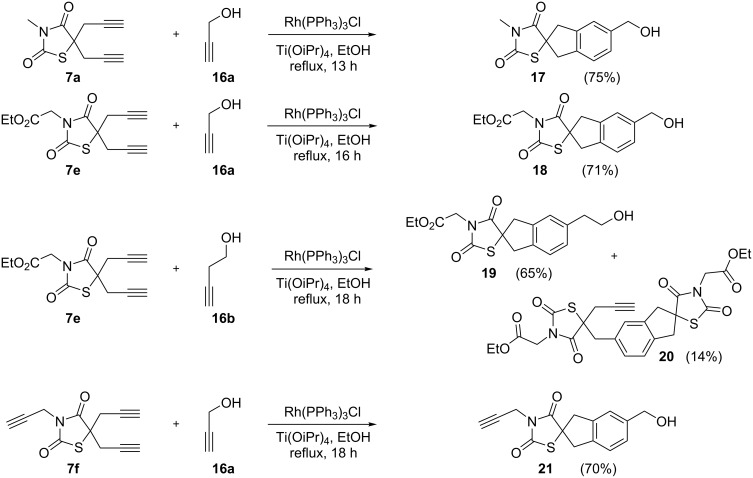
[2 + 2 + 2] Cycloaddition in the presence of Wilkinson’s catalyst.

**Scheme 8:**
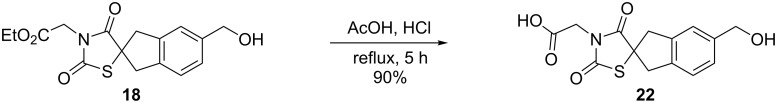
*N*-Ester derivative **18** hydrolysis to *N*-acid derivative **22**.

The ester derivative of thiazolidinedione **18** was hydrolyzed under AcOH, HCl reflux conditions to obtain *N*-acid derivative **22** in 90% yield (Scheme 8) [54].

**Scheme 9:**
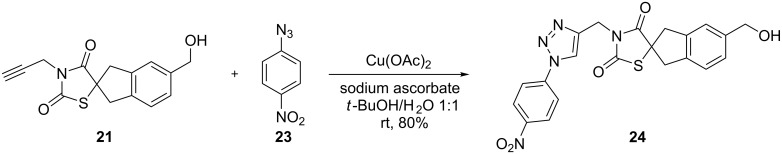
Synthesis of triazolo derivative **24** via click reaction.

Fused 1,2,3-triazoles represent an important class of nitrogen-containing biologically active compounds which exhibit various biological properties, such as antiviral, antibacterial and anticancer, etc. [55–58]. Recently, the use of 1,2,3-triazole derivatives as drug candidates has been increased for clinical therapy of various diseases. Hence, we also modified the *N*-propargyl alcohol derivative **21** to 1,2,3-triazolo alcohol derivative **24** by click chemistry using 4-nitrophenyl azide (**23**) and the corresponding triazolo alcohol derivative **24** was obtained as yellow solid in 80% yield (Scheme 9) [58].

All alcohol derivatives of thiazolidinedione were used to study the cytotoxic activity in comparison with camptothecin and etoposide on Jurkat, K562, HEK293, HELA, A549 and U937 cell lines. Compounds **20** and **17** showed the highest activity (IC_50_ = 0.29 and 0.36 nM, respectively) for leukemic monocytic lymphoma cells (U937), and **22** was active against T-cell leukemia (Jurkat) with an IC_50_ value of 0.34 nM. Further, these three compounds (**22**, **20** and **17**) were used to study apoptosis and the cell cycle in cells of the corresponding cell cultures. The highest percentage of apoptosis was observed when compound **22** was added to the culture of Jurkat cells at a concentration of 0.75 nM and was 68.65%. Under similar conditions, compound **20** showed about 95% in K562 cells of late apoptosis and compound **17** about 30.28% for cell line U937. The results of flow cytometry showed that in all three cell lines, Jurkat, K562 and U937, a hypodiploidal DNA peak appears 24 hours after the action of compounds **22**, **20** and **17**, which shows the inability to stop the fission cycle at the checkpoints, and ultimately, leads to cell death.

## Conclusion

In summary, we have synthesized spirothiazolidinedione derivatives via a [2 + 2 + 2] cyclotrimerization strategy with propargyl halides. We have also shown the synthesis of linearly fused spirocyclic alcohol derivatives of thiazolidinedione using Wilkinson’s catalyst and these compounds were tested for their anticancer activity. We have shown for the first time that the new benzyl alcohol derivatives of thiazolidinedione generated here are efficient apoptosis inducers in the HeLa, Hek293, U937, Jurkat, and K562 cell lines.

## Experimental

**General**: All commercially available products were used as received without further purification and moisture-sensitive reagents were transferred by using syringe–septum techniques. All the solvents used as reaction media were dried over oven-dried molecular sieves (4 Å). Column chromatography was performed with silica gel (100–200 mesh) using mixtures of petroleum ether and EtOAc as eluent. ^1^H NMR and ^13^C NMR spectral data were recorded with 400 MHz and 100 MHz or 500 MHz and 125 MHz spectrometers using tetramethylsilane (TMS) as an internal standard and chloroform-*d* as a solvent. High resolution mass spectrometry (HRMS) was performed using a Bruker (Maxis Impact) or a Micromass Q-ToF spectrometer. The melting points recorded are uncorrected. The microwave used here was a Discover^®^ SP by CEM Corporation and all the microwave reactions were performed under the standard method, where time and temperature can be monitored manually.

**Cell culture:** Cells (Jurkat, K562, U937) were purchased from the Russian Cell Culture Collection (Institute of Cytology of the Russian Academy of Sciences) and cultured according to standard mammalian tissue culture protocols and sterile technique. Human cancer cell lines HEK293 and HeLa were obtained from the HPA Culture Collections (UK). All cell lines used in the study were tested and shown to be free of mycoplasma and viral contamination.

Cell culture was performed in a similar manner as described in [59]. HEK293 and HeLa cell lines were cultured as monolayers and maintained in Dulbecco’s modified Eagle’s medium (DMEM, Gibco BRL) supplemented with 10% foetal bovine serum and 1% penicillin streptomycin solution at 37 °C in a humidified incubator under a 5% CO_2_ atmosphere.

Cells were maintained in RPMI 1640 (Jurkat, K562, U937) (Gibco) supplemented with 4 mM glutamine, 10% FBS (Sigma) and 100 units/mL penicillin streptomycin (Sigma). All types of cells were grown in an atmosphere of 5% CO_2_ at 37 °C. The cells were subcultured at 2–3 days intervals. Adherent cells (HEK293, HeLa) were suspended using trypsin/EDTA and counted after they have reached 80% confluency. Cells were then seeded in 24 well plates at 5 × 10^4^ cells per well and incubated overnight. Jurkat, K562, U937 cells were subcultured at 2 day intervals with a seeding density of 1 × 10^5^ cells per 24 well plates in RPMI with 10% FBS.

**MTT Assay:** The MTT assay is a colorimetric assay for evaluation of cell metabolic activity. The NADPH-dependent cellular oxidoreductases present in the living cell can reflect, under certain conditions, the cell viability. These enzymes can reduce the tetrazolium dye, 3-(4,5-dimethylthiazol-2-yl)-2,5-diphenyl-2*H*-tetrazolium bromide (МТТ) or yellow tetrazole, to give insoluble formazan, which develops purple color particularly in living cells. Thus, the color gradient can serve to determine the degree of cytostatic activity (shift from proliferation to dormancy) of drug candidates and toxic compounds. In vitro cytotoxicity was assessed using a standard MTT colorimetric assay in a similar manner as described in [60–61]. HEK293 cells were plated in 96-well microassay culture plates (1 × 10^4^ cells per well) and grown overnight at 37 °C in a 5% CO_2_ incubator. The cells were then incubated with the test compounds at different concentrations for an additional 24 h. Control wells were prepared by the addition of dimethyl sulfoxide (DMSO, 1%). At the end of this incubation, 20 μL of MTT (5 mg/mL in PBS) was added to each well. After incubation for 4 h, the formazan crystals were dissolved in 150 μL of DMSO, and the absorbance was determined at 595 nm using a microplate spectrophotometer. The IC_50_ value was determined from plots of percent viability against the dose of the compound added.

**Cytotoxicity assay:** Viability (live/dead) assessment was performed by staining cells with 7-AAD (7-aminoactinomycin D, Biolegend) in a similar manner as described in [62]. In brief, after treatment cells were harvested, washed 1–2 times with phosphate-buffered saline (PBS) and centrifuged at 400*g* for 5 minutes. Cell pellets were resuspended in 200 µL of flow cytometry staining buffer (PBS without Ca^2+^and Mg^2+^, 2.5% FBS) and stained with 5 µL of 7-AAD staining solution for 15 minutes at room temperature in the dark. Samples were acquired on a NovoCyteTM 2000 Flow Cytometry System (ACEA) equipped with a 488 nm argon laser. Detection of 7-AAD emission was collected through a 675/30 nm filter in FL4 channel.

**Annexin V-FITC/PI assay:** The apoptosis-inducing activity of the compounds was analyzed quantitatively using an Alexa Fluor^®^ 488 Annexin V/Dead Cell Apoptosis Kit in a similar way as described in [60]. Phosphatidylserine externalization on the outer surface of the plasma membrane is an exact and reliable indication of cell apoptosis or necrosis. The difference between these two forms of cell death is that in the early stages of apoptosis, the cell membrane remains relatively undamaged, whereas upon necrosis, the cell membrane loses integrity. In the living, normally functioning cells, phosphatidylserine is present on the cell surface membrane in minor quantity; hence, its interaction with the V Alexa Fluor^®^ 488 annexin is insignificant. Furthermore, undamaged cell membrane is impermeable for propidium iodide. At the early stage of apoptosis, phosphatidylserine molecules appear on the cell surface but the membrane is still impermeable for DNA-binding dyes (such as propidium iodide). The membrane integrity is lost at later stages of the cell death. Thus, while detecting the apoptosis, one can distinguish four types of cells: living cells (annexin V−/PI−), early apoptosis (annexin V+/PI−), late apoptosis (annexin V+/PI+), and necrosis (annexin V−/PI+).

Apoptosis was quantified by the detection of phosphatidylserine surface exposure on apoptotic cells using an Alexa Fluor^®^ 488 Annexin V/Dead Cell Apoptosis Kit. HEK293 cells were incubated with or without the testing compound for 24 h. The adherent and floating cells were collected and washed twice with cold PBS. Then, the cells were resuspended in 90 μL of annexin V binding buffer (10 mM HEPES, 140 mM NaCl, 2.5 mM CaCl_2_; pH 7.4). Annexin V (5 μL) and 1 μL of propidium iodide were added to the reaction mixture and incubated for 15 min at room temperature in the dark. After the addition of 300 μL of binding buffer, the stained cells were analyzed with a NovoCyteTM 2000 FlowCytometry System (ACEA).

## Supporting Information

File 1 Experimental details, characterization data and copies of spectra.
