# Supplementary material for: Diversity-oriented synthesis of spirothiazolidinediones and their biological evaluation
Source: Beilstein J Org Chem. 2019 Nov 18;15:2774–81. doi: 10.3762/bjoc.15.269 (PMC6880819; doi:10.3762/bjoc.15.269)
Supplement: File 1 — Experimental details, characterization data and copies of spectra. [file Beilstein_J_Org_Chem-15-2774-s001.pdf]

BEILSTEIN JOURNAL OF ORGANIC CHEMISTRY

Supporting Information

for

Diversity-oriented synthesis of spirothiazolidinediones and their biological evaluation

Sambasivarao Kotha, Gaddamedi Sreevani, Lilya U. Dzhemileva, Milyausha M. Yunusbaeva, Usein M. Dzhemilev and Vladimir A. D'yakonov

Beilstein J. Org. Chem. **2019,** *15,* 2774–2781. doi:10.3762/bjoc.15.269

Experimental details, characterization data and copies of spectra

License and Terms: This is a supporting information file under the terms of the Creative Commons Attribution License (http://creativecommons.org/licenses/by/4.0). Please note that the reuse, redistribution and reproduction in particular requires that the authors and source are credited.
The license is subject to the *Beilstein Journal of Organic Chemistry* terms and conditions: (https://www.beilstein-journals.org/bjoc)

Table of contents

Experimental procedures and characterization of all compounds S2

NMR spectra S10

Cytotoxicity assay S37

Cell cycle analysis S40

References S42

Experimental section

General: All commercially available products were used as received without further purification and moisture-sensitive reagents were transferred by using syringe–septum techniques. All solvents used as reaction media were dried over oven-predried molecular sieves (4 Å). Column chromatography was performed with silica gel (100–200 mesh) using mixtures of petroleum ether and EtOAc as eluent. ^{1}H NMR and ^{13}C NMR spectral data were recorded with 400 MHz and 100 MHz or 500 MHz and 125 MHz spectrometers using tetramethylsilane (TMS) as an internal standard and chloroform-*d* as a solvent. The high resolution mass spectrometry (HRMS) was performed using Bruker (Maxis Impact) or Micromass Q-ToF spectrometer. The melting points recorded are uncorrected. The microwave used here was Discover® SP by CEM Corporation and all the microwave reactions were performed under the standard method, where time and temperature can be monitored manually.

Methylation of thiazolidine-2,4-dione (3): To a suspension of K_2CO_3 (2.36 g, 17.1 mmol) in dry acetone (5 mL/1 mmol) 2,4-thiazolidinedione **3** (1 g, 8.54 mmol) was added and the mixture allowed to stir at 50–60 °C for 30 min. Later, Me_2SO_4 (1.6 mL, 17.1 mmol) was added at the same temperature and the resulting mixture was stirred for another 12 h. After completion of the reaction (TLC), the reaction was stopped by adding water. The reaction mixture was extracted with EtOAc (3 × 20 mL). The combined organic phases were washed with brine, dried over anhydrous Na_2SO_4, filtered and concentrated. The crude residue was then purified by column chromatography on silica gel with EtOAc/petroleum ether 5:95 afforded *N*-methylthiazolidine-2,4-dione (**5a**, 0.95 g, 85%) as a colorless oily liquid. The NMR spectra matched with the literature reports [1].

General procedure for the preparation of *N*-protected thiazolidine-2,4-diones: $(Boc)_2O$, propargyl bromide, allyl bromide or benzyl bromide (2 equiv) was added to a solution of thiazolidinedione (1 equiv) and Et_3N (2 equiv) in CH_2Cl_2 (0.7 M) at 0 °C. Then the reaction mixture was brought to rt and stirred for 7–10 h at rt. The reaction mixture was quenched with water and extracted with CH_2Cl_2 (3 × 20 mL). The combined organic phases were washed with brine, dried over anhydrous Na_2SO_4, filtered and concentrated. Then, the crude residue was purified by column chromatography on silica gel using the appropriate mixture of EtOAc/petroleum ether 5:95 to 10:90 to provide the corresponding *N*-protected thiazolidine-2,4-dione. The NMR spectra of the compounds matched with the literature reports [1,2]. However, in the case of Boc protection we observed *tert*-butyl protection.

***N*-(*tert*-Butyl)thiazolidine-2,4-dione (5b):** Colorless oil; 517.5 mg (70%); ^{1}H NMR 500 MHz, $CDCl_3$) δ1.60 (s, 9H), 4.06 (s, 2H) ppm; ^{13}C NMR (125 MHz, $CDCl_3$) δ 28.6, 33.4, 62.3, 172.5, 173.0 ppm; IR (υ_{max}) 865, 899, 1038, 1141, 1193, 1258, 1316, 1370, 1460, 1480, 1685, 1755, 2938, 2979 cm^{-1}; HRMS (ESI, Q-ToF) *m/z* calcd for $C_7H_{11}KNO_2S$ $[M+K]^+$ 212.0142, found 212.0144.

General procedure for the dipropargylation of *N*-protected thiazolidine-2,4-diones: The dipropargylation of the *N*-protected thiazolidine-2,4-diones was performed in a similar manner as described in [3]. To a suspension of the *N*-protected thiazolidine-2,4-dione (1 equiv) and K_2CO_3 (5 equiv) in dry DMF (0.5 M) was added propargyl bromide (1.5 or 3 equiv). The resulting mixture was stirred at rt for 1 h to 18 h. After completion of the reaction (TLC), the reaction was quenched by adding water and the mixture was extracted with EtOAc (3 × 20 mL). The combined organic phases were washed with brine, dried over anhydrous Na_2SO_4, filtered and concentrated. Then, the crude residue was purified by column chromatography on silica gel with EtOAc/hexane 1:99 to 10:95 to give the desired compound.

***N*-Methyl-5,5-di(2-propynyl)thiazolidine-2,4-dione (7a):** Colorless high density liquid; 671.56 mg (85%); ^{1}H NMR (500 MHz, $CDCl_3$) δ 2.13 (t, J = 2.60 Hz, 2H), 2.87-3.0 (dq, J_1 = 2.60 Hz, J_2 = 14.35 Hz, 4H), 3.12 (s, 3H) ppm; ^{13}C NMR (125 MHz, $CDCl_3$) δ 28.1, 29.0, 60.0, 73.1, 77.5, 170.4, 175.1 ppm; IR (υ_{max}) 981, 1017, 1045, 1281, 1374, 1428, 1682, 1751, 2918, 2951, 3289 cm^{-1}; HRMS (ESI, Q-ToF) *m/z* calcd for $C_{10}H_{10}NO_2S$ $[M+H]^+$ 208.0427, found 208.0427.

***N*-(*tert*-Butyl)-5,5-di(2-propynyl)thiazolidine-2,4-dione (7b):** Colorless high density liquid; 561.33 mg (78%); ^{1}H NMR (500 MHz, $CDCl_3$) δ 1.61 (s, 9H), 2.12 (t, J = 2.60 Hz, 2H), 2.81-2.90 (dq, J_1 = 2.60 Hz, J_2 = 16.85 Hz, 4H) ppm; ^{13}C NMR (125 MHz, $CDCl_3$) δ 28.6, 29.4, 58.2, 62.8, 72.8, 77.8, 170.8, 176.4 ppm; IR (υ_{max}) 863, 912, 1026, 1039, 1150, 1191, 1259, 1307, 1331, 1370, 1424, 1461, 1683, 1751, 2855, 2928, 2977, 3294 cm^{-1}; HRMS (ESI, Q-ToF) *m/z* calcd for $C_{13}H_{15}NNaO_2S$ $[M+Na]^+$ 272.0716, found 272.0716.

***N*-Allyl-5,5-di(2-propynyl)thiazolidine-2,4-dione (7c):** Colorless high density liquid; 112.79 mg (76%); ^{1}H NMR (500 MHz, $CDCl_3$) δ 2.13 (t, J = 2.6 Hz, 2H), 2.88-2.98 (dq, J_1 = 2.6 Hz, J_2 = 16.95 Hz, 4H), 4.21-4.23 (td, J_1 = 1.25 Hz, J_2 = 5.85 Hz, 2H), 5.20-5.22 (dd, J_1 = 1.05 Hz, J_2 = 10.25 Hz, 1H), 5.29-5.32 (dd, J_1 = 1.1 Hz, J_2 = 17.10 Hz, 1H), 5.74-5.81 (m, 1H) ppm; ^{13}C NMR (125 MHz, $CDCl_3$) δ 29.1, 44.2, 60.6, 73.2, 77.4, 119.2, 130.0, 169.9, 174.6 ppm; HRMS (ESI, Q-ToF) *m/z* calcd for $C_{12}H_{12}NO_2S$ $[M+H]^+$ 234.0583, found 234.0588.

***N*-Benzyl-5,5-di(2-propynyl)thiazolidine-2,4-dione (7d):** Colorless high density liquid; 95.7 mg, (70%); ^{1}H NMR (500 MHz, $CDCl_3$) δ 1.93 (t, J = 2.50 Hz, 2H), 2.85-2.94 (dq, J_1 = 2.50 Hz, J_2 = 16.95 Hz, 4H), 4.77 (s, 2H), 7.27-7.40 (m, 5H) ppm; ^{13}C NMR (125 MHz, $CDCl_3$) δ 29.0, 45.6, 60.5, 73.1, 77.2, 128.3, 128.6, 129.1, 135.1, 170.1, 174.8 ppm; DEPT135 NMR (125 MHz, $CDCl_3$) δ 29.0, 45.6, 73.1, 77.2, 128.3, 128.6, 129.1 ppm; HRMS (ESI, Q-ToF) *m/z* calcd for $C_{16}H_{13}NaNO_2S$ $[M+Na]^+$ 306.0559, found 306.0551.

Compound 7e: Colorless high density liquid; 536 mg (78%); ^{1}H NMR (500 MHz, $CDCl_3$) δ 1.28 (t, J = 7.15 Hz, 3H), 2.17 (t, J = 2.55 Hz, 2H), 2.93-3.04 (dq, J_1 = 2.55 Hz, J_2 = 17.00 Hz, 4H), 4.19-4.23 (q, J = 7.15 Hz, 2H), 4.34 (s, 2H) ppm; ^{13}C NMR (125 MHz, $CDCl_3$) δ 14.2, 28.8, 42.2, 61.1, 62.2, 73.3, 77.2, 166.1, 169.6, 174.3 ppm; IR (υ_{max}) 667, 862, 976, 1004, 1028, 1115, 1165, 1216, 1257, 1327, 1354, 1387, 1409, 1693, 1747, 2125, 2912, 2987, 3284 cm^{-1}; HRMS (ESI, Q-ToF) *m/z* calcd for $C_{13}H_{13}NNaO_4S$ $[M+Na]^+$ 302.0457, found 302.0457.

3,5,5-Tri(2-propynyl)thiazolidine-2,4-dione (7f): Colorless high density liquid; 558.9 mg (75%); ^{1}H NMR (400 MHz, $CDCl_3$) δ 2.14 (t, J = 2.54 Hz, 2H), 2.23 (t, J = 2.44 Hz, 1H), 2.89-2.01 (dq, J_1 = 2.52 Hz, J_2 = 9.76 Hz, 4H), 4.37 (d, J = 2.48 Hz, 2H) ppm; HRMS (ESI, Q-ToF) *m/z* calcd for $C_{12}H_{10}NO_2S$ $[M+H]^+$ 232.0427, found 232.0427.

General procedure for the [2 + 2 + 2] cycloaddition under MWI conditions: The [2 + 2 + 2] cycloaddition was performed in a similar manner as described in [3]. In brief, to a solution of diyne (1 equiv) and propargyl halide (2 equiv) in dry acetonitrile (5 mL), $Mo(CO)_6$ (5 mol %) was added and the reaction mixture was stirred under MWI conditions for 10–15 min. After completion of the reaction (TLC), the reaction mixture was concentrated at reduced pressure and the crude product was purified by silica gel column chromatography using EtOAc/petroleum ether 5:95 to 30:70 to give the [2 + 2 + 2] cyclotrimerized compounds.

Compound 8a: Colorless high density liquid; 63.0 mg (80%); ^{1}H NMR (500 MHz, $CDCl_3$) δ 3.19 (s, 3H), 3.39 (d, J = 16.35 Hz, 2H), 3.87-3.91 (dd, J_1 = 3.85 Hz, J_2 = 12.55 Hz, 2H), 4.49 (s, 2H), 7.21 (d, J = 7.70 Hz, 1H), 7.27 (d, J = 7.95 Hz, 2H) ppm; ^{13}C NMR (125 MHz, $CDCl_3$) δ 28.4, 33.5, 47.3, 47.3, 64.4, 124.9, 125.3, 128.8, 137.7, 139.7, 140.0, 171.2, 177.0 ppm; IR (υ_{max}) 950, 1024, 1212, 1249, 1275, 1284, 1324, 1367, 1424, 1682, 1747, 2847, 2915, 2951, 3017 cm^{-1}; HRMS (ESI, Q-ToF) *m/z* calcd for $C_{13}H_{12}BrNNaO_2S$ $[M+Na]^+$ 347.9668, found 347.9663 and other isotopic peak is 349.9645.

Compound 8b: Colorless crystalline solid; 75.9 mg (75%); MP 202-204°C; ^{1}H NMR (400 MHz, $CDCl_3$) δ 3.19 (s, 3H), 3.40 (d, J = 16.45 Hz, 2H), 3.89 (d, J = 16.33 Hz, 2H), 4.64 (s, 4H), 7.26

(s, 2H) ppm; ^{13}C NMR (100 MHz, $CDCl_3$) δ 28.4, 30.2, 47.2, 64.0, 127.2, 136.5, 140.8, 170.9, 176.9 ppm; IR (υ_{max}) 614, 947, 1025, 1046, 1130, 1195, 1214, 1273, 1366, 1428, 1442, 1674, 1749, 2921 cm^{-1}; HRMS (ESI, Q-ToF) *m/z* calcd for $C_{14}H_{13}Br_2NNaO_2S$ $[M+Na]^+$ 439.8926, found 439.8922 and other isotopic peaks are 441.8899 and 443.8877.

Compound 8c: Colorless high density liquid; 57.69 mg (85%); ^{1}H NMR (500 MHz, $CDCl_3$) δ 3.16 (s, 3H), 3.36-3.39 (dd, J_1 = 2.05 Hz, J_2 = 14.40 Hz, 2H), 3.85-3.90 (dd, J_1 = 5.00 Hz, J_2 = 11.40 Hz, 2H), 4.55 (s, 2H), 7.23 (m, 3H) ppm; ^{13}C NMR (125 MHz, $CDCl_3$) δ 28.4, 46.2, 47.2, 47.3, 64.4, 124.9, 124.9, 128.4, 137.4, 139.6, 139.9, 171.2, 177.0 ppm; IR (υ_{max}) 719, 950, 1025, 1131, 1276, 1325, 1368, 1425, 1682, 1747, 2918, 2953 cm^{-1}; HRMS (ESI, Q-ToF) *m/z* calcd for $C_{13}H_{12}ClNNaO_2S$ $[M+Na]^+$ 304.0169, found 304.0164.

Compound 8d: Colorless crystalline solid; 63.77 mg (80%); MP 164-166 ^{o}C; ^{1}H NMR (400 MHz, $CDCl_3$) δ 3.19 (s, 3H), 3.41 (d, J = 8.26 Hz, 2H), 3.90 (d, J = 16.53 Hz, 2H), 4.73 (s, 4H), 7.30 (s, 2H) ppm; ^{13}C NMR (100 MHz, $CDCl_3$) δ 28.3, 43.3, 47.1, 64.1, 126.9, 136.1, 140.7, 170.9, 176.9 ppm; IR (υ_{max}) 677, 948, 1026, 1048, 1128, 1214, 1272, 1323, 1367, 1428, 1447, 1680, 1749, 2890, 2973 cm^{-1}; HRMS (ESI, Q-ToF) *m/z* calcd for $C_{14}H_{13}Cl_2NNaO_2S$ $[M+Na]^+$ 351.9936, found 351.9936.

Compound 10a: Sticky gel; 54.68 mg (74%); ^{1}H NMR (400 MHz, $CDCl_3$) δ 1.65 (s, 9H), 3.34 (d, J = 16.37 Hz, 2H), 3.82-3.87 (dd, J_1 = 3.0 Hz, J_2 = 16.45 Hz, 2H), 4.48 (s, 2H), 7.18 (m, 3H) ppm; ^{13}C NMR (100 MHz, $CDCl_3$) δ 28.8, 33.6, 47.3, 47.3, 61.9, 62.4, 124.9, 125.2, 128.7, 137.5, 140.0, 140.3, 171.3, 178.4 ppm; HRMS (ESI, Q-ToF) *m/z* calcd for $C_{16}H_{18}BrNNaO_2S$ $[M+Na]^+$ 390.0134, found 390.0131 and other isotopic peak is 392.0112.

Compound 10b: White solid; 64.8 mg (70%); ^{1}H NMR (500 MHz, $CDCl_3$) δ 1.65 (s, 9H), 3.34 (d, J = 16.55 Hz, 2H), 3.83 (d, J = 16.55 Hz, 2H), 4.64 (s, 4H), 7.23 (s, 2H) ppm; ^{13}C NMR (125 MHz, $CDCl_3$) δ 28.7, 30.2, 47.2, 61.6, 62.5, 127.1, 136.3, 141.1, 171.1, 178.2 ppm; HRMS (ESI, Q-ToF) *m/z* calcd for $C_{17}H_{19}Br_2NNaO_2S$ $[M+Na]^+$ 481.9395, found 481.9395 and other isotopic peaks are 483.9375 and 485.9354.

Compound 10c: Colorless liquid; 50.59 mg (78%); ^{1}H NMR (500 MHz, $CDCl_3$) δ 1.65 (s, 9H), 3.34 (d, J = 16.15 Hz, 2H), 3.83-3.87 (dd, J_1 = 5.45 Hz, J_2 = 16.30 Hz, 2H), 4.57 (s, 2H), 7.23 (m, 3H) ppm; ^{13}C NMR (125 MHz, $CDCl_3$) δ 28.7, 46.3, 47.2, 62.0, 62.3, 124.8, 124.8, 128.2, 137.2, 139.9, 140.2, 171.3, 178.3 ppm; HRMS (ESI, Q-ToF) *m/z* calcd for $C_{16}H_{18}ClNNaO_2S$ $[M+Na]^+$ 346.0639, found 346.0636.

Compound 10d: White solid; 56.77 mg (76%); ^{1}H NMR (500 MHz, $CDCl_3$) δ 1.65 (s, 9H), 3.35 (d, J = 16.50 Hz, 2H), 3.85 (d, J = 16.50 Hz, 2H), 4.73 (s, 4H), 7.26 (s, 2H) ppm; ^{13}C NMR (125 MHz, $CDCl_3$) δ 28.7, 43.4, 47.2, 61.7, 62.5, 126.8, 135.9, 141.0, 170.1, 178.2 ppm; HRMS (ESI, Q-ToF) m/z calcd for $C_{17}H_{19}Cl_2NNaO_2S$ $[M+Na]^+$ 394.0406, found 394.0405.

Compound 11: Sticky liquid; 37.54 mg (71%); ^{1}H NMR (500 MHz, $CDCl_3$) δ 3.40 (d, J = 16.35 Hz, 2H), 3.88-3.92 (dd, J_1 = 4.15 Hz, J_2 = 16.45 Hz, 2H), 4.28 (d, J = 5.85 Hz, 2H), 4.49 (s, 2H), 5.24-5.29 (m, 2H), 5.81-5.89 (m, 1H), 7.20-7.28 (m, 3H) ppm; ^{13}C NMR (125 MHz, $CDCl_3$) δ 33.5, 44.2, 47.3, 64.2, 119.1,124.9, 125.3, 128.8, 130.3, 137.7, 139.7, 140.0, 170.6, 176.5 ppm; HRMS (ESI, Q-ToF) m/z calcd for $C_{15}H_{14}BrNNaO_2S$ $[M+Na]^+$ 373.9821, found 373.9822.

Compound 12: Sticky liquid; 38.64 mg (68%); ^{1}H NMR (500 MHz, $CDCl_3$) δ 3.40 (d, J = 16.35 Hz, 2H), 3.85-3.89 (dd, J_1 = 4.15 Hz, J_2 = 16.45 Hz, 2H), 4.49 (s, 2H), 4.83 (s, 2H), 7.20 (d, 1H), 7.26-7.28 (m, 2H), 7.32-7.37 (m, 3H), 7.40-7.42 (m, 2H) ppm; ^{13}C NMR (125 MHz, $CDCl_3$) δ 33.5, 45.6, 47.1, 47.2, 64.1, 124.9, 125.2, 128.4, 128.7, 128.8, 128.9, 135.3, 137.6, 139.6, 140.0, 170.8, 176.7 ppm; DEPT135 NMR (125 MHz, $CDCl_3$) δ 33.5, 45.6, 47.1, 47.2, 124.9, 125.2, 128.4, 128.75, 128.82, 128.9 ppm; HRMS (ESI, Q-ToF) m/z calcd for $C_{19}H_{16}BrNNaO_2S$ $[M+Na]^+$ 423.9977, found 423.9976.

Synthesis of sultine derivative of 2,4-thiazolidinedione 13: The sultine derivative of 2,4-thiazolidinedione was prepared in a similar way as described in [4]. To a solution of dibromo compound **8b** (150 mg, 0.36 mmol) and TBAB (1 equiv) in DMF (5mL), was added rongalite (422.44 mg, 3.58 mmol) at 0 °C and the reaction mixture was stirred at 0 °C for 3 h and at rt for another 3 h. At the conclusion of the reaction (TLC monitoring), the aqueous layer was extracted with EtOAc and the organic layer was washed with water (4–5 times) to remove excess of DMF. The solvent was removed under reduced pressure and the crude product was purified by silica gel column chromatography using EtOAc/petroleum ether 15:85 to give a white solid (78.63 mg, 68% yield). ^{1}H NMR (500 MHz, $CDCl_3$) δ 3.18 (d, J = Hz,3H), 3.40 (m, 2H), 3.56 (d, J = 16.25 Hz, 1H), 3.90 (m, 2H), 4.36-4.40 (dd, 1H), 4.92-4.95 (dd, J = 16.30 1H), 5.25-5.29 (dd, 1H), 7.13 (d,1H), 7.16 (d, 1H) ppm; ^{13}C NMR (100 MHz, $CDCl_3$) δ 28.4, 47.1, 47.1, 47.1,57.2, 57.5, 63.6, 63.8, 64.0, 64.4, 122.1, 122.2, 125.9, 126.0, 126.2, 133.6, 133.7, 139.3, 139.3, 140.0, 140.0, 170.9, 176.7, 176.9 ppm; HRMS (ESI, Q-ToF) m/z calcd for $C_{14}H_{14}NNaO_4S$ $[M+Na]^+$ 346.0178, found 346.0175.

General procedure for the DA reaction: The solution of compound (1 equiv) and dienophiles (1.5 equiv) in toluene (5 mL) was kept in the MW for 30 min at 130 °C. The solvent was

removed under reduced pressure and the crude product was purified by silica gel column chromatography (20–30% EtOAc/petroleum ether) to deliver the desired DA adducts.

Compound 15a: White solid; 33.89 mg (78%); ^{1}H NMR (500 MHz, $CDCl_3$) δ 3.18 (s, 3H), 3.34 (d, J = 16.25 Hz, 2H), 3.69 (s, 4H), 3.82 (s, 6H), 3.85 (d, J = 16.30 Hz, 2 H), 7.04 (s, 2H) ppm; ^{13}C NMR (100 MHz, $CDCl_3$) δ 28.4, 31.7, 47.0, 52.6, 64.7, 124.0, 131.4, 133.5, 138.1, 168.2, 171.3, 177.0 ppm; IR (υ_{max}) 786, 857, 920, 951, 1025, 1127, 1214, 1277, 1369, 1433, 1456, 1682, 1723, 2363, 2851, 2952, 3023 cm^{-1}; HRMS (ESI, Q-ToF) *m/z* calcd for $C_{20}H_{19}NNaO_6S$ $[M+Na]^+$ 424.0825, found 424.0827.

Compound 15b: White solid; 20.61 mg (86%); ^{1}H NMR (500 MHz, $CDCl_3$) δ 3.20(s, 3H), 3.42 (d, J = 16.60 Hz, 2H), 3.81 (s, 4H), 3.91 (d, J = 16.60 Hz, 2H), 7.12 (s, 2H) ppm; IR (υ_{max}) 669, 912, 1023, 1217, 1370, 1424, 1679, 1745, 2926, 3307 cm^{-1}; HRMS (ESI, Q-ToF) *m/z* calcd for $C_{20}H_{14}N_5O_2S$ $[M+H]^+$ 388.0863, found 388.0869.

General procedure for [2 + 2 + 2] cycloaddition using Wilkinson's catalyst: The [2 + 2 + 2] cycloaddition in the presence of Wilkinson's catalyst was performed in a similar way as described in [4]. In brief, the solution of compound **7a**, **7e**, or **7f** (1 equiv) and alcohol **16a** or **16b** (3 equiv) in dry ethanol (≈ 5 mL per mmol) was degassed with nitrogen for 15 min. Then, Wilkinson's catalyst (5 mol %) and $Ti(OiPr)_4$ (25 mol %) were added and the reaction mixture was refluxed for 13–18 h. At the conclusion of the reaction (TLC), the solvent was removed under reduced pressure and the crude product was purified by silica gel column chromatography (10–40% EtOAc/petroleum ether) to deliver the corresponding [2 + 2 + 2] cycloaddition products.

Compound 17: Colorless high density liquid; 190.58 mg (75%); ^{1}H NMR (500 MHz, $CDCl_3$) δ 2.34 (s, 1H); 3.16 (s, 3H), 3.35 (d, J = 16.30 Hz, 2H), 3.86 (d, J = 16.20 Hz, 2H), 4.63 (s, 2H), 7.19 (s, 2H), 7.23 (s, 1H) ppm; ^{13}C NMR (125 MHz, $CDCl_3$) δ 28.3, 47.1, 47.3, 64.6, 65.0, 123.2, 124.5, 126.6, 138.5, 139.5, 140.8, 171.3, 177.0 ppm; IR (υ_{max}) 816, 875, 950, 1026, 1132, 1227, 1242, 1280, 1325, 1369, 1426, 1493, 1682, 1747, 2918, 2953, 3018, 3500 cm^{-1}; HRMS (ESI, Q-ToF) *m/z* calcd for $C_{13}H_{13}NNaO_3S$ $[M+Na]^+$ 286.0508, found 286.0506.

Compound 18: Colorless high density liquid; 426.25 mg (71%); ^{1}H NMR (500 MHz, $CDCl_3$) δ 1.29 (t, J = 7.15 Hz, 3H), 2.03 (b, 1H), 3.45 (d, J = 16.25 Hz, 2H), 3.90 (d, J = 16.25 Hz, 2H), 4.21-4.25 (q, J = 7.15 Hz, 2H), 4.40 (s, 2H), 4.66 (s, 2H), 7.22 (s, 2H), 7.26 (s, 1H) ppm; ^{13}C NMR (125 MHz, $CDCl_3$) δ 14.2, 42.4, 47.0, 47.2, 62.3, 64.7, 65.1, 123.3, 124.6, 126.7, 138.5, 139.6, 140.9, 166.5, 170.6, 176.5 ppm; DEPT135 (125 MHz, $CDCl_3$) δ 14.2, 42.4, 47.0, 47.2,

62.3, 65.1, 123.3, 124.6, 126.7 ppm; IR (υ_{max}) 818, 862, 959, 1019, 1115, 1164, 1218, 1326, 1354, 1382, 1408, 1492, 1689, 1745, 2960, 2987, 3522 cm^{-1}; HRMS (ESI, Q-ToF) *m/z* calcd for $C_{16}H_{17}NNaO_5S$ $[M+Na]^+$ 358.0720, found 358.0719.

Compound 19: Colorless high density liquid; 122.0 mg (65%); ^{1}H NMR (500 MHz, $CDCl_3$) δ 1.27-1.30 (dt, J_1 = 0.75 Hz, J_2 = 7.15 Hz, 3H), 1.95 (b, 1H), 2.82 (t, J = 6.30 Hz, 2H), 3.42 (d, J = 16.30 Hz, 2H), 3.79-3.82 (dt, J_1 = 2.10 Hz, J_2 = 6.50 Hz, 2H), 3.85-3.90 (dd, J_1 = 9.35 Hz, J_2 = 16.25 Hz, 2H), 4.19-4.24 (dq, J_1 = 0.75 Hz, J_2 = 7.15 Hz, 2H), 4.38 (s, 2H), 7.08-7.17 (m, 3H) ppm; ^{13}C NMR (125 MHz, $CDCl_3$) δ 14.1, 39.0, 42.3, 47.0, 47.1, 62.2, 63.6, 64.6, 124.5, 125.1, 128.6, 137.1, 138.5, 139.4, 166.5, 170.6, 176.5 ppm; IR (υ_{max}) 820, 861, 959, 1020, 1114, 1163, 1217, 1326, 1354, 1382, 1407, 1493, 1616, 1689, 1745, 2950, 2983, 3525 cm^{-1}; HRMS (ESI, Q-ToF) *m/z* calcd for $C_{17}H_{20}NO_5S$ $[M+H]^+$ 350.1057, found 350.1057.

Compound 20: Colorless high density liquid; 42.0 mg (14%); ^{1}H NMR (500 MHz, $CDCl_3$) δ 1.24-1.27 (dt, J_1 = 1.60 Hz, J_2 = 7.15 Hz, 3H), 1.29 (t, J = 7.15 Hz, 3H), 2.18 (t, J = 2.50 Hz, 1H) 2.84-288 (td, J_1 = 2.55 Hz, J_2 = 16.85 Hz, 1H), 2.96-3.0 (dd, J_1 = 2.55 Hz, J_2 = 16.85 Hz, 1H), 3.27-3.30 (dd, J_1 = 4.95 Hz, J_2 = 13.90 Hz, 1H), 3.36-3.39 (dd, J_1 = 5.65 Hz, J_2 = 13.95 Hz, 1H), 3.44 (d, J = 16.40 Hz, 2H), 3.89 (d, J = 16.50 Hz, 1H), 3.91 (d, J = 16.55 Hz, 1H), 4.17-4.25 (m, J_1 = 0.75 Hz, J_2 = 7.15 Hz, 6H), 4.39 (s, 2H), 7.09-7.18 (m, 3H) ppm; ^{13}C NMR (125 MHz, $CDCl_3$) δ 14.2, 29.66, 29.71, 42.08, 42.11, 42.4, 43.2, 47.09, 47.14, 47.2, 62.2, 62.3, 63.5, 64.4, 64.5, 73.3, 77.8, 124.5, 126.6, 126.7, 130.2, 130.3, 133.67, 133.70, 138.8, 138.9, 139.6, 139.7, 166.1, 166.4, 169.4, 170.5, 175.1, 176.4 ppm; HRMS (ESI, Q-ToF) *m/z* calcd for $C_{26}H_{27}N_2O_8S_2$ $[M+H]^+$ 559.1208, found 559.1206.

Compound 21: Colorless high density liquid; 347.88 mg (70%); ^{1}H NMR (500 MHz, $CDCl_3$) δ 1.85 (b, 1H), 2.27 (t, J = 2.45 Hz, 1H), 3.41 (d, J = 16.25 Hz, 2H), 3.89-3.93 (dd, J_1 = 3.45 Hz, J_2 = 16.30 Hz, 2H), 4.42 (d, J = 2.45 Hz, 2H), 4.67 (s, 2H), 7.23-7.27 (m, 3H) ppm; ^{13}C NMR (125 MHz, $CDCl_3$) δ 31.1, 47.1, 47.3, 64.7, 65.2, 72.4, 76.3, 123.3, 124.7, 126.8, 138.4, 139.5, 140.9, 170.0, 175.8 ppm; HRMS (ESI, Q-ToF) *m/z* calcd for $C_{15}H_{14}NO_3S$ $[M+H]^+$ 288.0691, found 288.0692.

Hydrolysis of compound 18 to compound 22: A mixture of acetate **18** (250 mg, 0.75 mmol), glacial AcOH (10 mL), and HCl (12 N, 3 mL) was refluxed for 5 h. After evaporation in vacuo, water was added and extracted with EtOAc. The combined organic phases were washed with brine, dried over anhydrous Na_2SO_4, filtered and concentrated. The crude residue was then purified by column chromatography on silica gel with EtOAc/petroleum ether 50:50 to 80:20 gave compound **22** as a wheatish solid (206.19 mg, 90% yield). MP 137.5-139.5 ^oC; ^{1}H NMR

(400 MHz, $CDCl_3$) δ 3.47 (d, J = 16.45Hz, 2H), 3.90-3.95 (dd, J_1 = 4.04 Hz, J_2 = 16.53 Hz, 2H), 4.47 (s, 2H), 4.58 (s, 2H), 6.87 (b, 2H), 7.23-7.29 (m, 3H) ppm; ^{13}C NMR (100 MHz, $CDCl_3$) δ 42.1, 46.2, 47.2, 64.6, 124.9, 128.5, 137.5, 139.5, 139.8, 170.4, 171.4, 176.3 ppm; IR (υ_{max}) 823, 960, 985, 1025, 1117, 1165, 1230, 1327, 1384, 1409, 1492, 1682, 1731, 2548, 2626, 2751, 2952, 3429 cm^{-1}; HRMS (ESI, Q-ToF) *m/z* calcd for $C_{16}H_{17}NNaO_5S$ $[M+Na]^+$ 358.0720, found 358.0719.

Synthesis of compound 24: The monoalkynylated precursor **21** (100 mg, 0.35 mmol) was dissolved in *t*-BuOH/H_2O 3:3 (mL) and the *p*-nitrophenyl azide (**23**, 62.90 mg, 0.38 mmol), $Cu(OAc)_2$ (6.33 mg, 0.03 mmol) and sodium ascorbate (13.81 mg, 0.07 mmol) were added. The resulting mixture was stirred at rt until TLC indicated completion of reaction. The mixture was diluted with ethyl acetate and washed with aq. NH_4OH (0.2%) and brine. The aqueous phases were extracted with ethyl acetate (2 × 10 mL). The combined organic extracts were dried over Na_2SO_4 and concentrated in vacuo. The crude product was purified by silica gel column chromatography (70–30% EtOAc/petroleum ether) to deliver the triazole adduct **24** as yellow solid (125.71 mg, 80% yield). MP 188.5-190.5 ^oC; ^1H NMR (500 MHz, DMSO) δ 3.48-3.52 (dd, J_1 = 5.55 Hz, J_2 = 16.50 Hz, 2H), 3.71-3.75 (dd, J_1 = 5.85 Hz, J_2 = 16.50 Hz, 2H), 4.47 (s, 2H), 4.94 (s, 2H), 5.16 (b, 1H), 7.16-7.24 (m, 3H), 8.23 (d, J = 9.10 Hz, 2H), 8.45 (d, J = 9.10 Hz, 2H), 9.00 (s, 1H) ppm; ^{13}C NMR (125 MHz, $CDCl_3$) δ 36.6, 46.0, 46.2, 62.8, 63.9, 120.7, 122.2, 122.5, 123.9, 125.6, 125.7, 137.8, 139.4, 140.7, 141.9, 143.1, 146.8, 170.2, 176.0 ppm; HRMS (ESI, Q-ToF) *m/z* calcd for $C_{21}H_{17}N_5NaO_5S$ $[M+Na]^+$ 474.0843, found 474.0847.

^{1}H NMR (500 MHz, CDCl$_3$) of compound **5b**

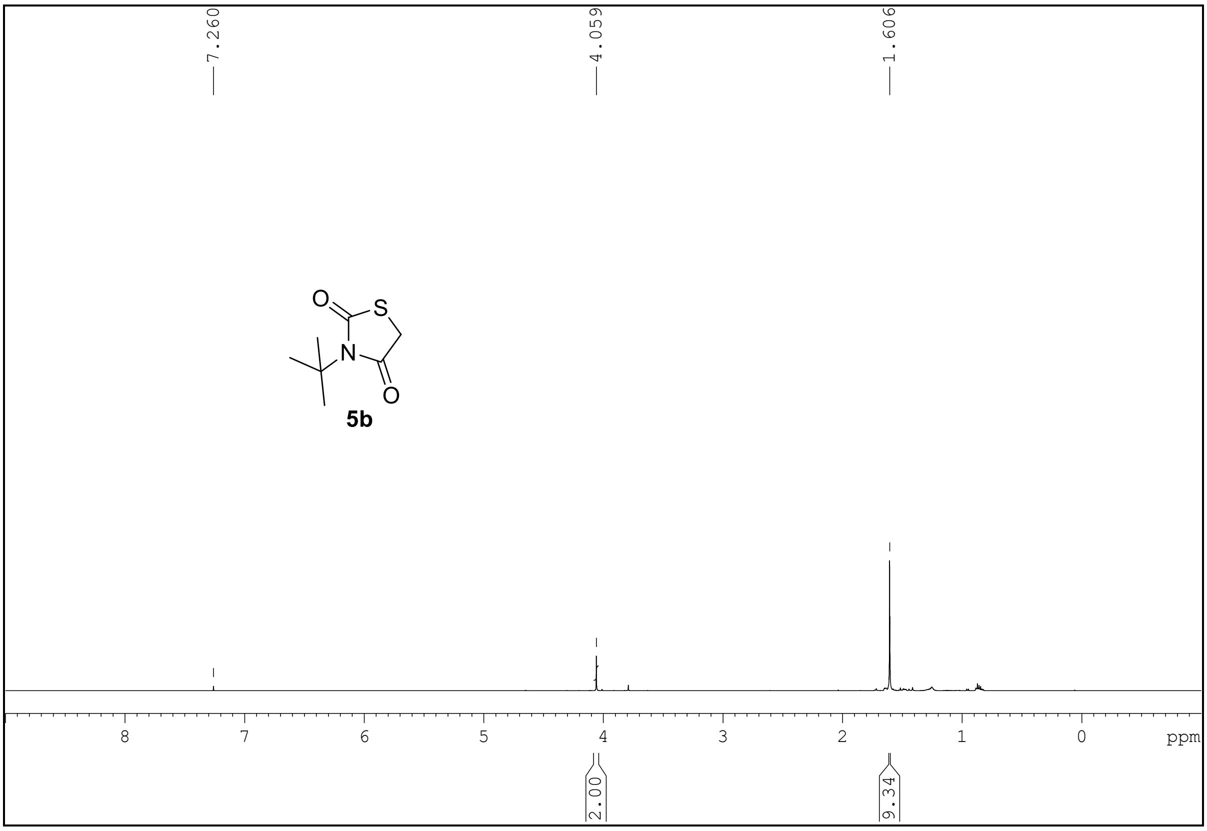

^{13}C NMR (125 MHz, CDCl$_3$) of compound **5b**

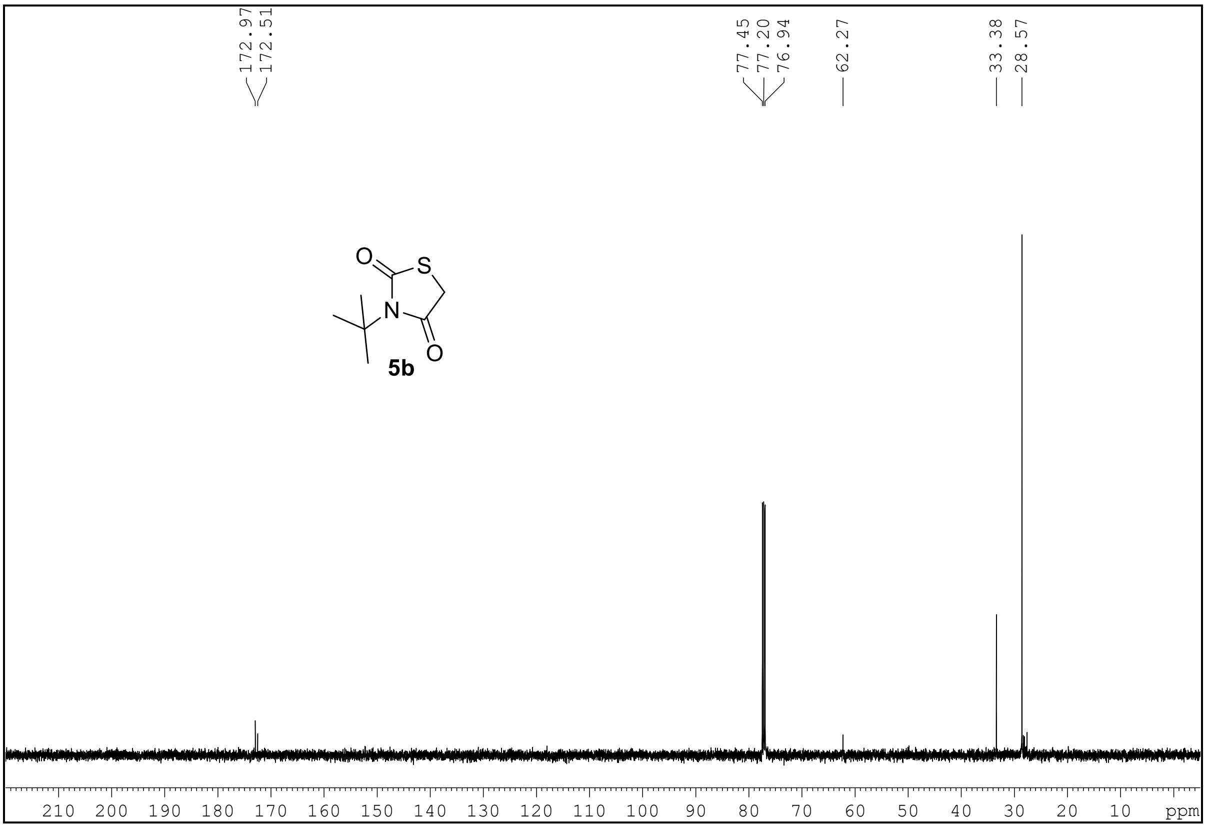

^{1}H NMR (500 MHz, $CDCl_3$) of compound **7a**

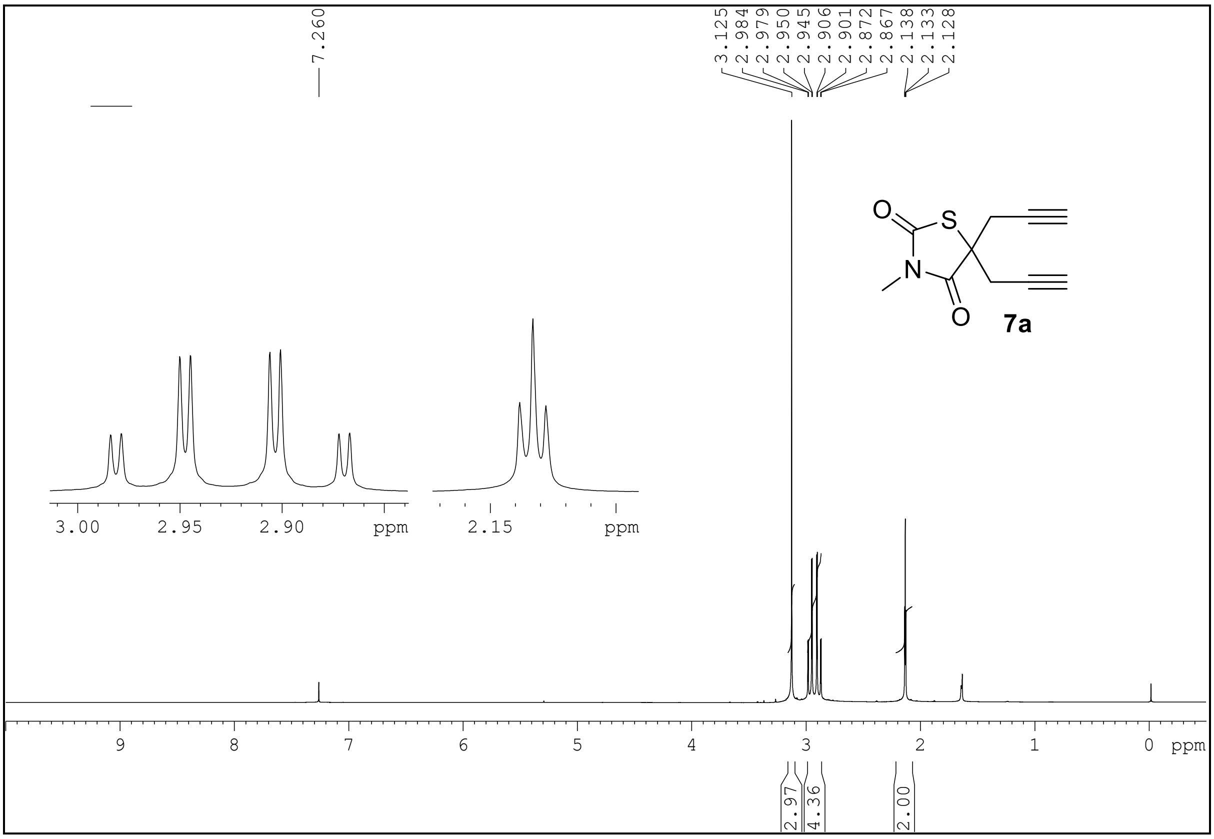

^{13}C NMR (125 MHz, $CDCl_3$) of compound **7a**

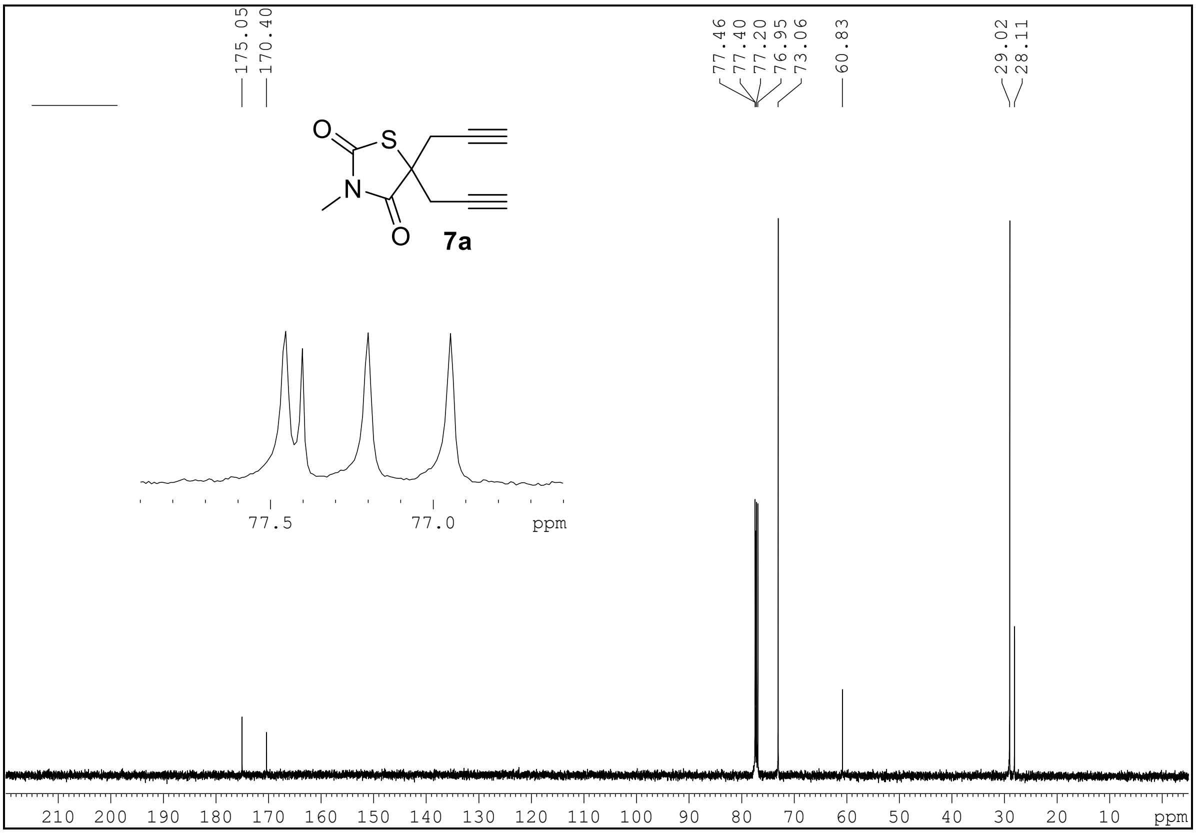

^{1}H NMR (500 MHz, $CDCl_3$) of compound **7b**

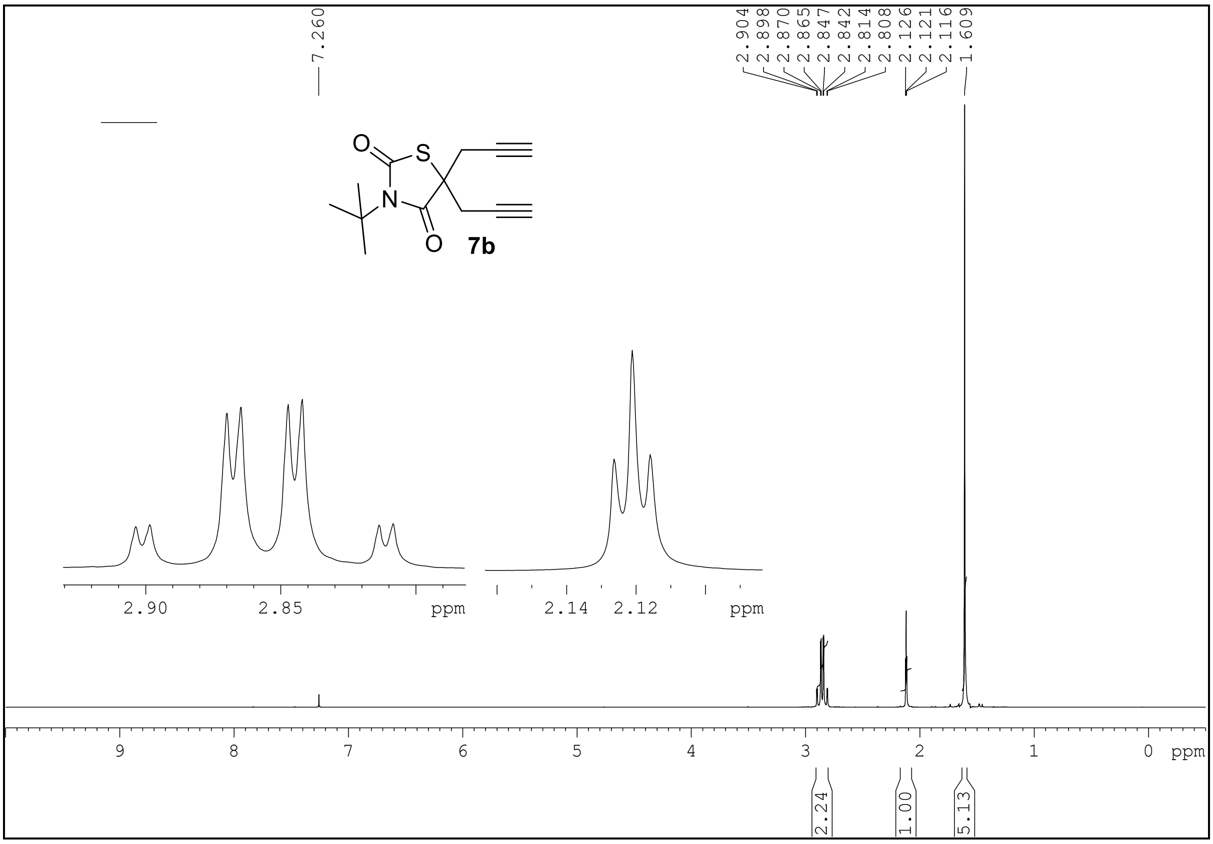

^{13}C NMR (125 MHz, $CDCl_3$) of compound **7b**

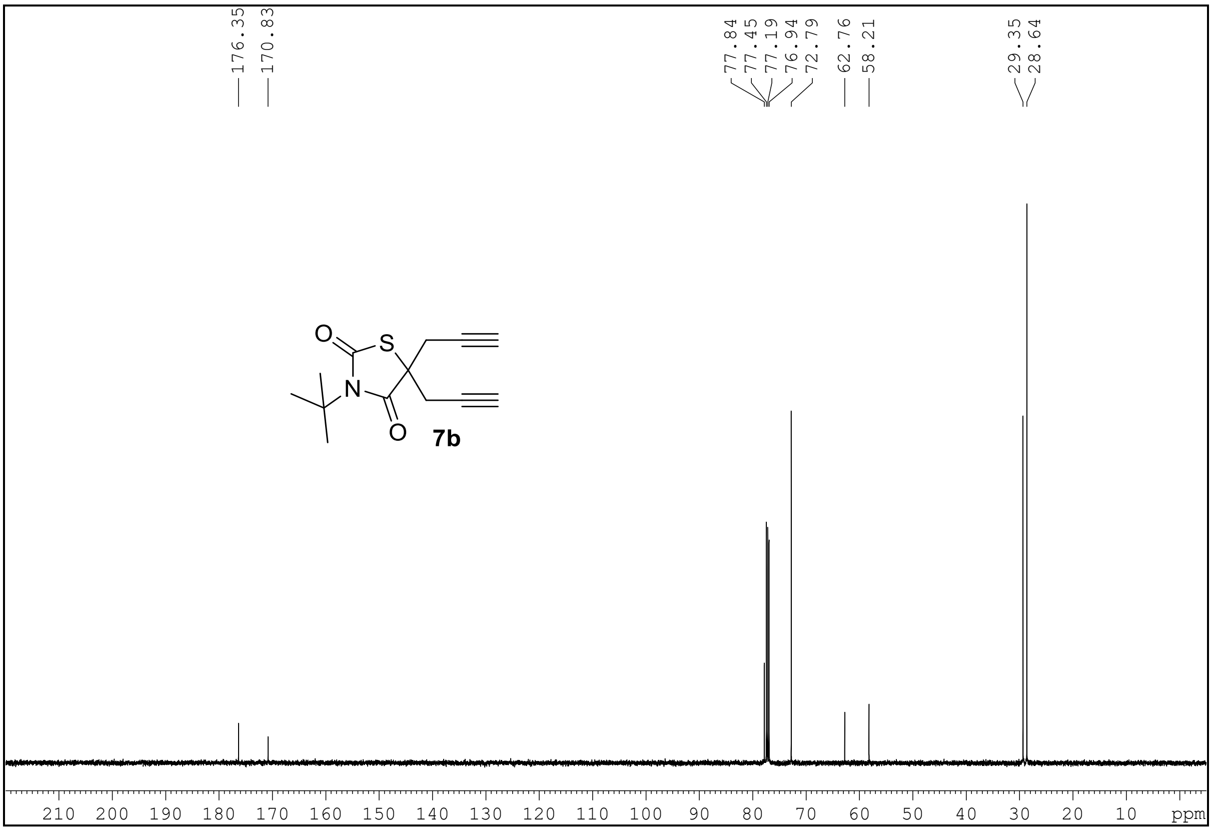

^{1}H NMR (500 MHz, $CDCl_3$) of compound **7c**

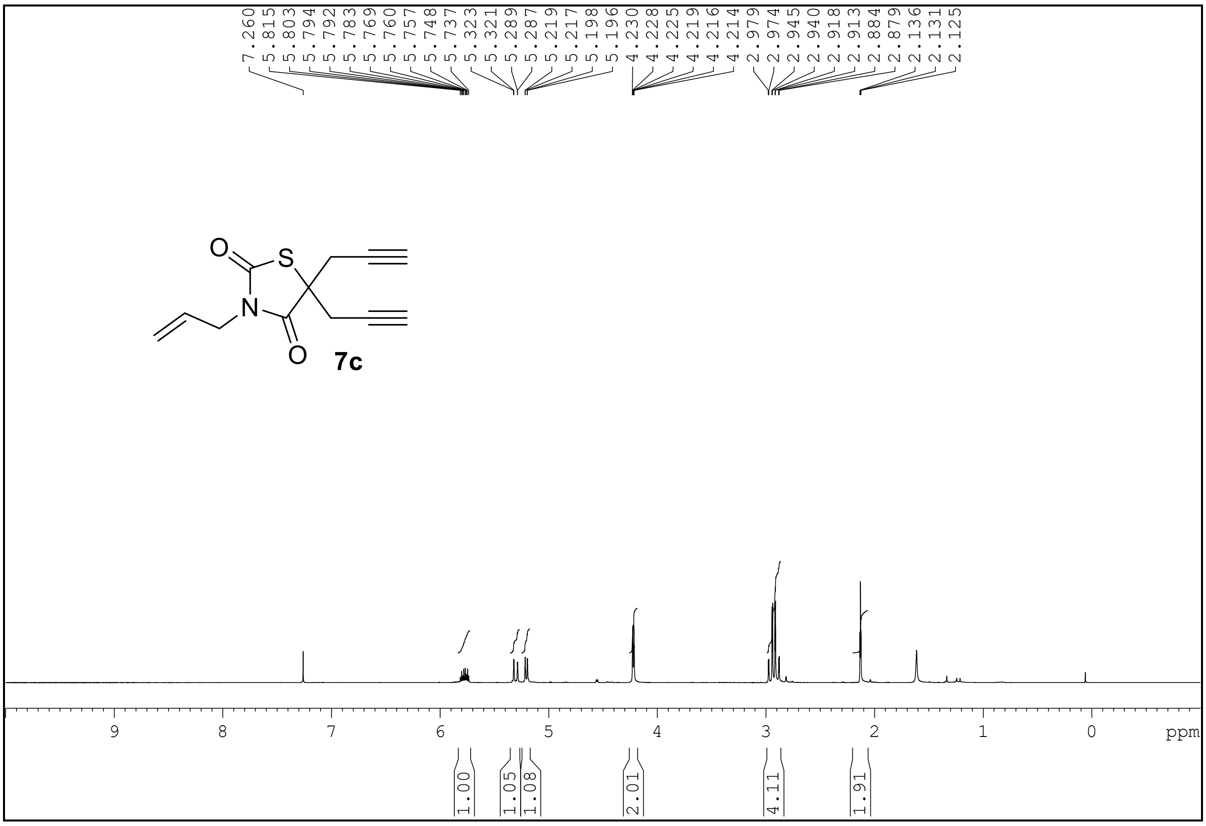

^{13}C NMR (125 MHz, $CDCl_3$) of compound **7c**

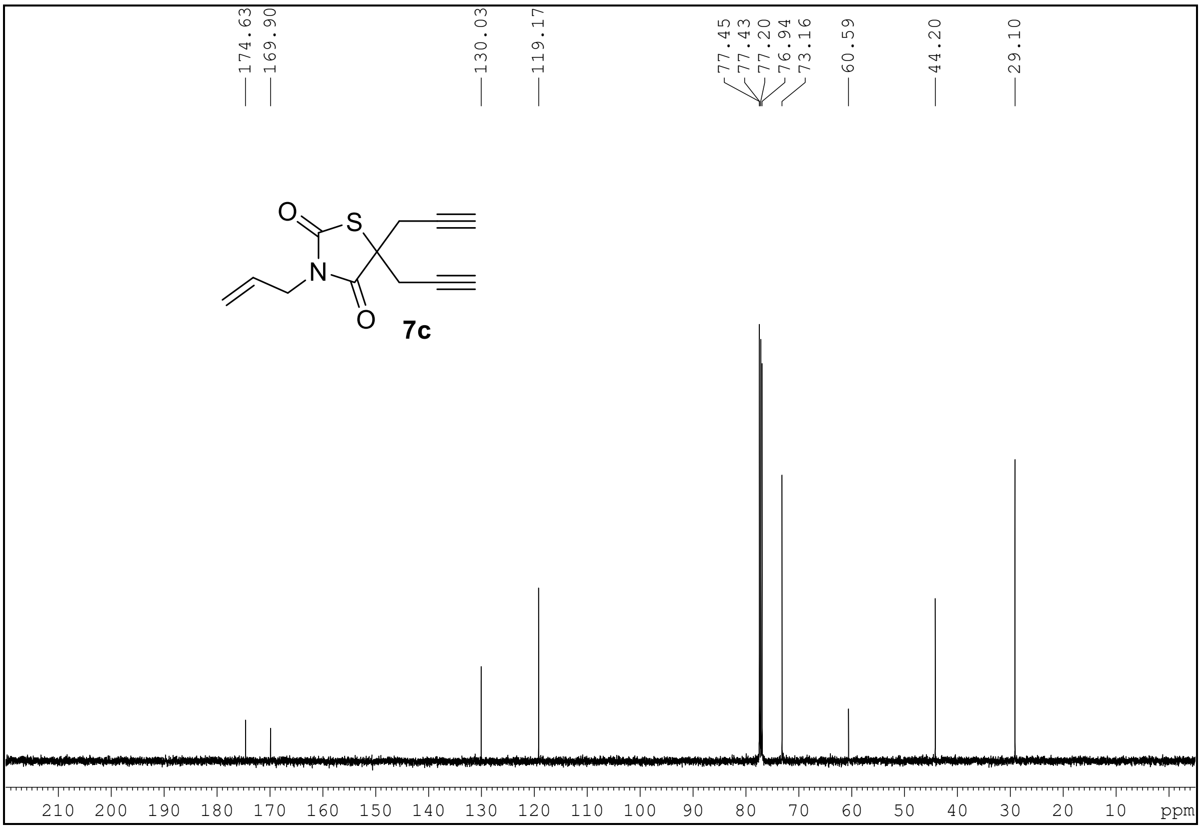

^{1}H NMR (500 MHz, $CDCl_3$) of compound **7d**

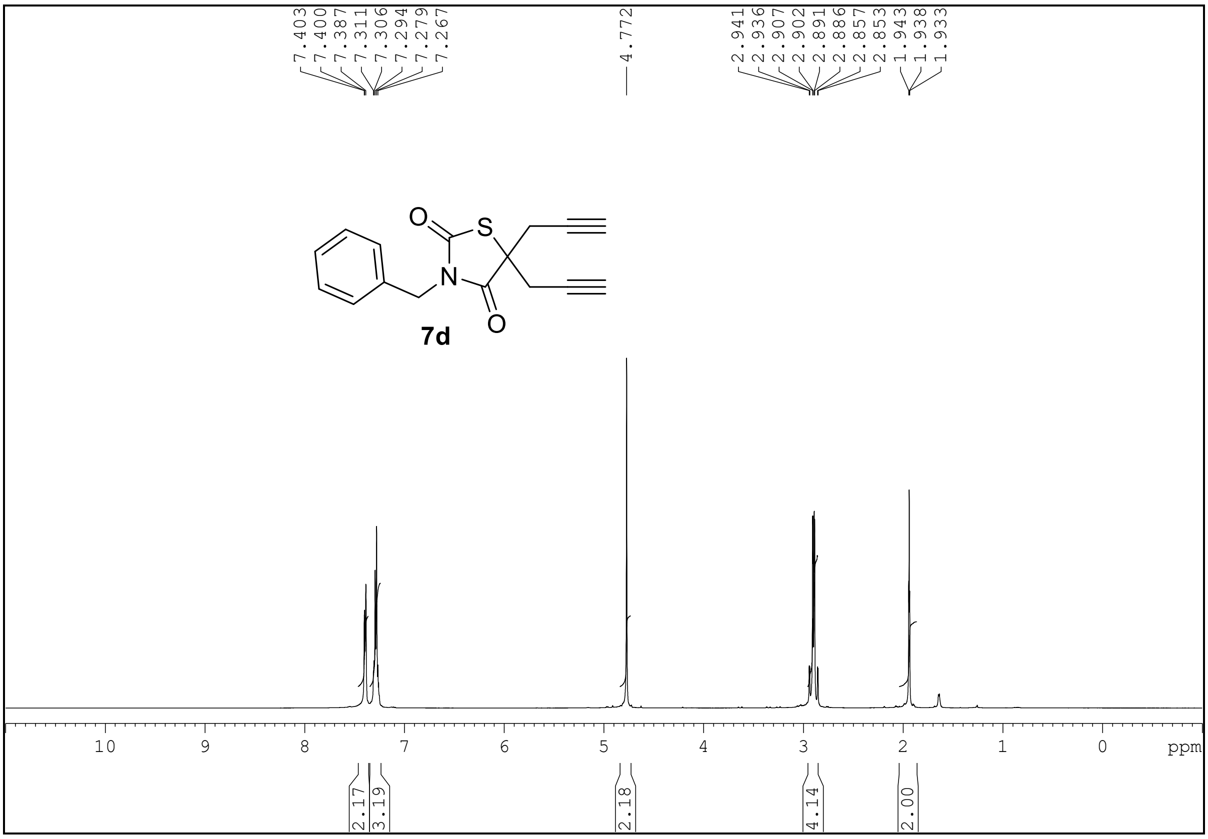

^{13}C NMR (125 MHz, $CDCl_3$) of compound **7d**

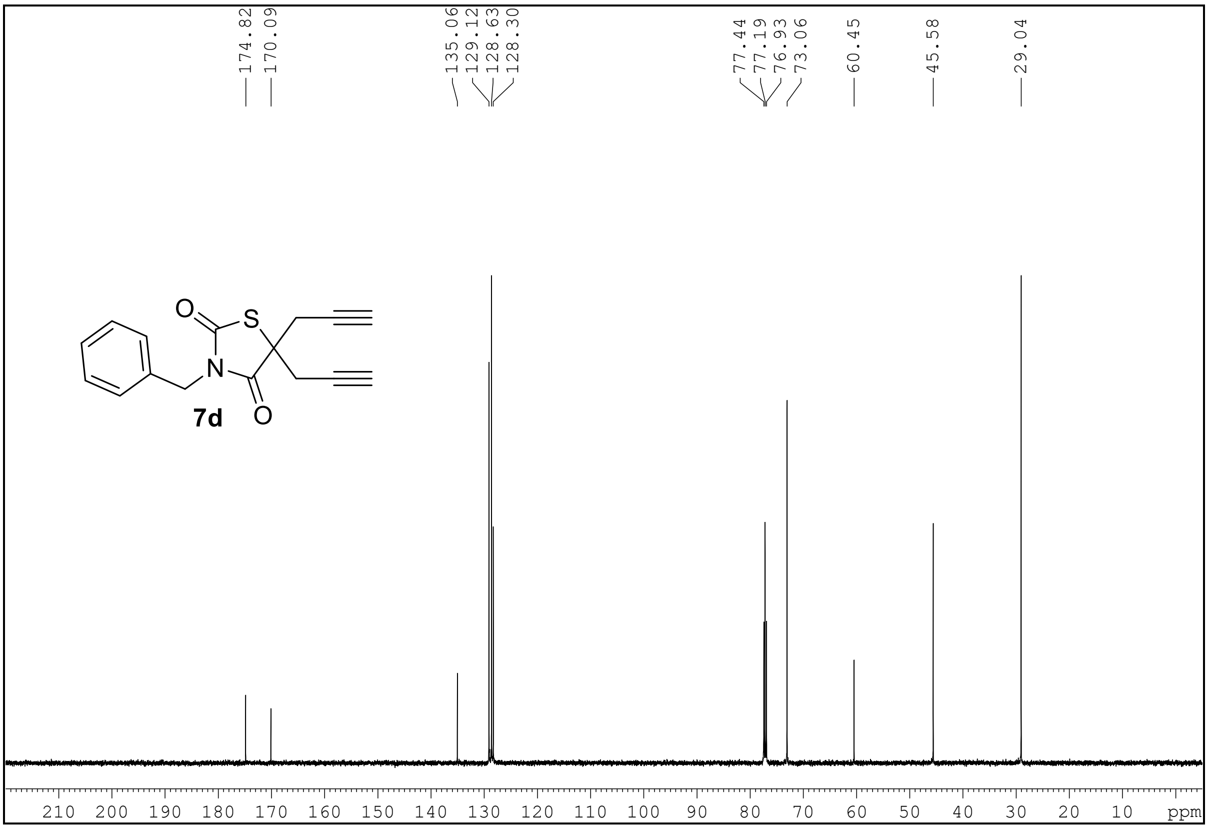

DEPT NMR (125 MHz, $CDCl_3$) of compound **7d**

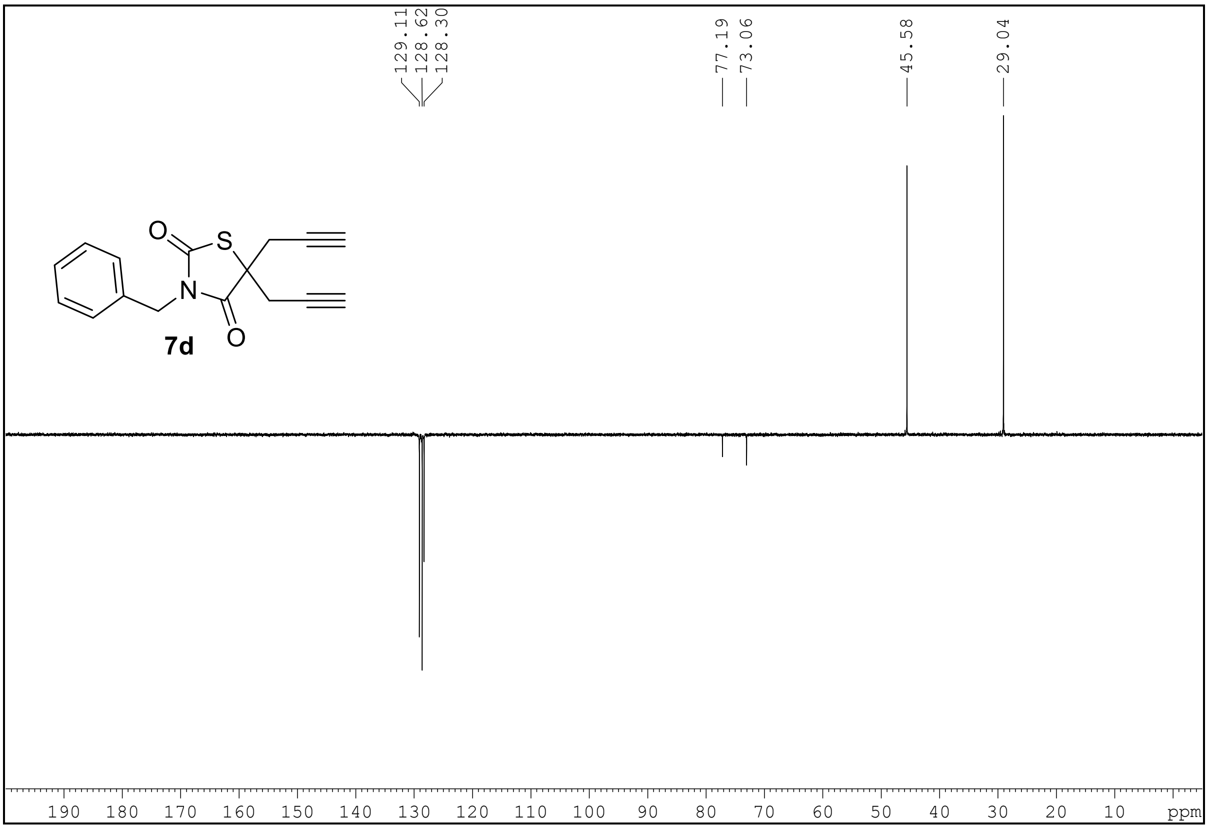

^{1}H NMR (500 MHz, $CDCl_3$) of compound **8a**

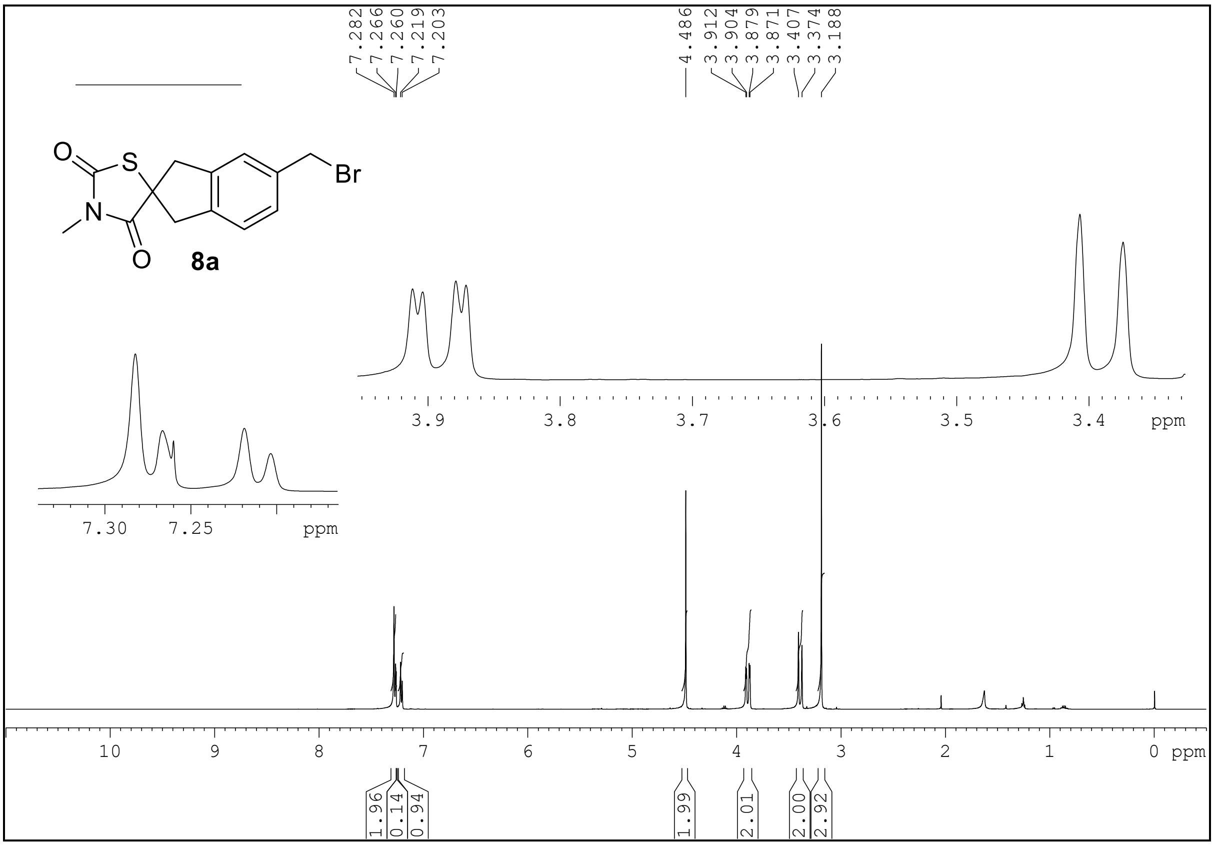

^{13}C NMR (125 MHz, $CDCl_3$) of compound **8a**

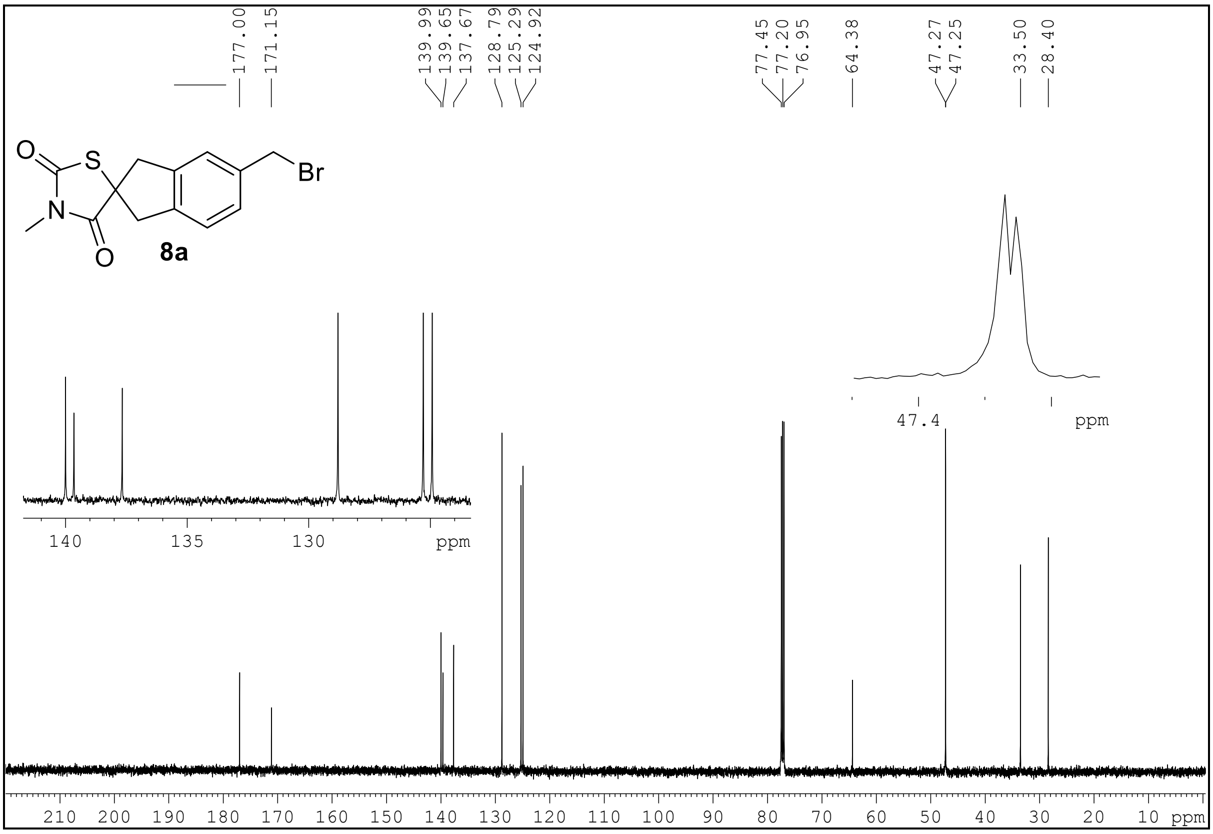

^{1}H NMR (400 MHz, $CDCl_3$) of compound **8b**

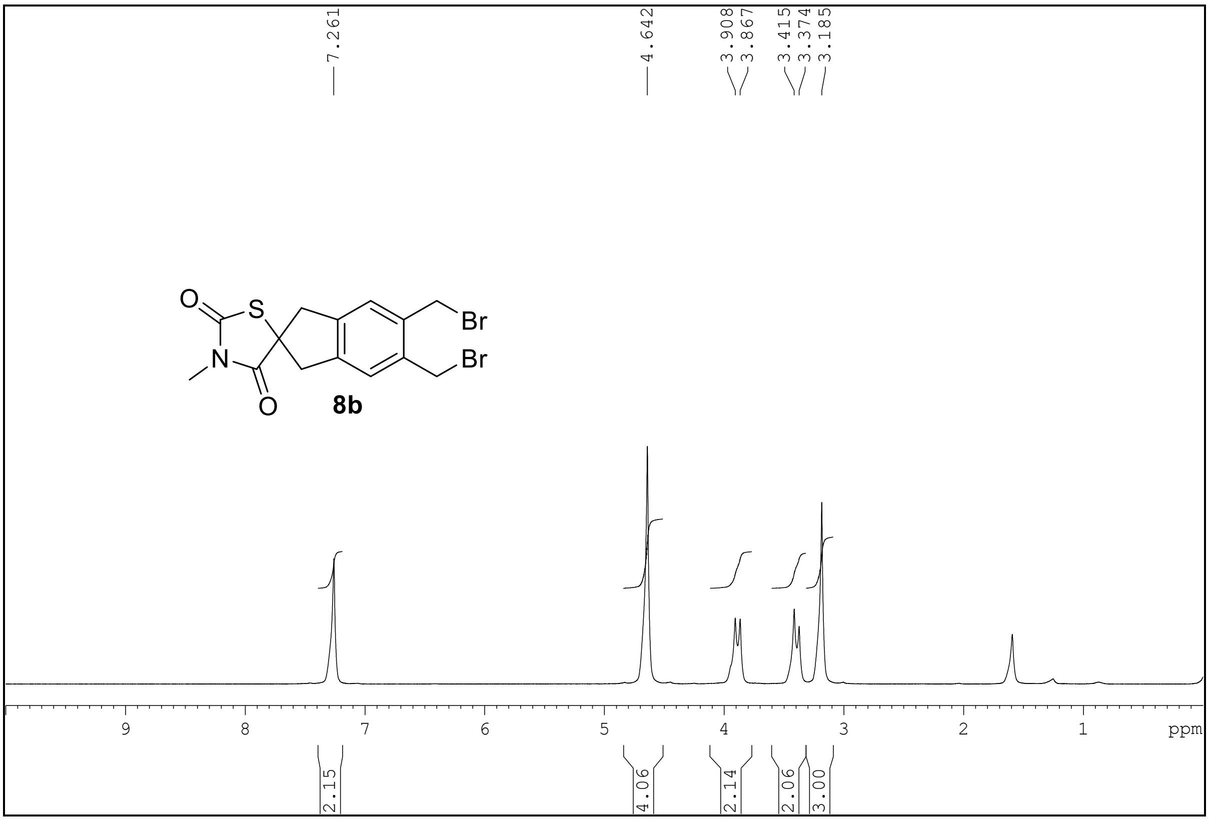

^{13}C NMR (125 MHz, $CDCl_3$) of compound **8b**

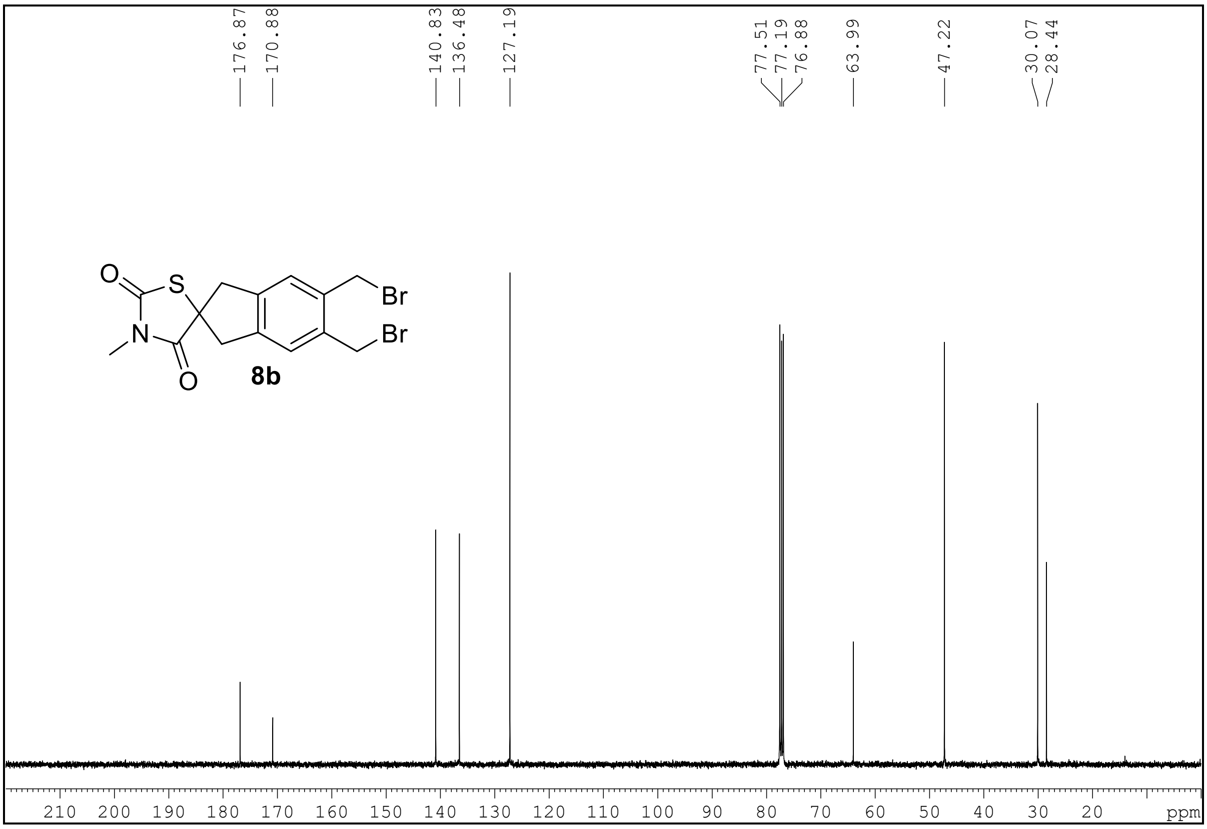

^{1}H NMR (500 MHz, $CDCl_3$) of compound **8c**

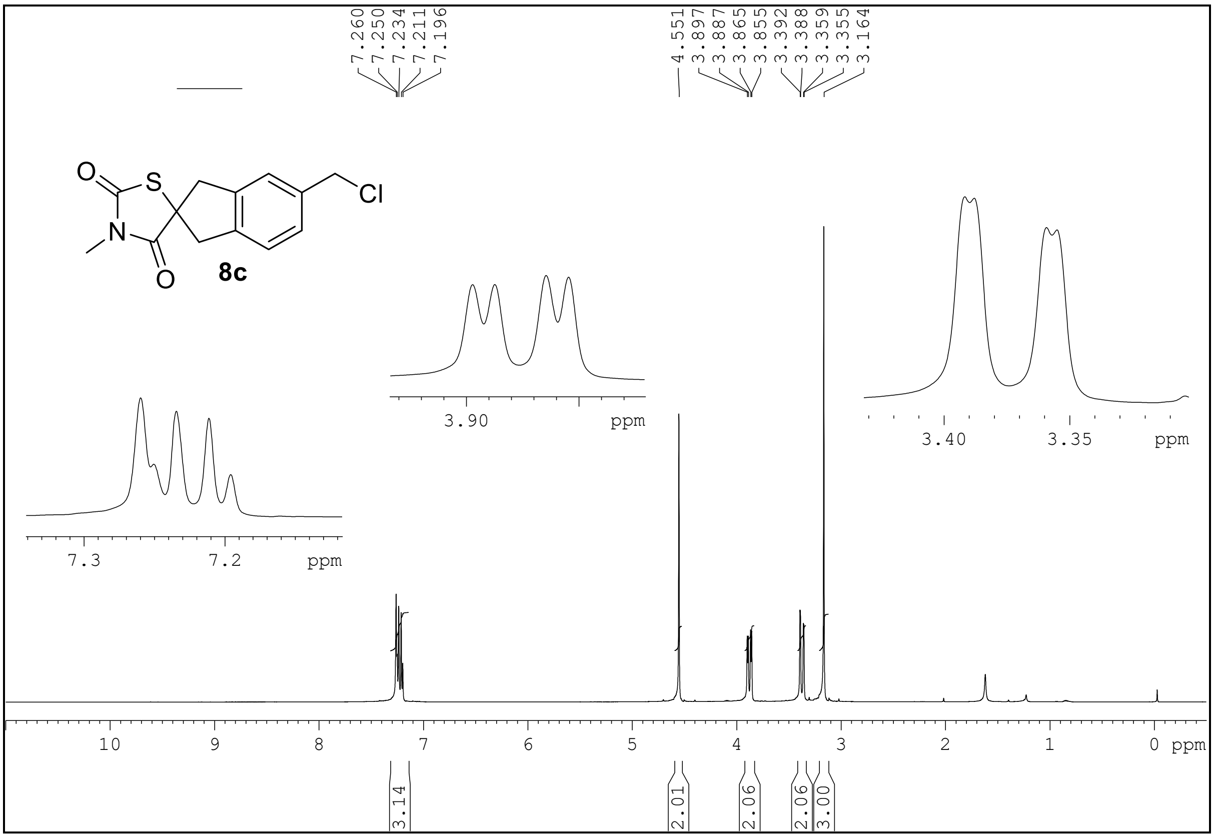

^{13}C NMR (125 MHz, $CDCl_3$) of compound **8c**

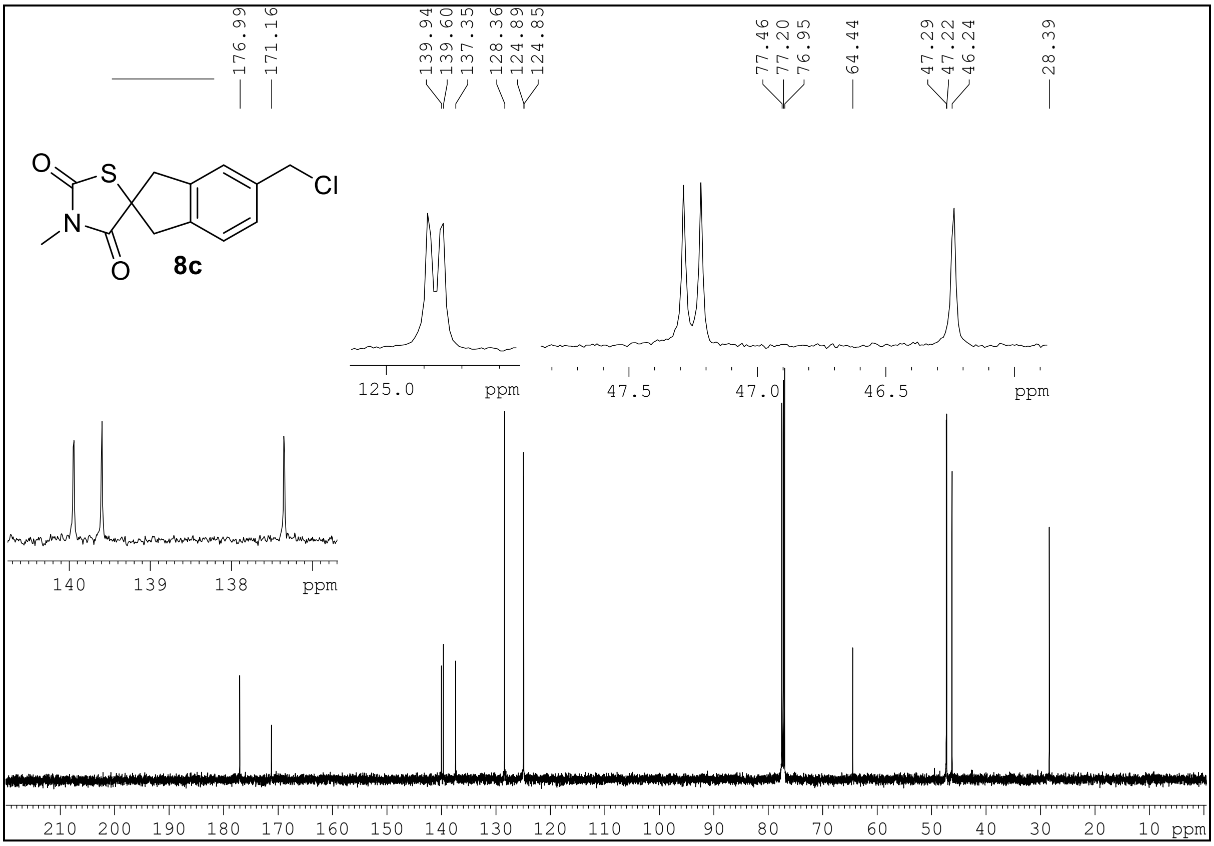

^{1}H NMR (400 MHz, $CDCl_3$) of compound **8d**

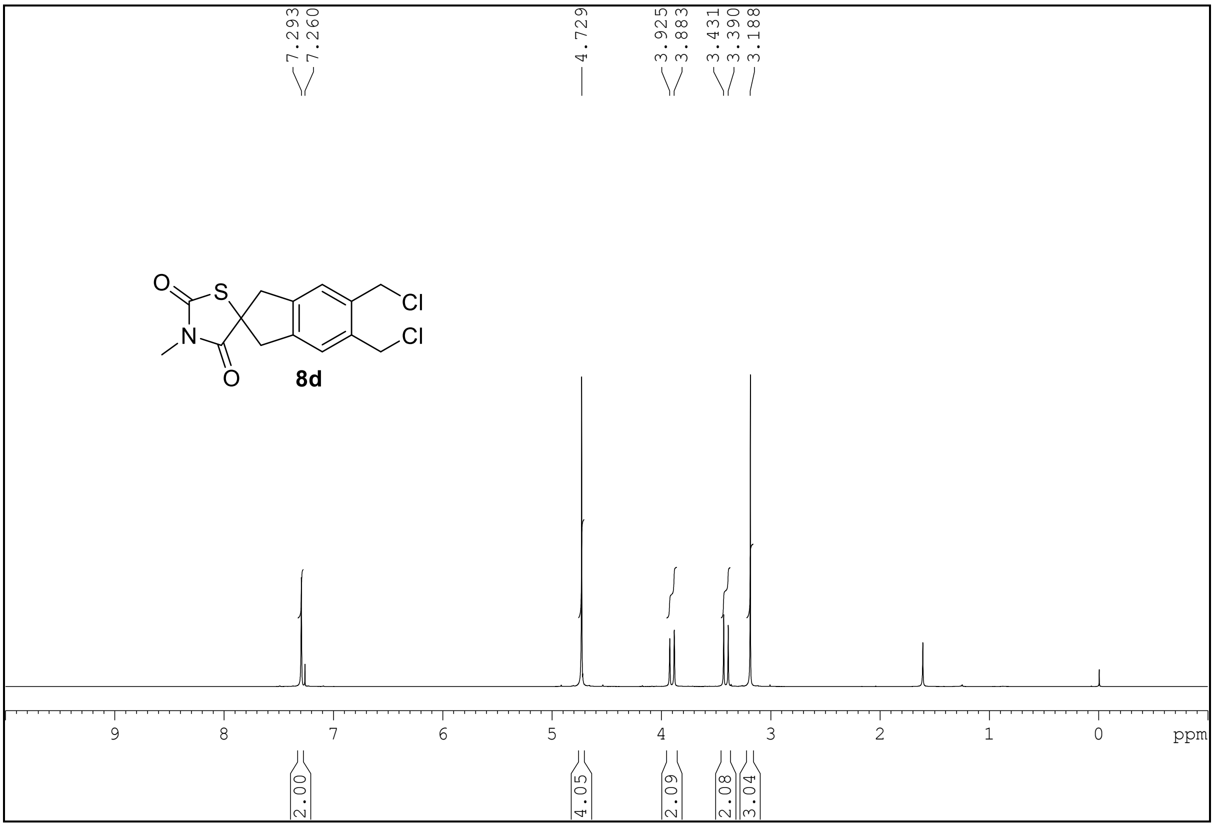

^{13}C NMR (100 MHz, $CDCl_3$) of compound **8d**

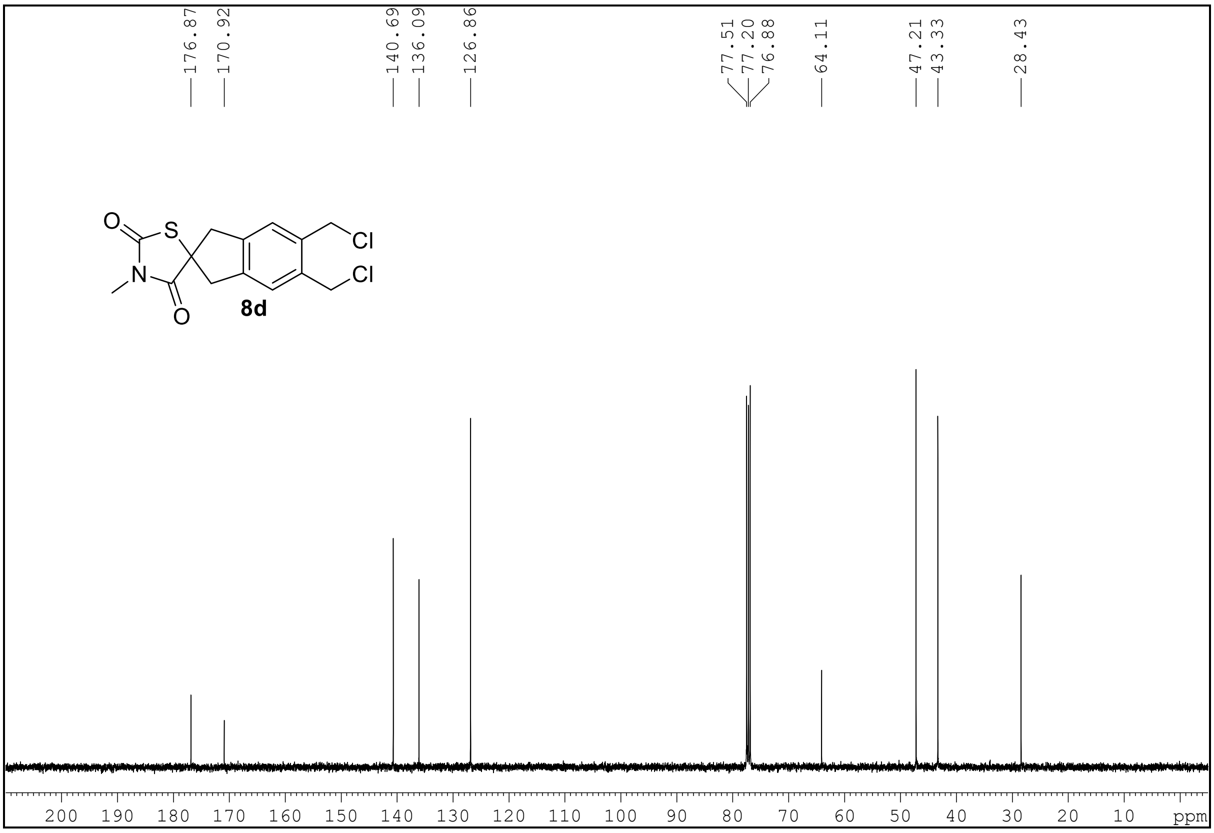

^{1}H NMR (400 MHz, $CDCl_3$) of compound **10a**

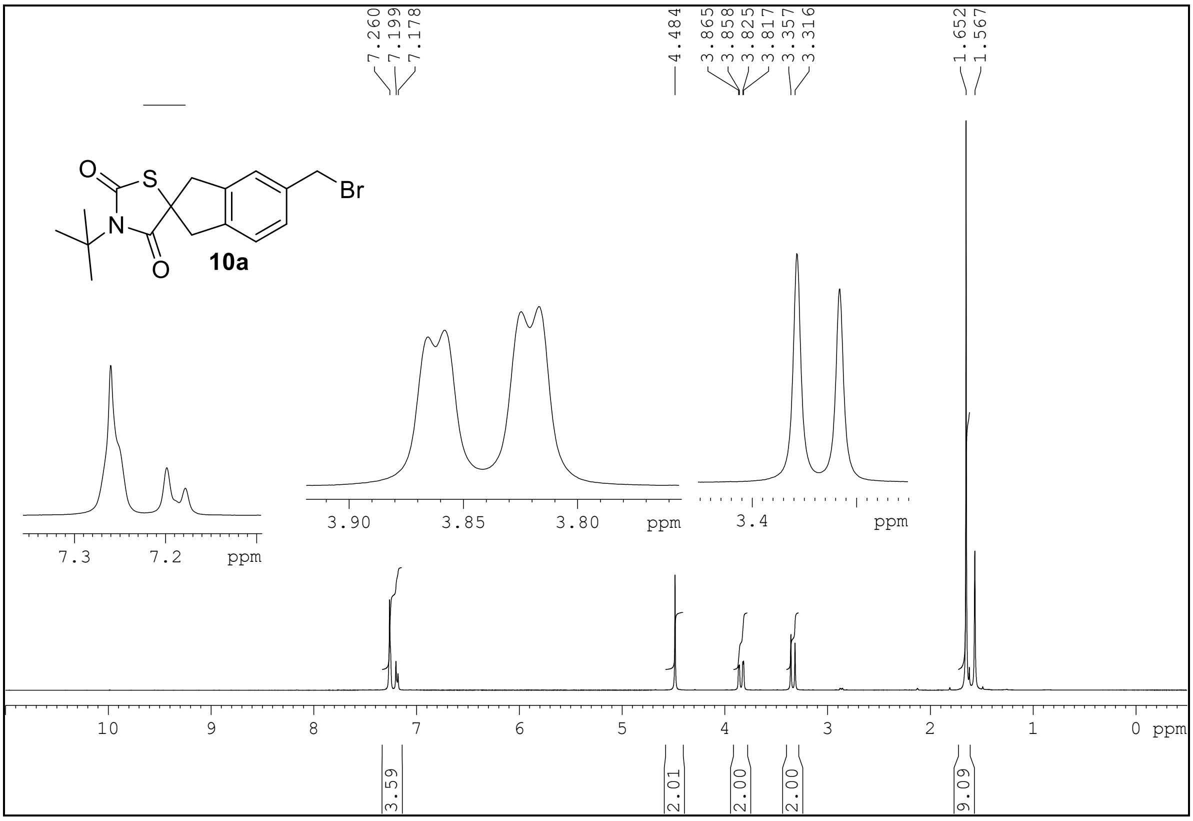

^{13}C NMR (100 MHz, $CDCL_3$) of compound **10a**

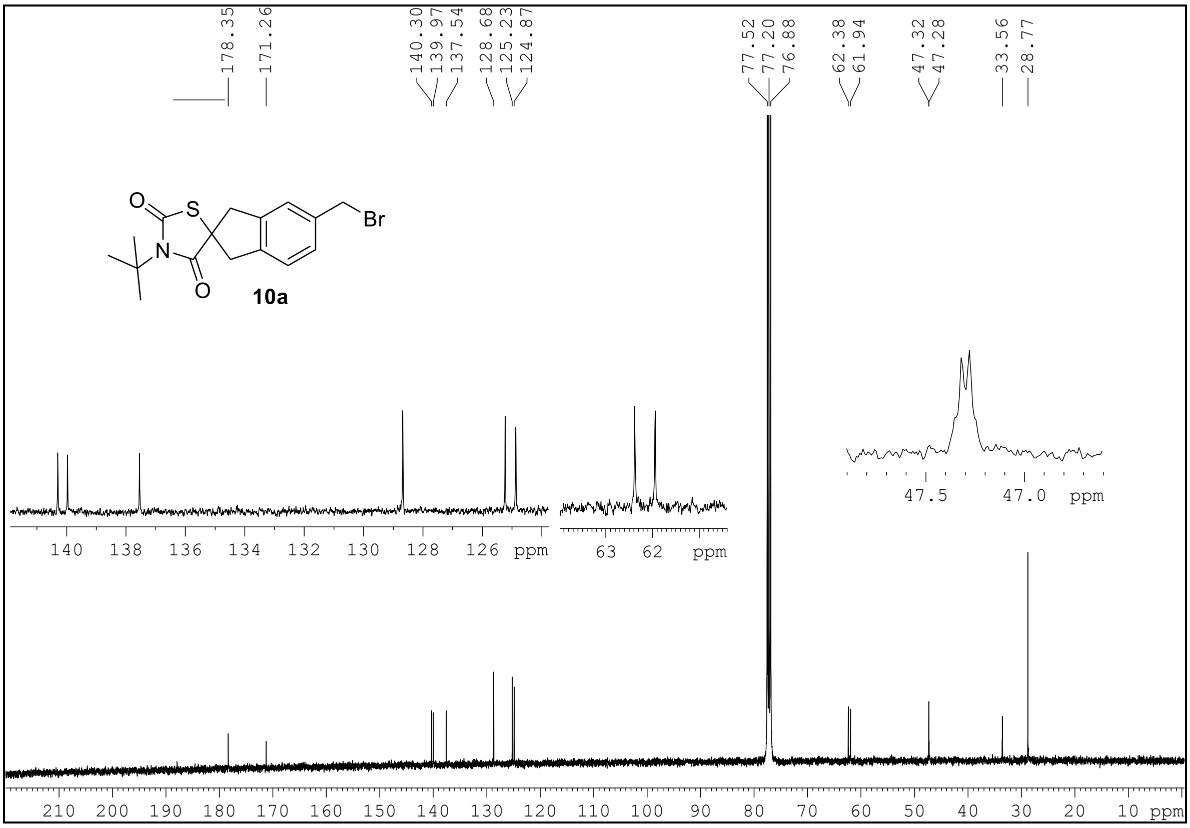

^{1}H NMR (500 MHz, $CDCl_3$) of compound **10b**

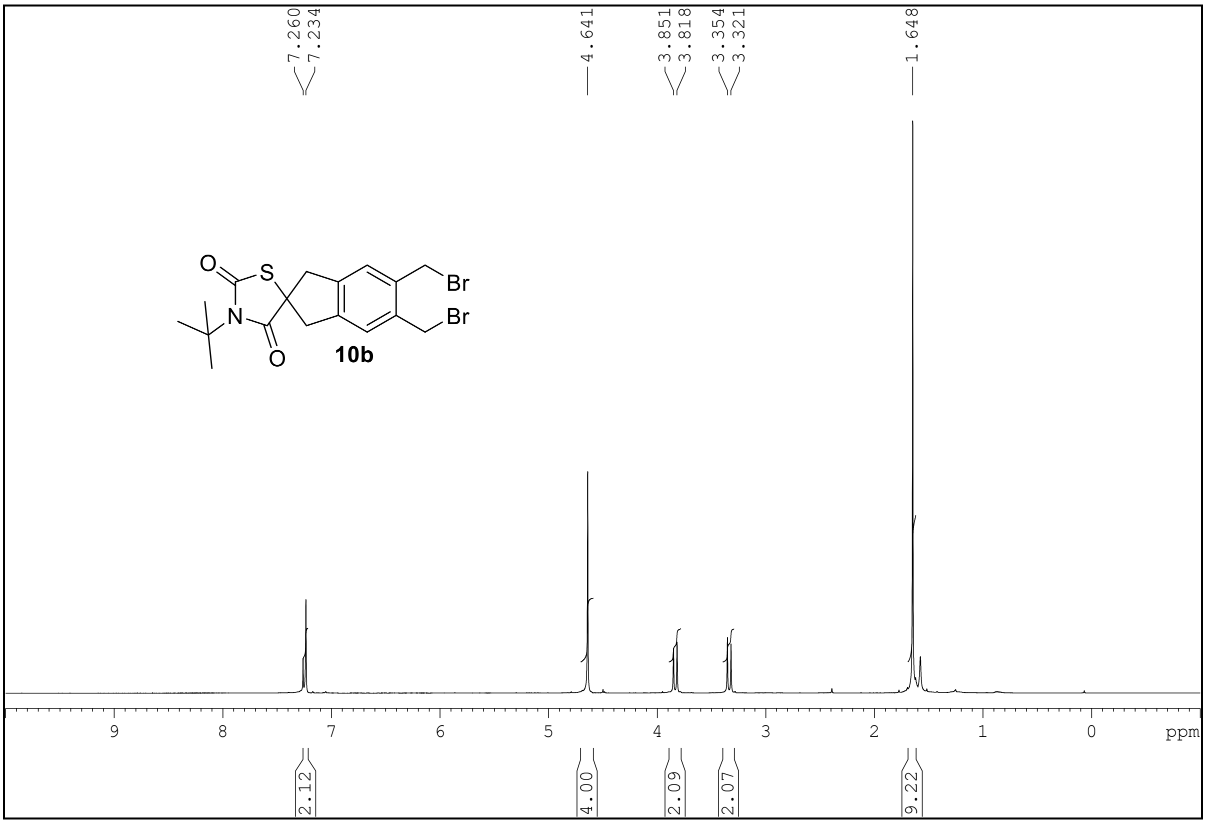

^{13}C NMR (125 MHz, $CDCl_3$) of compound **10b**

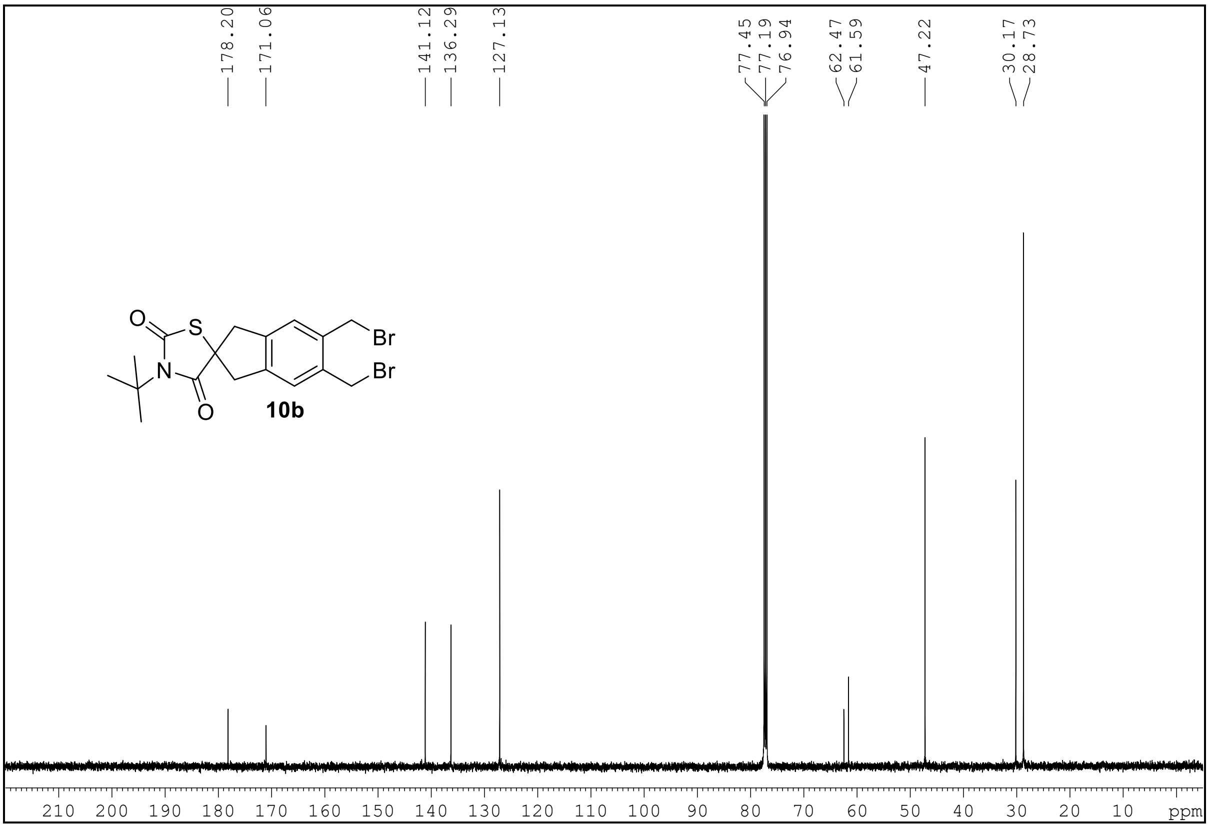

^{1}H NMR (500 MHz, $CDCl_3$) of compound **10c**

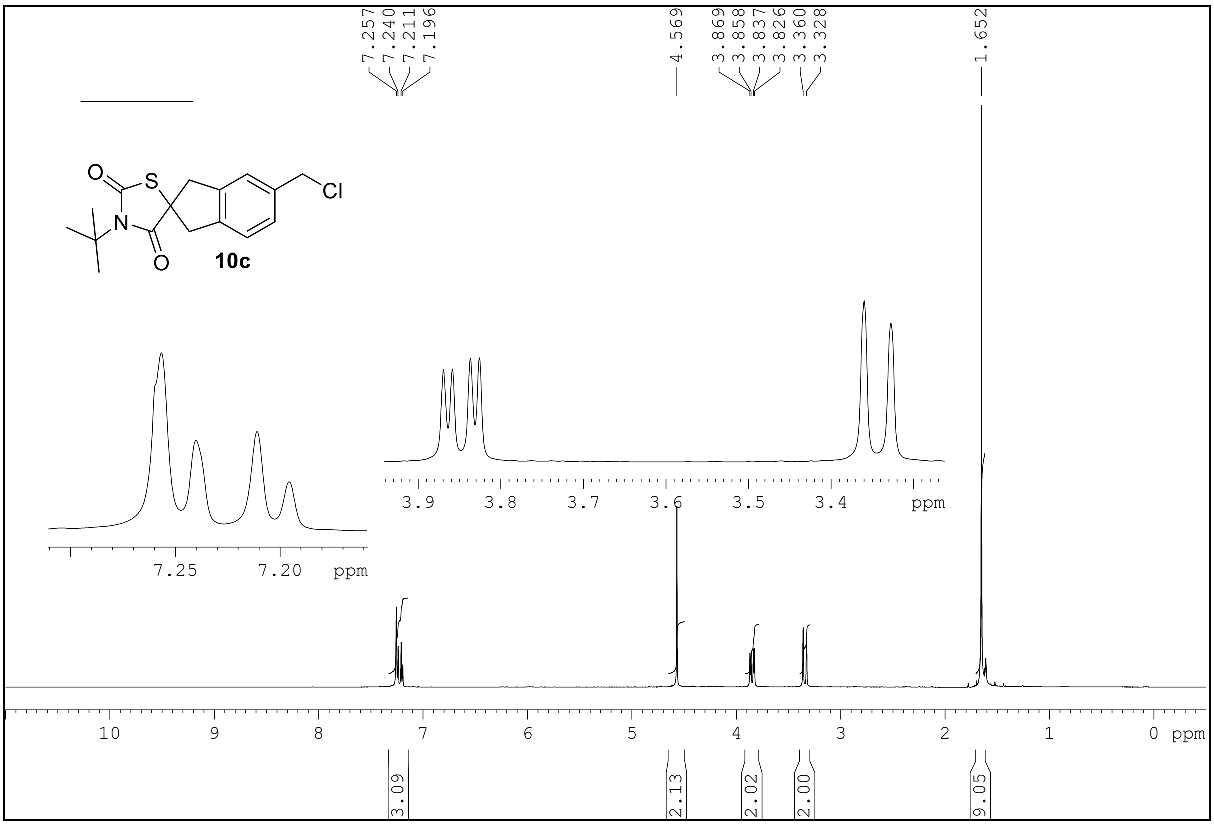

^{13}C NMR (125 MHz, $CDCl_3$) of compound **10c**

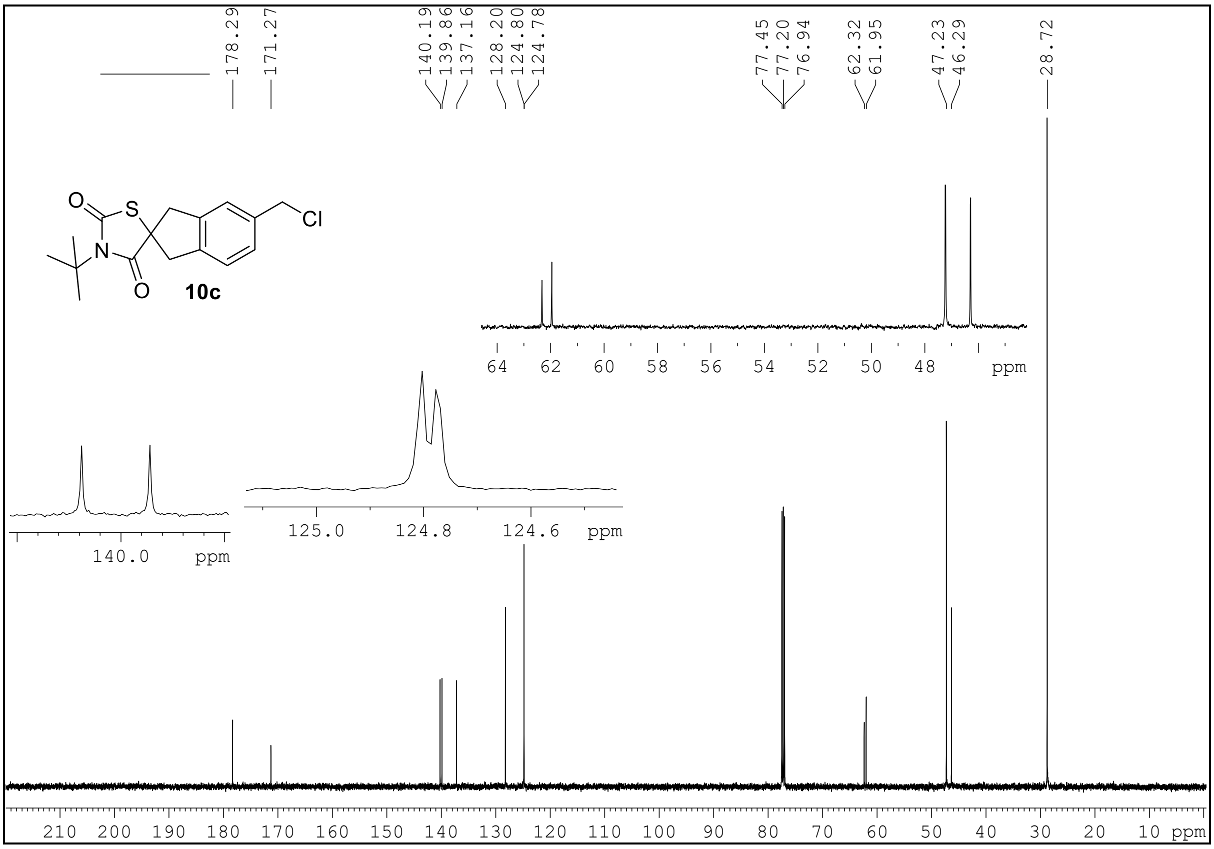

^{1}H NMR (500 MHz, $CDCl_3$) of compound **10d**

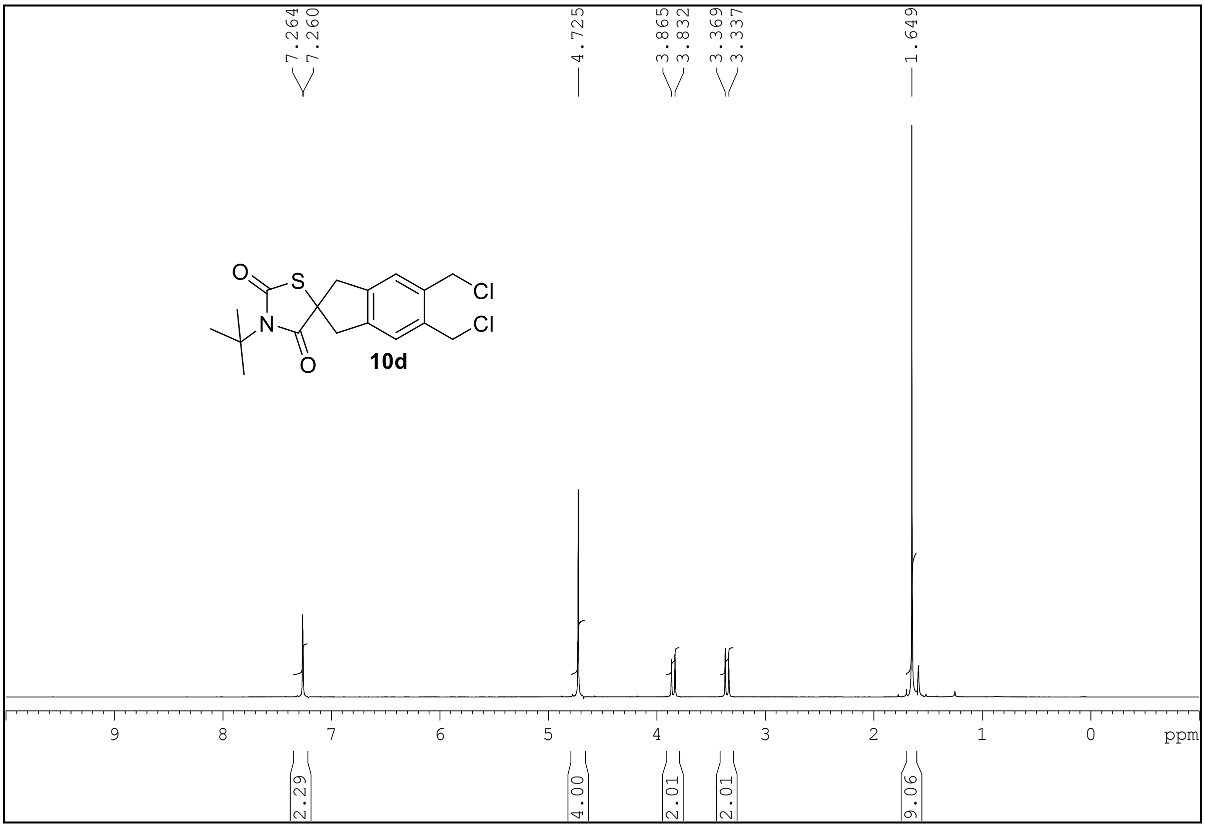

^{13}C NMR (125 MHz, $CDCl_3$) of compound **10d**

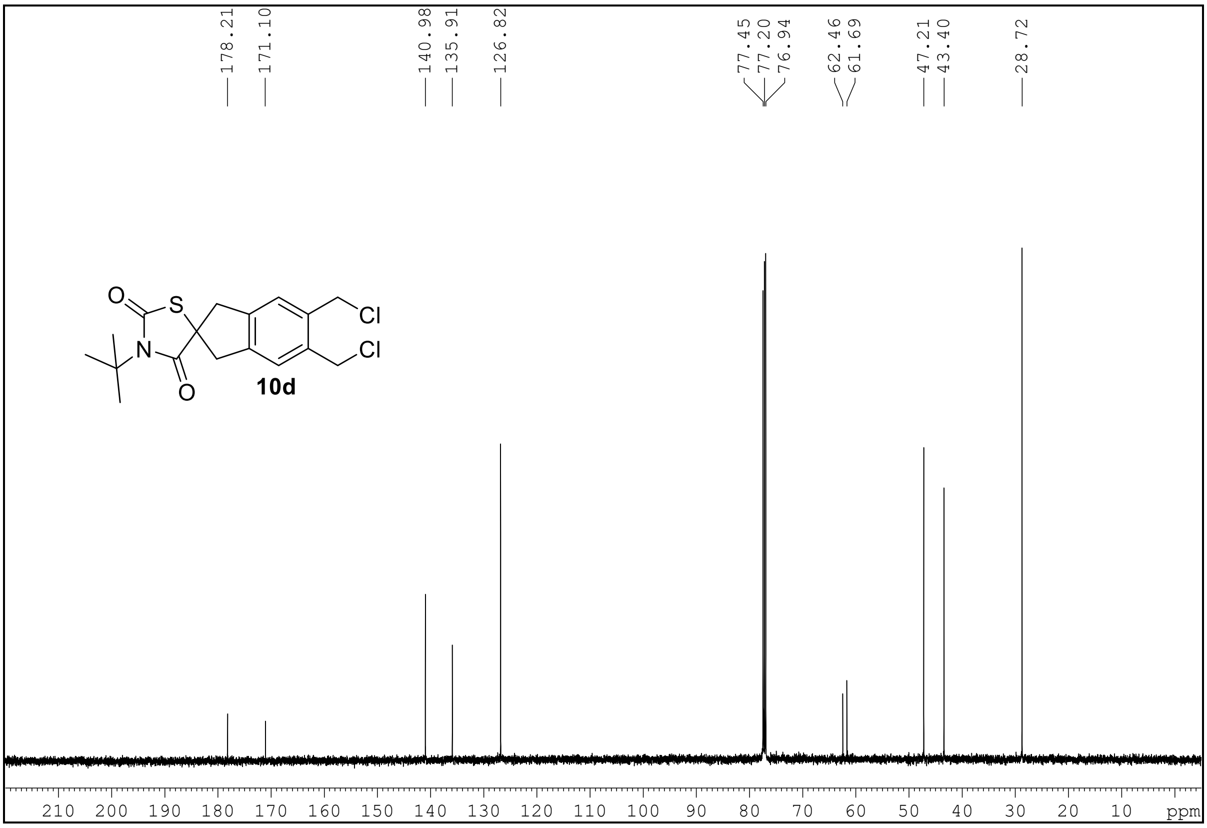

^{1}H NMR (500 MHZ, CDCl3) of compound **11**

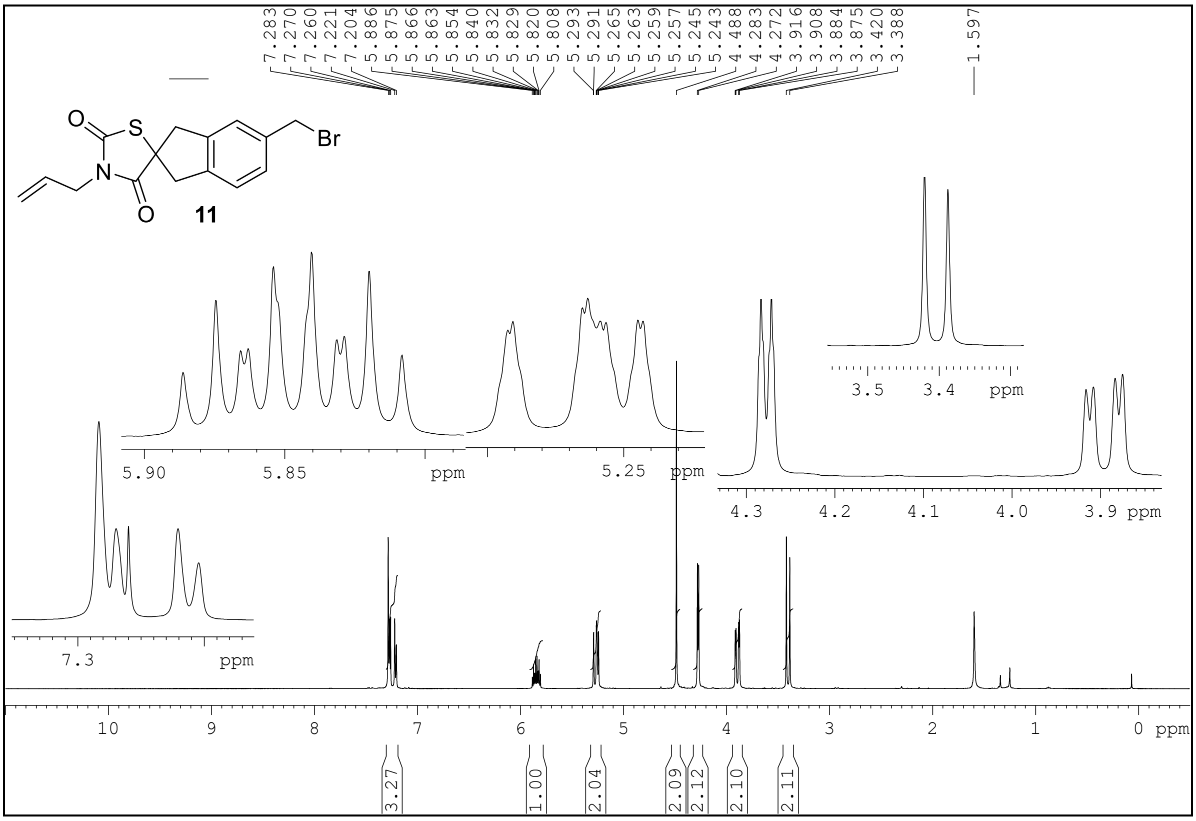

^{13}C NMR (125 MHz, CDCl3) of compound **11**

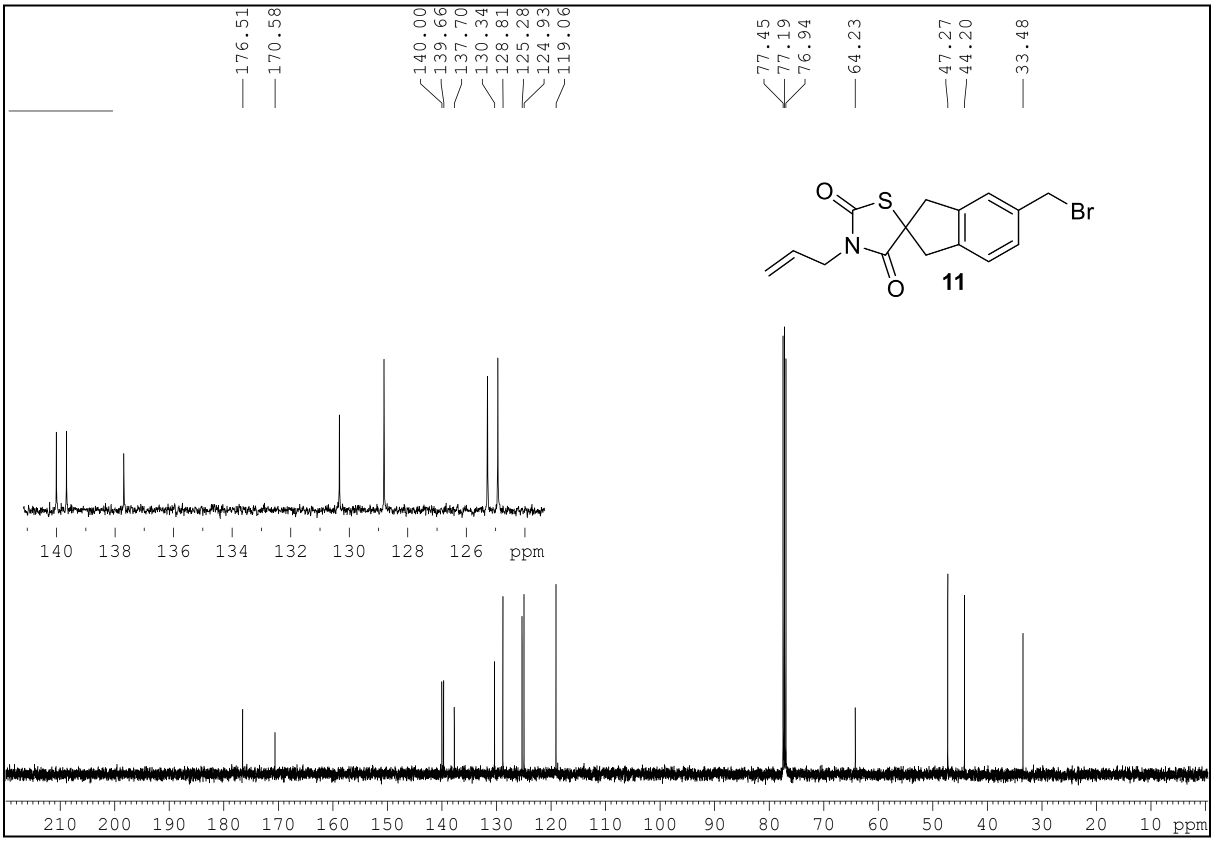

^{1}H NMR (500 MHz, CDCl3) of compound **12**

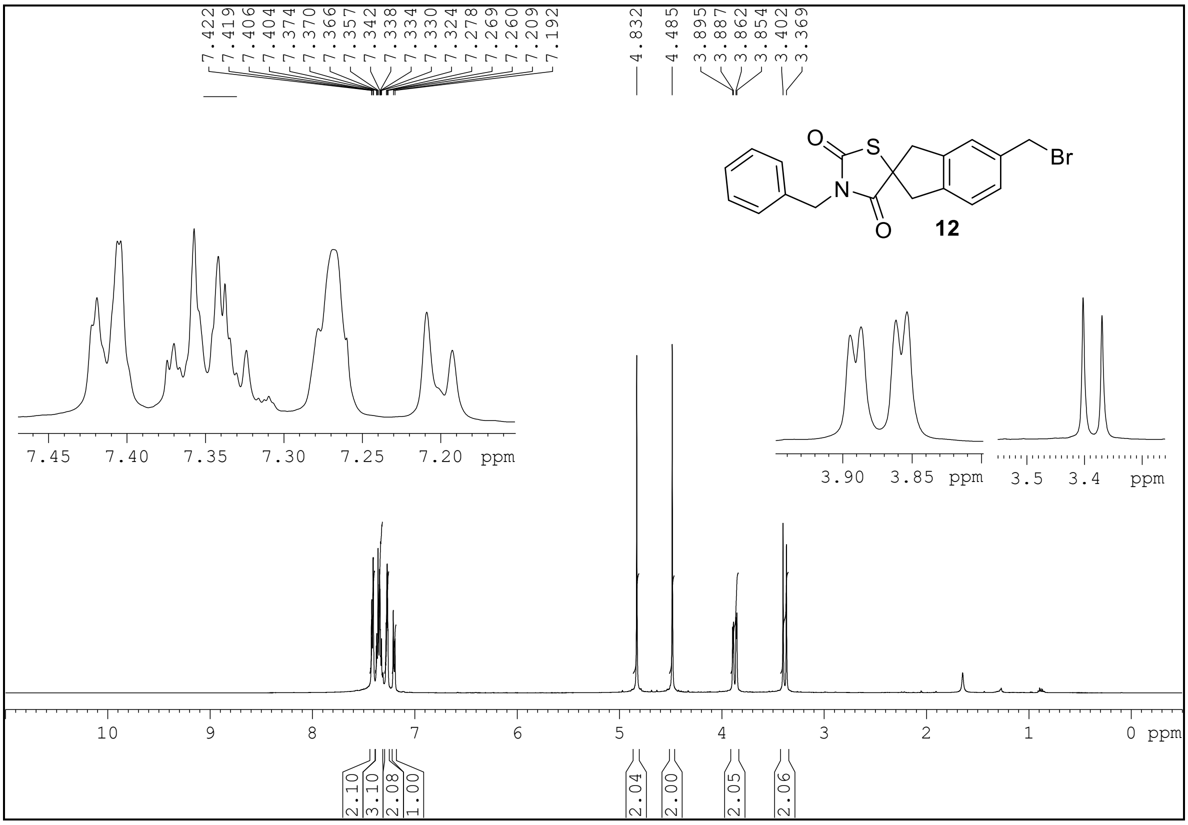

^{13}C NMR (125 MHz, CDCl3) of compound **12**

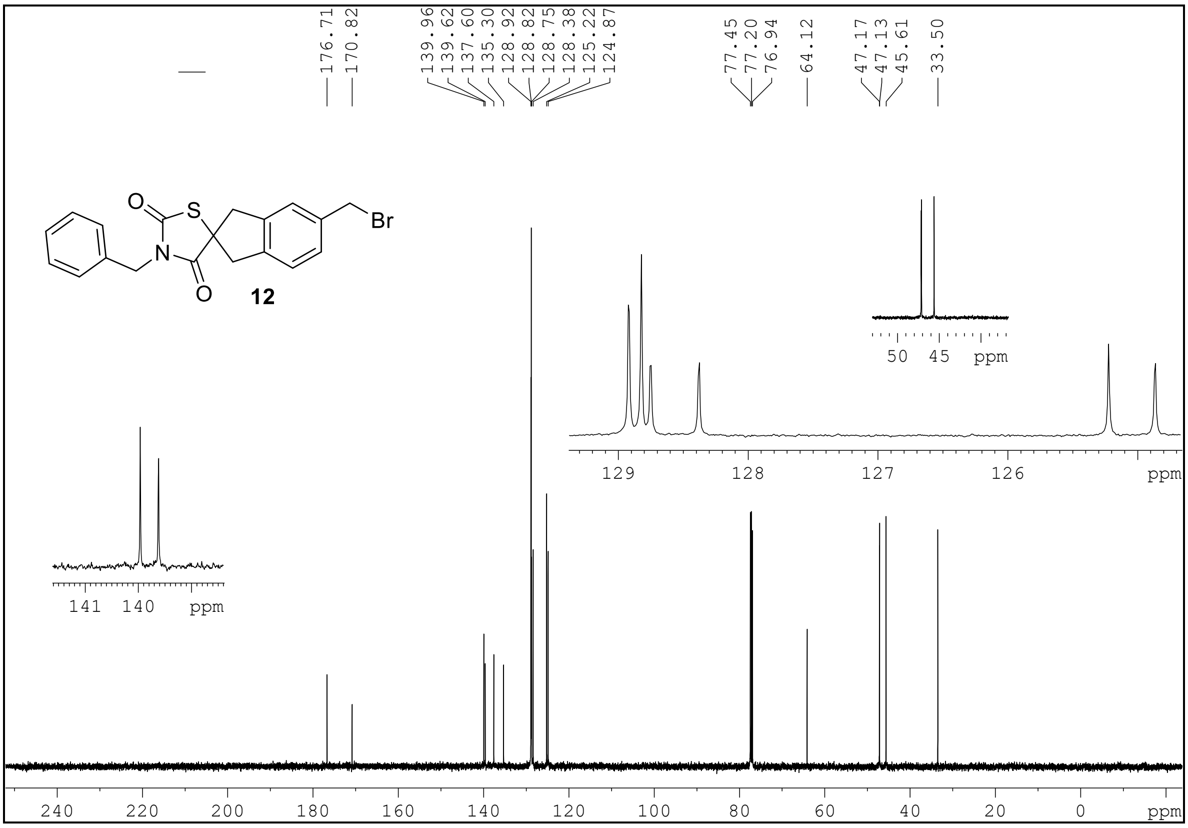

^{1}H NMR (500 MHz, $CDCl_3$) of compound **13**

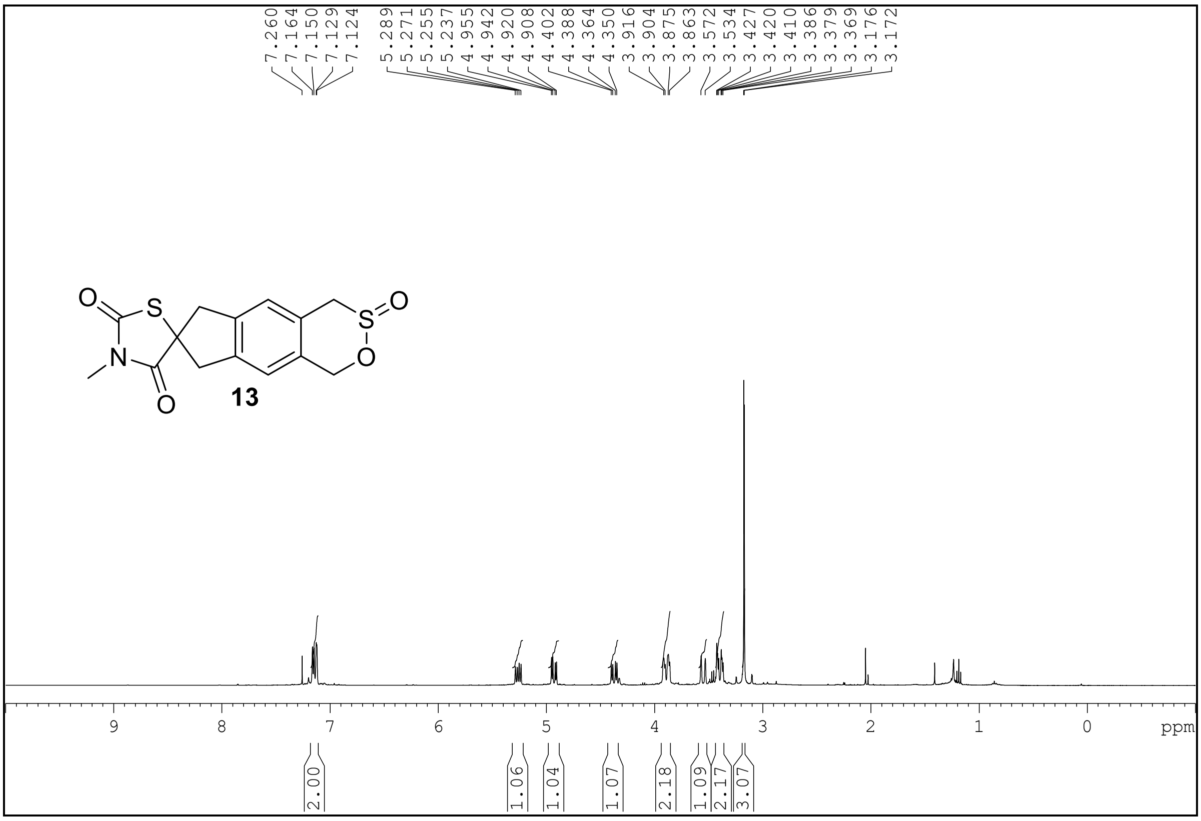

^{13}C NMR (100 MHz, $CDCl_3$) of compound **13**

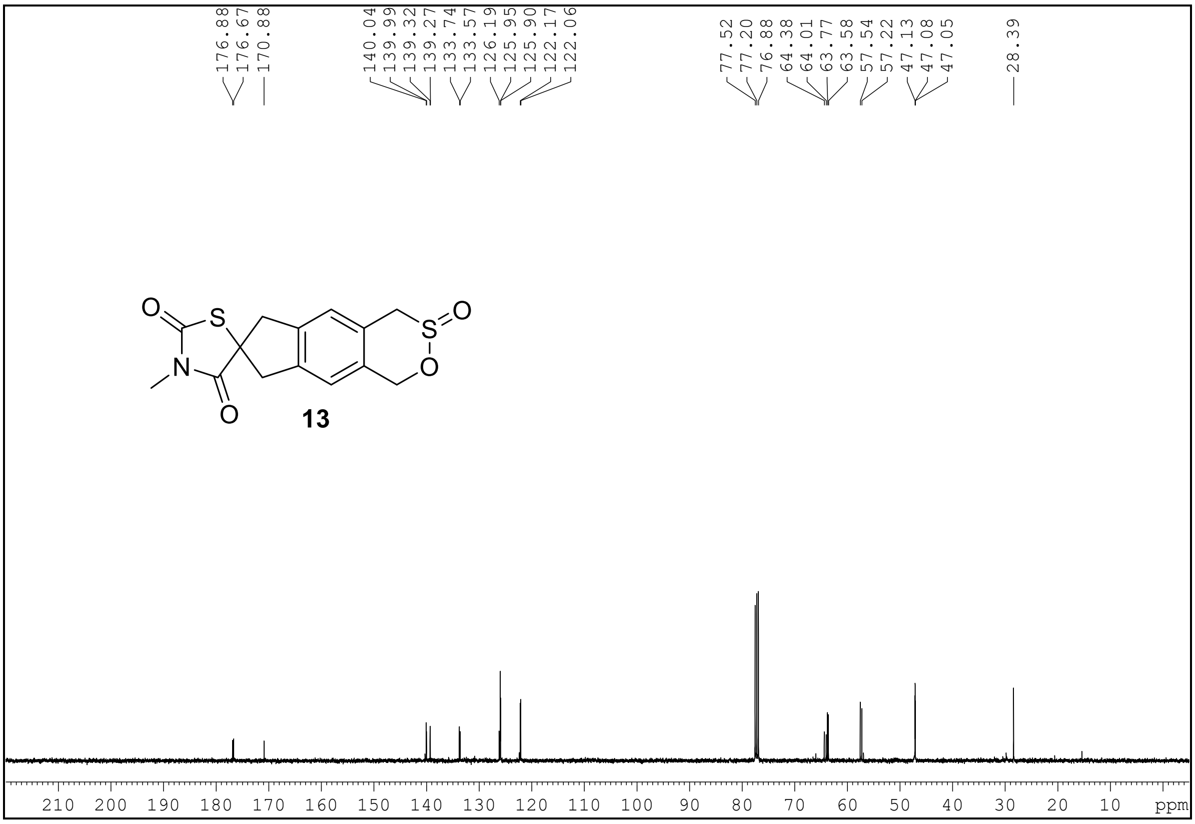

^{1}H NMR (500 MHz, $CDCl_3$) of compound **15a**

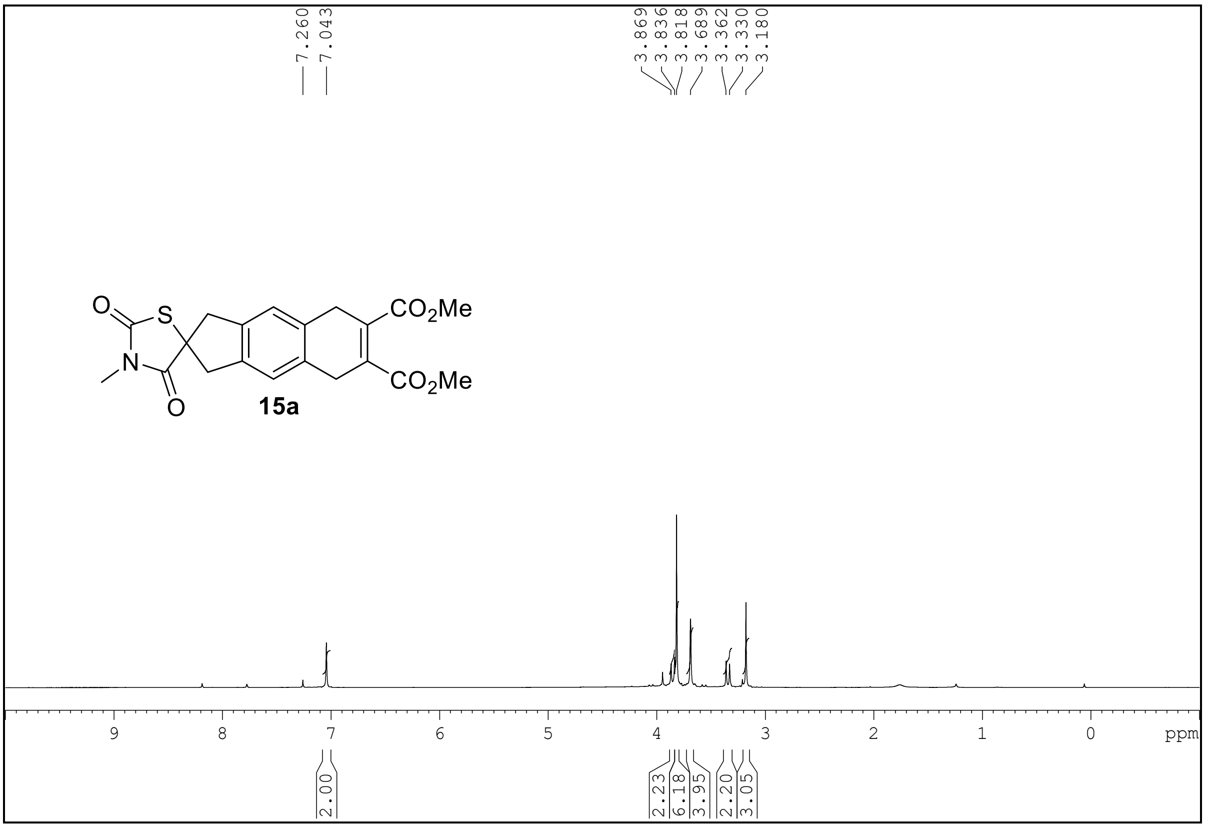

^{13}C NMR (125 MHz, $CDCl_3$) of compound **15a**

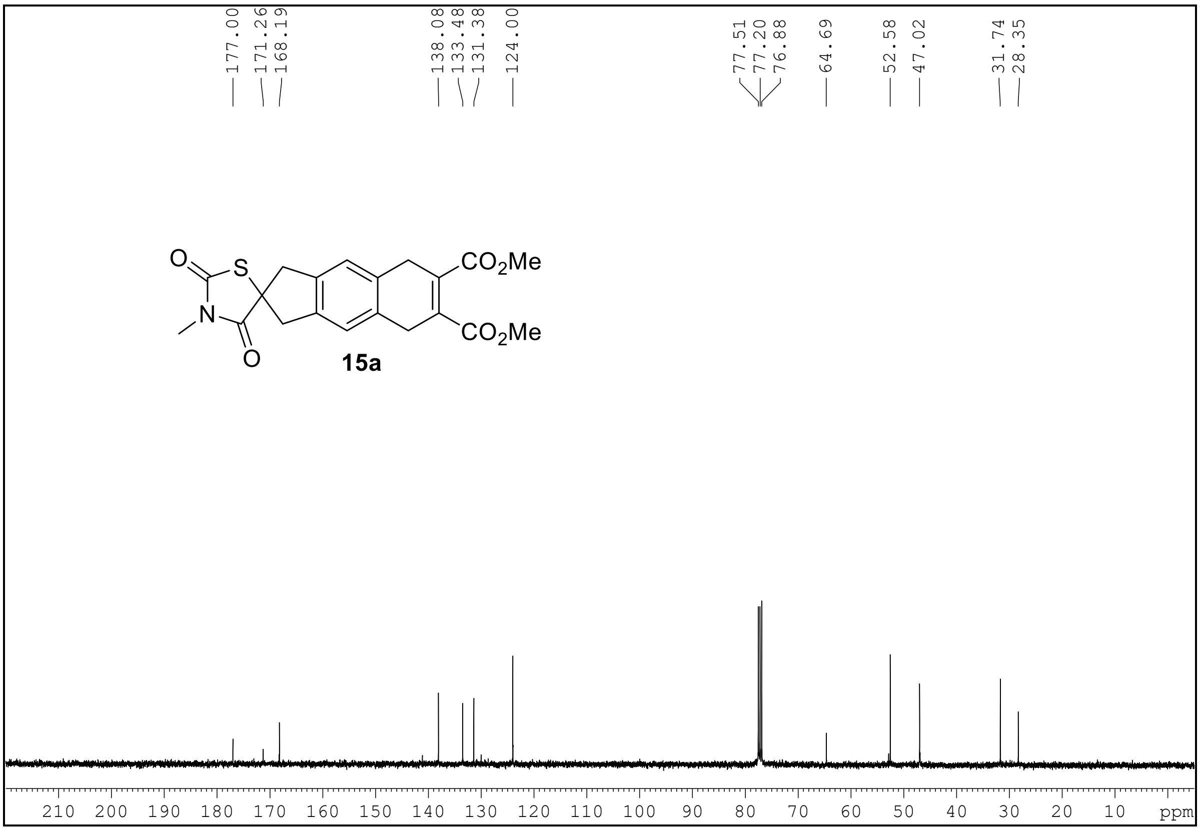

^{1}H NMR (500 MHz, $CDCl_3$) of compound **15b**

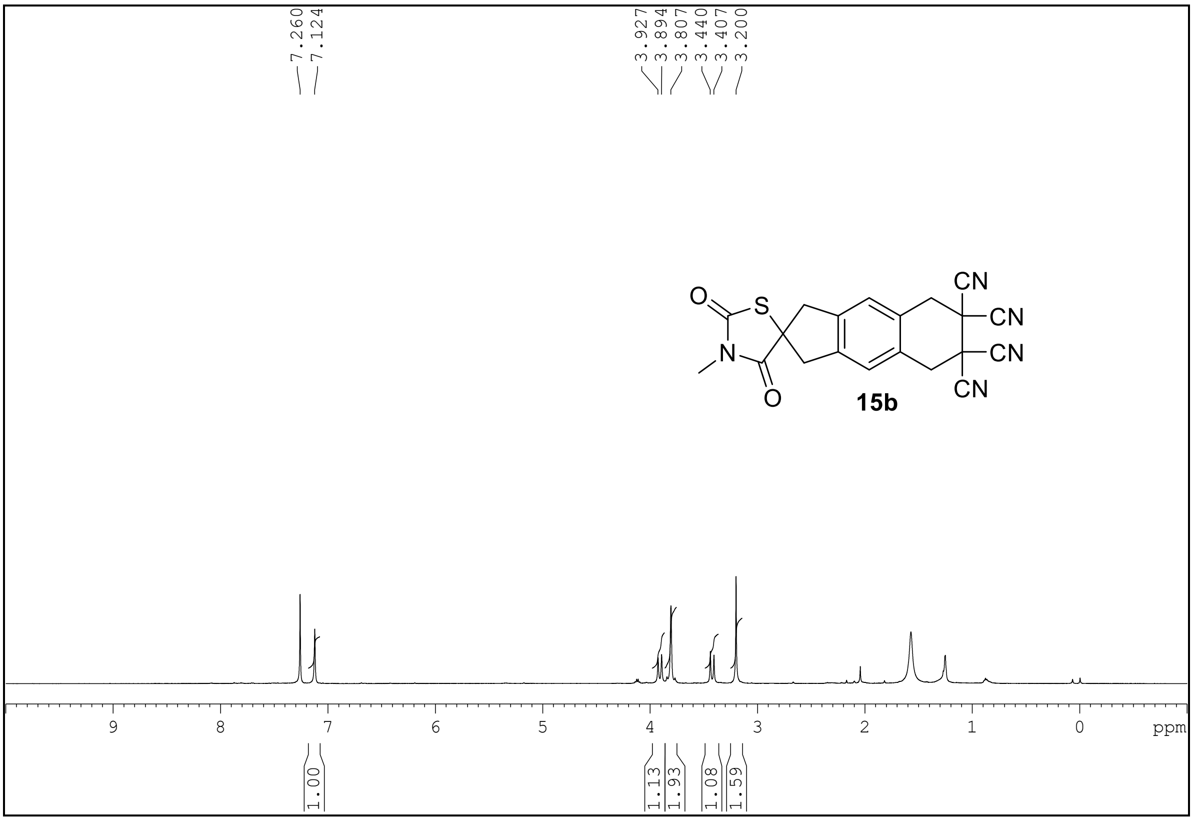

^{1}H NMR (400 MHz, $CDCl_3$) of compound **7f**

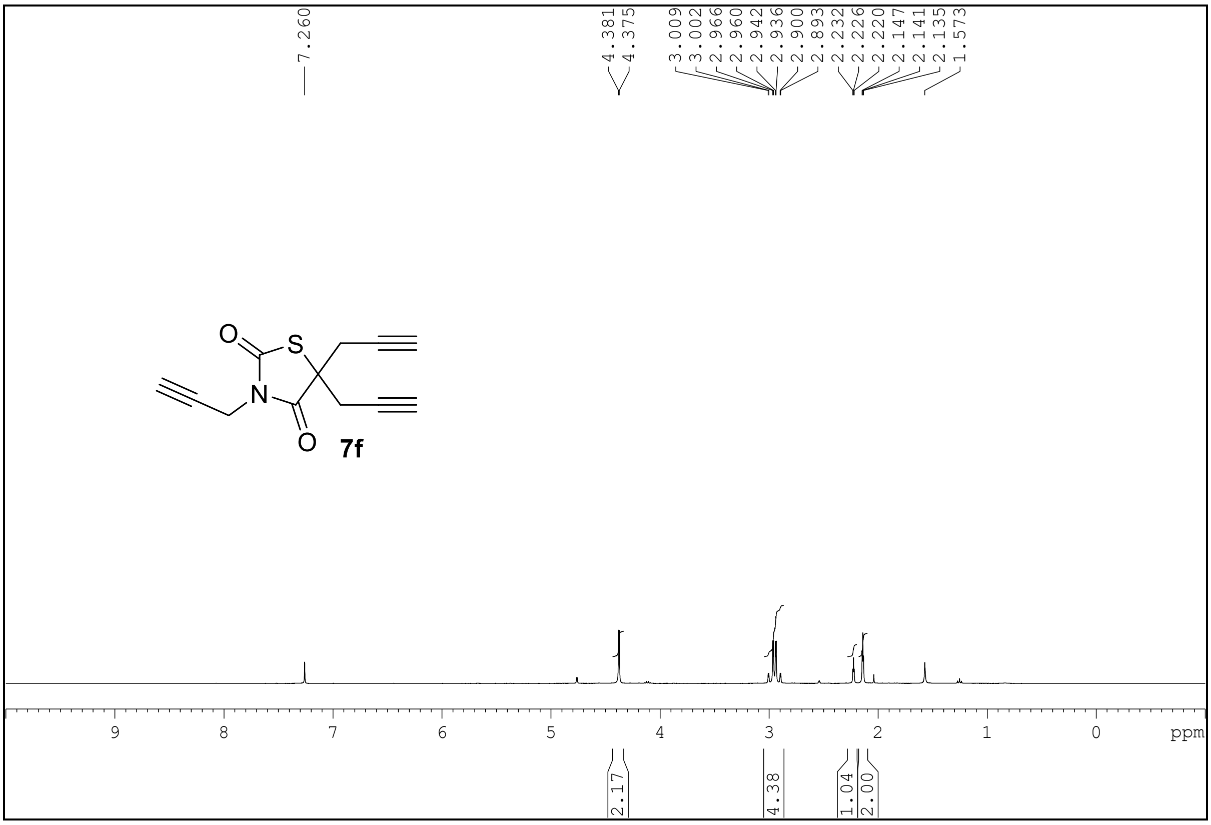

^{1}H NMR (500 MHz, $CDCl_3$) of compound **7e**

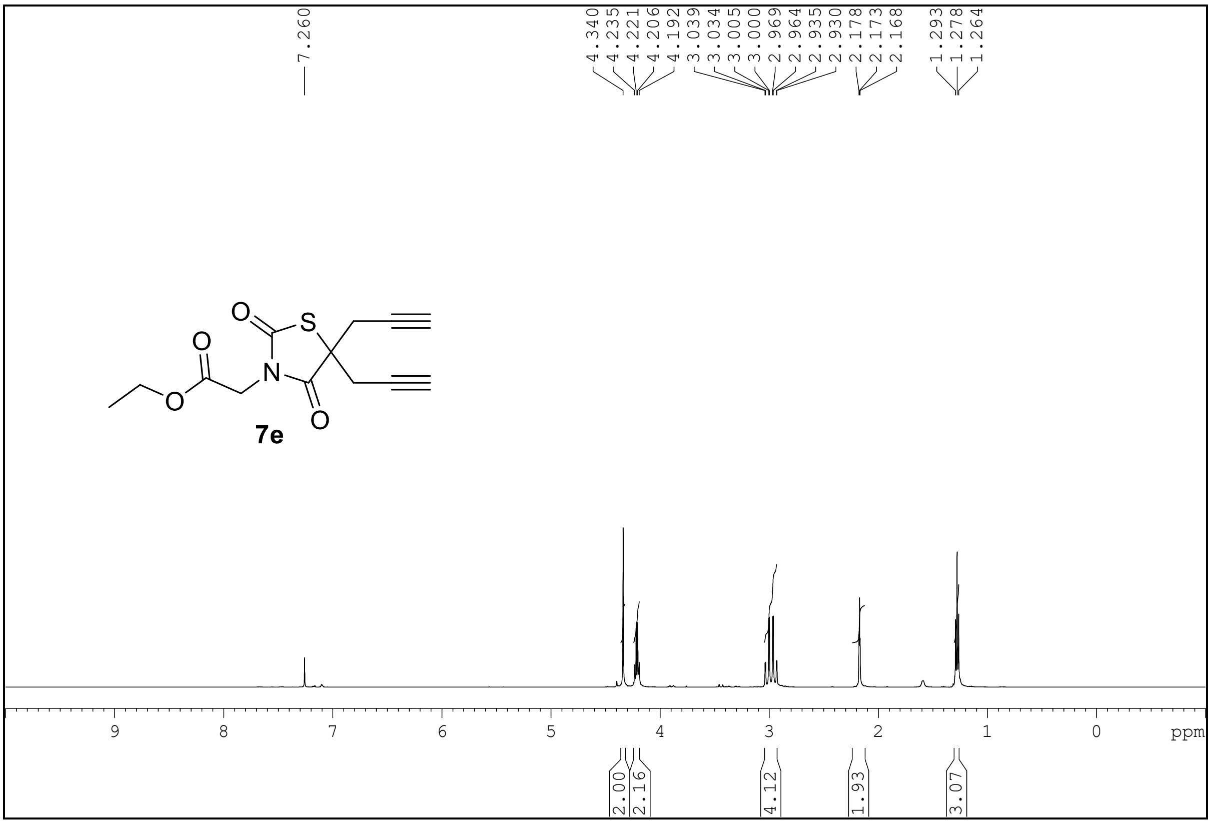

^{13}C NMR (125 MHz, $CDCl_3$) of compound **7e**

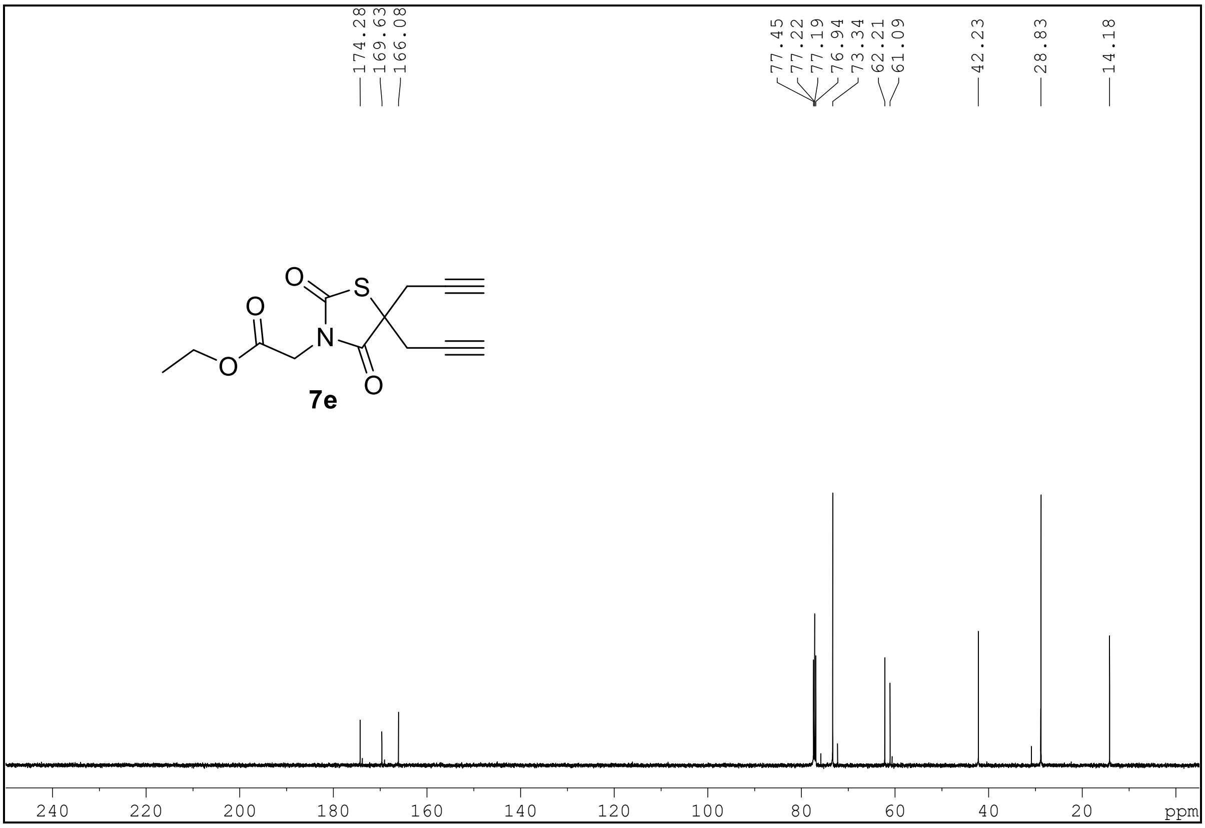

^{1}H NMR (500 MHz, $CDCl_3$) of compound **17**

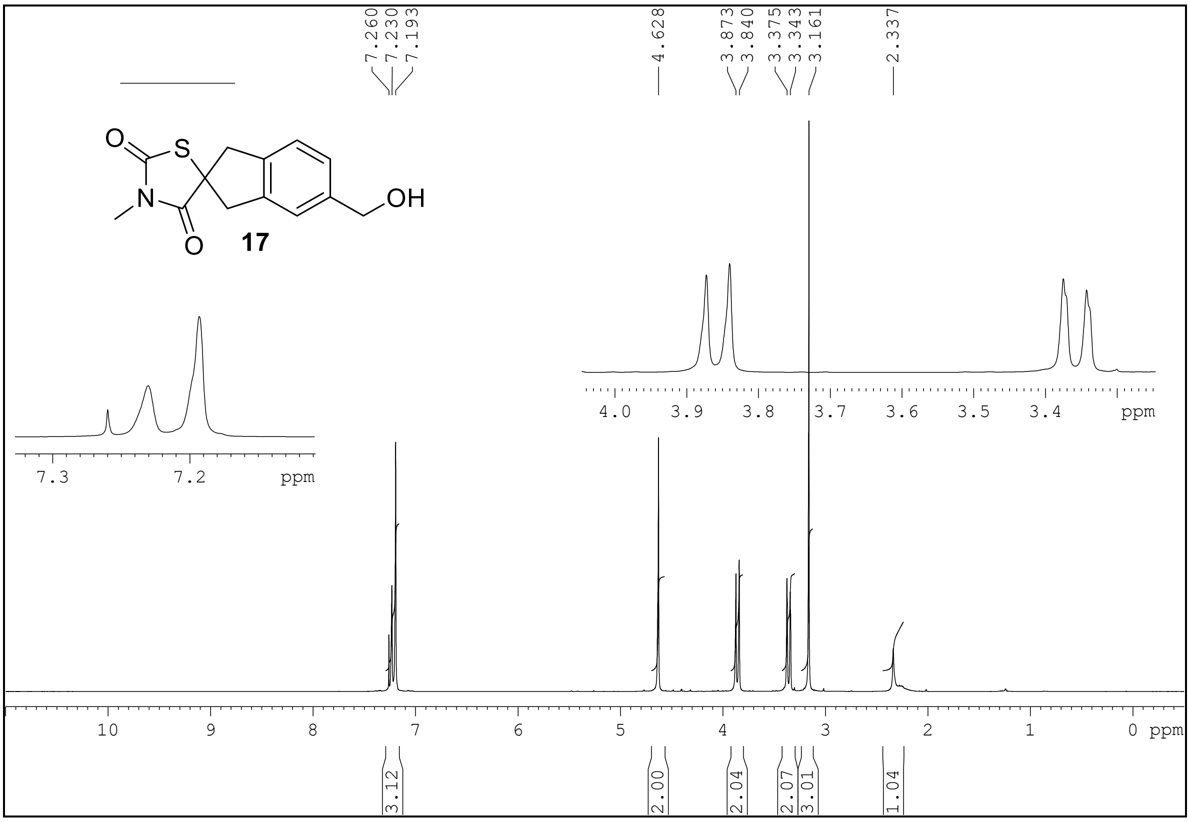

^{13}C NMR (125 MHz, $CDCl_3$) of compound **17**

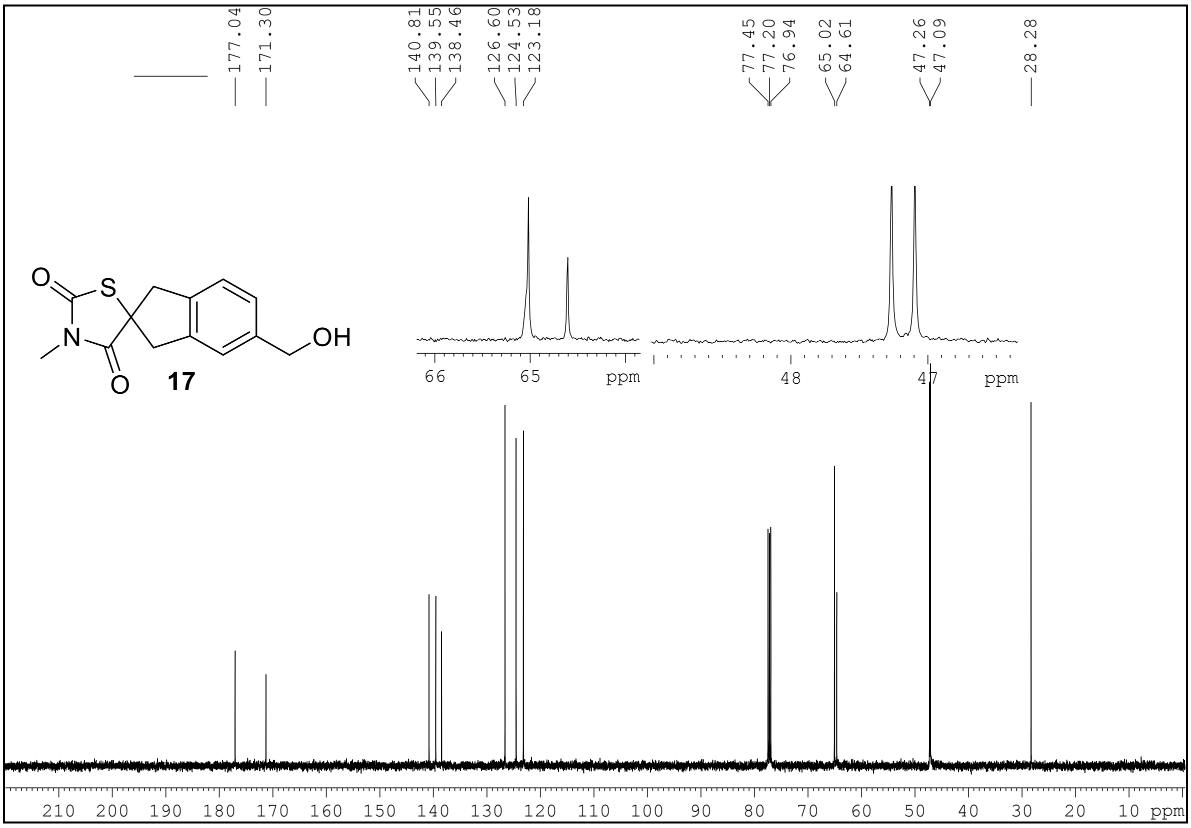

^{1}H NMR (500 MHz, $CDCl_3$) of compound **18**

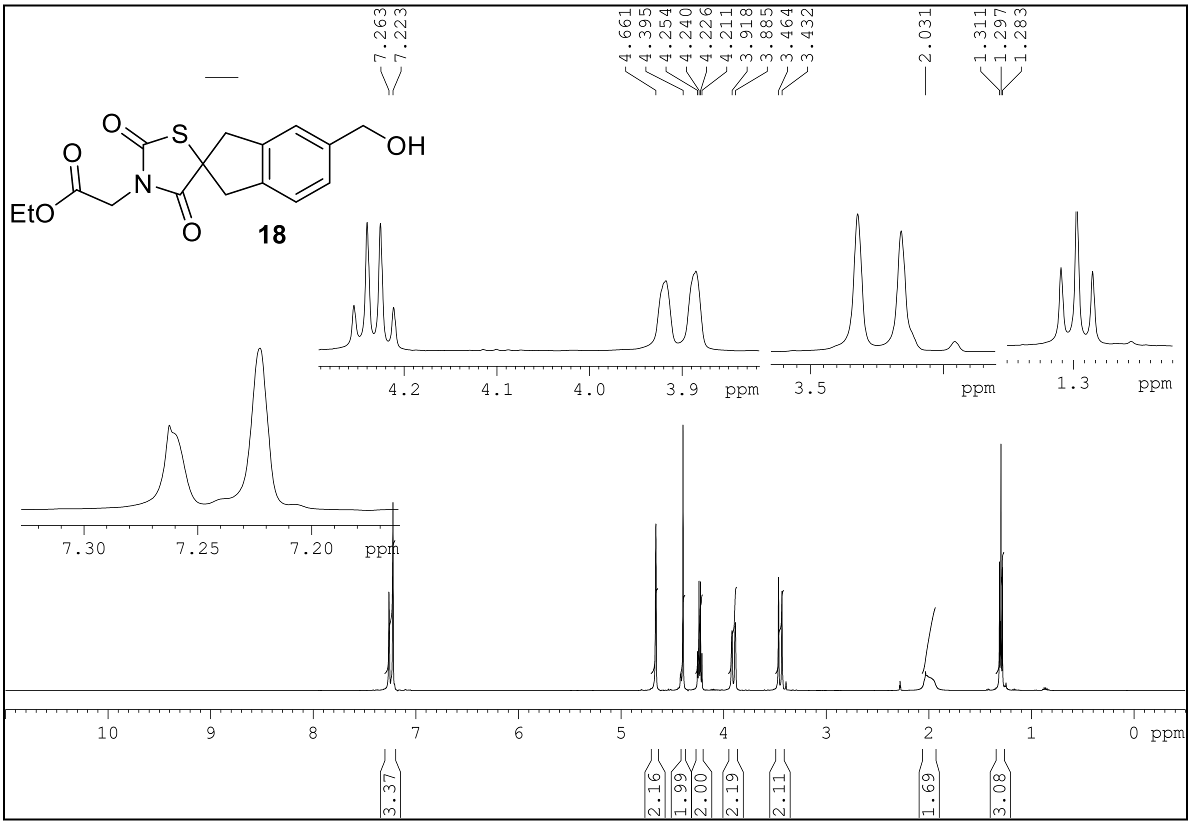

^{13}C NMR (125 MHz, $CDCl_3$) of compound **18**

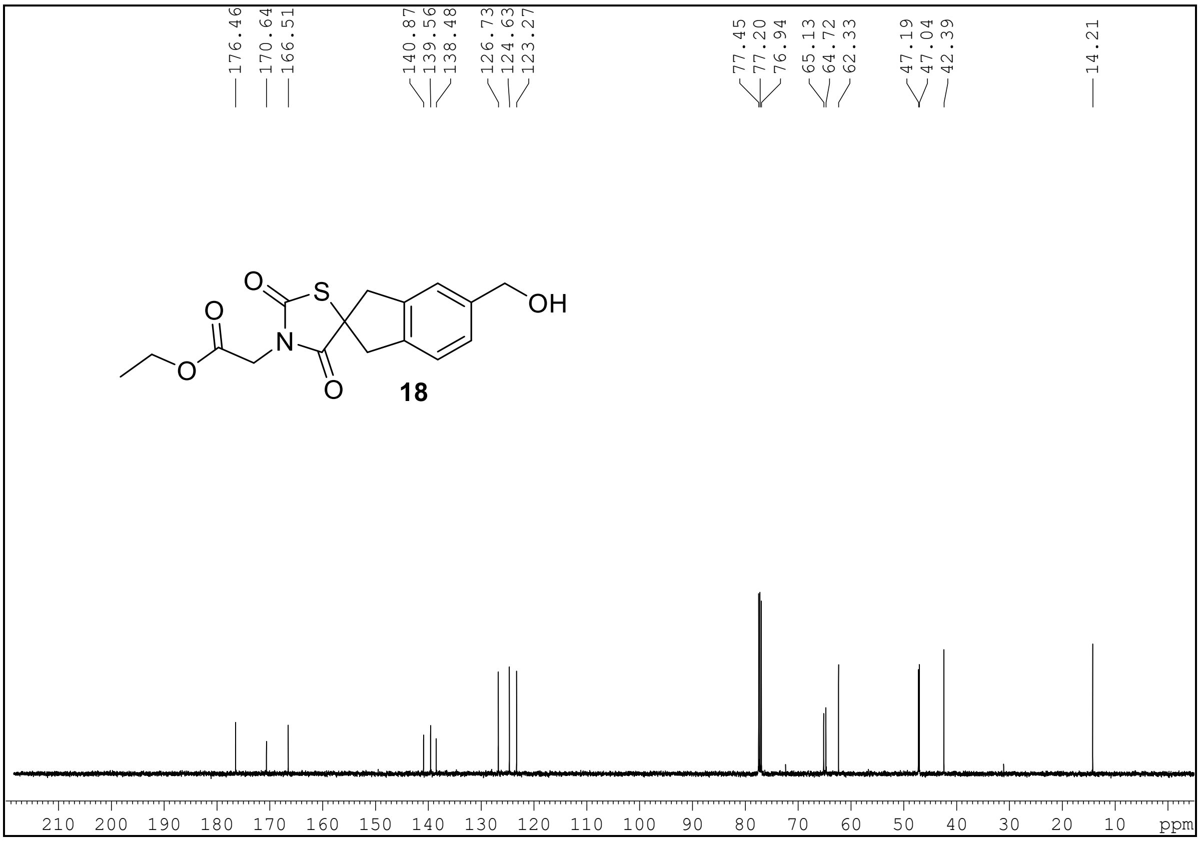

^{1}H NMR (500 MHz, CDCl$_3$) of compound **19**

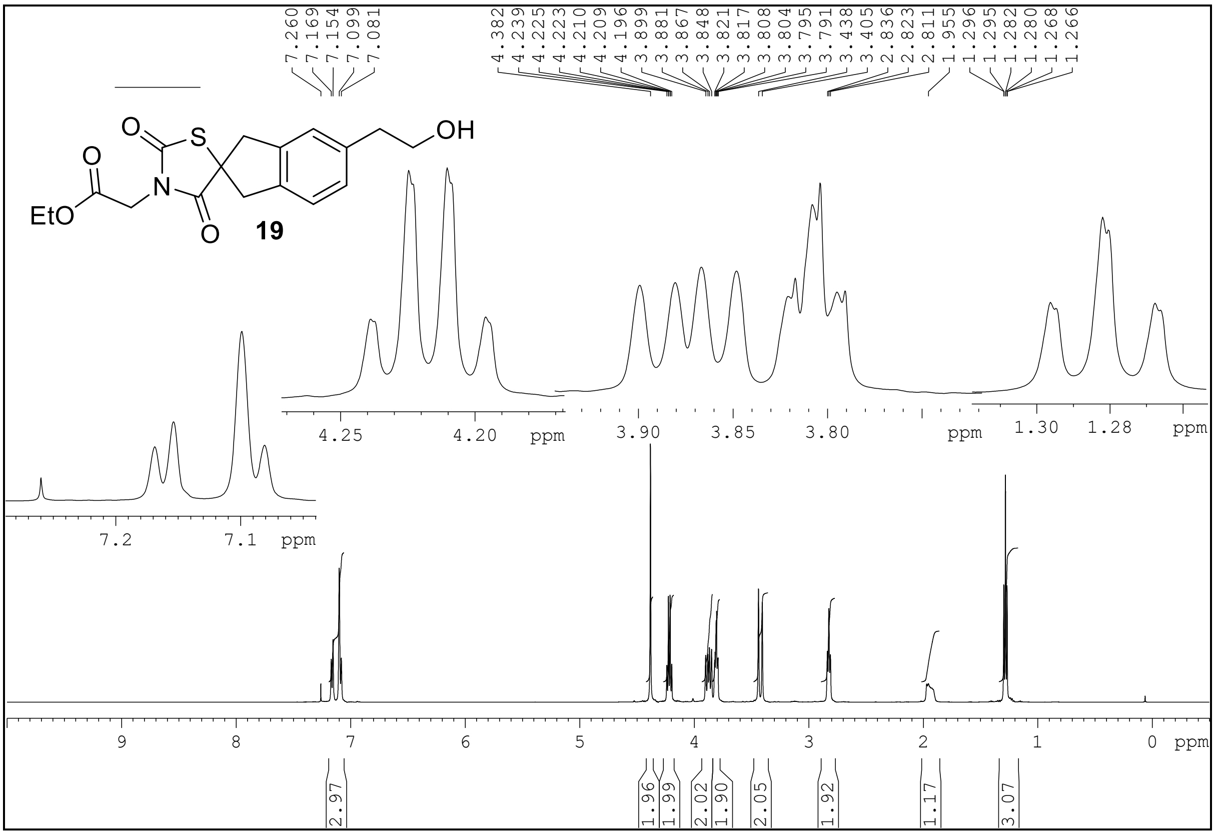

^{13}C NMR (125 MHz, CDCl$_3$) of compound **19**

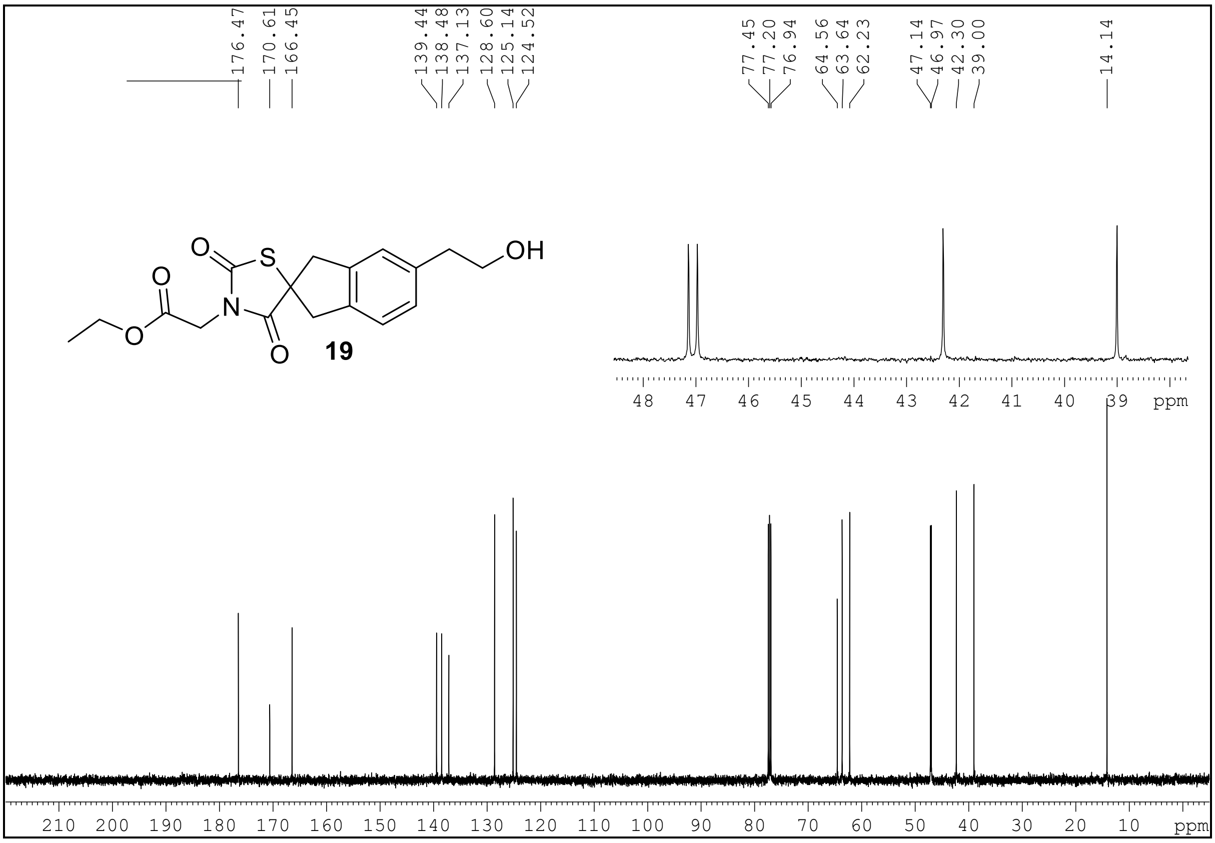

^{1}H NMR (500 MHz, $CDCl_3$) of compound **20**

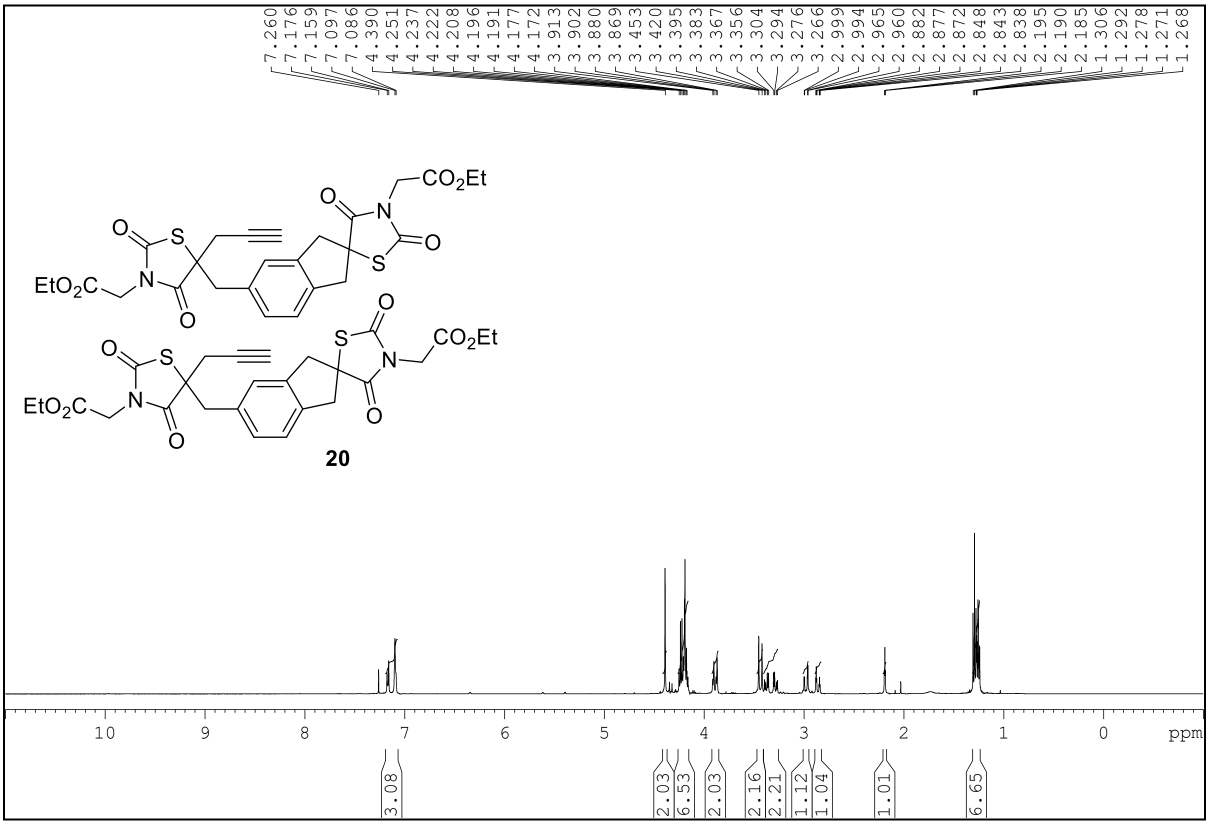

^{13}C NMR (125 MHz, $CDCl_3$) of compound **20**

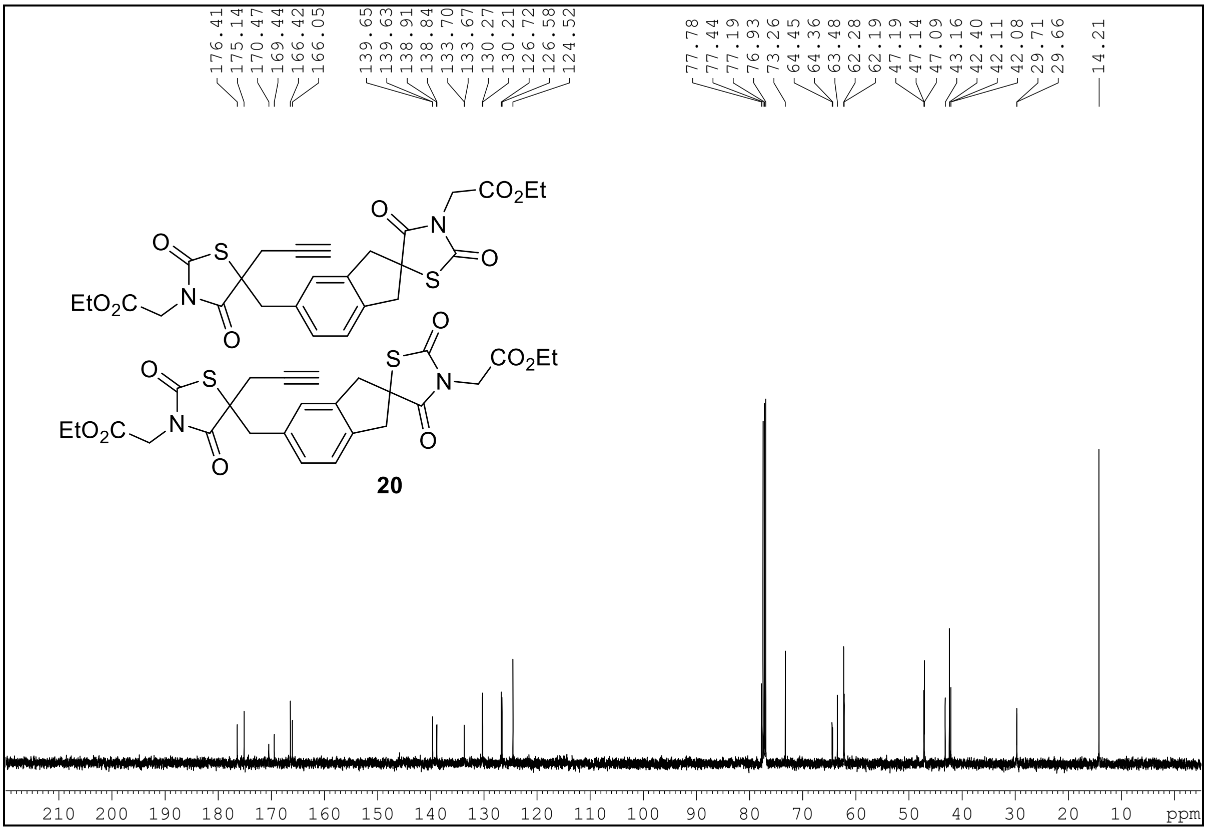

^{1}H NMR (500 MHz, $CDCl_3$) of compound **21**

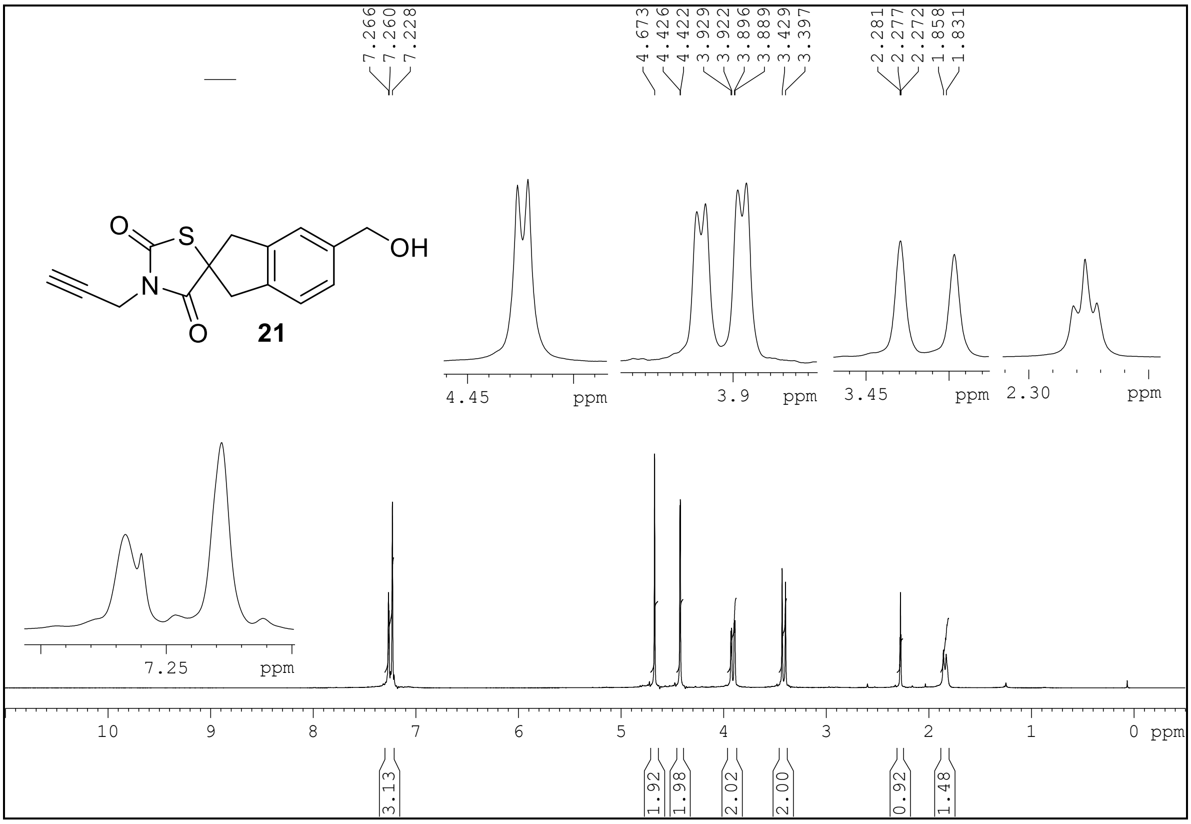

^{13}C NMR (125 MHz, $CDCl_3$) of compound **21**

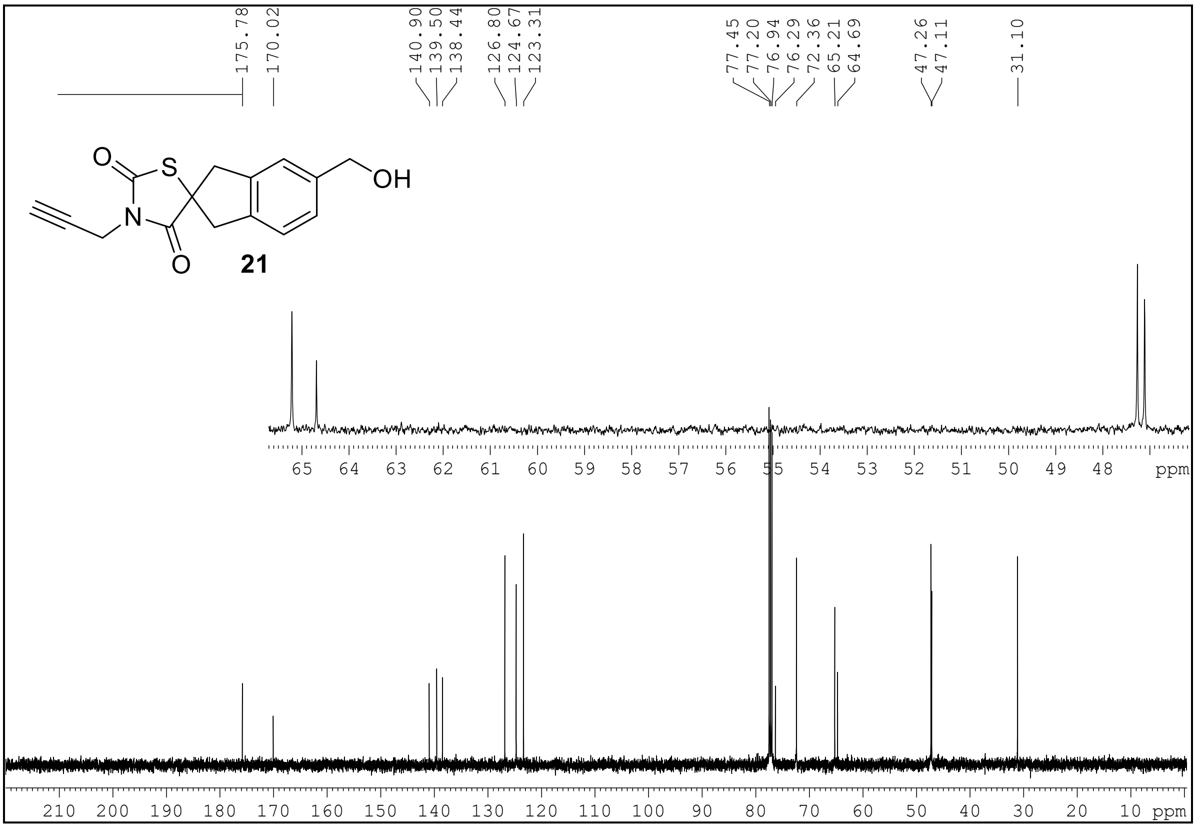

^{1}H NMR (400 MHz, $CDCl_3$) of compound **22**

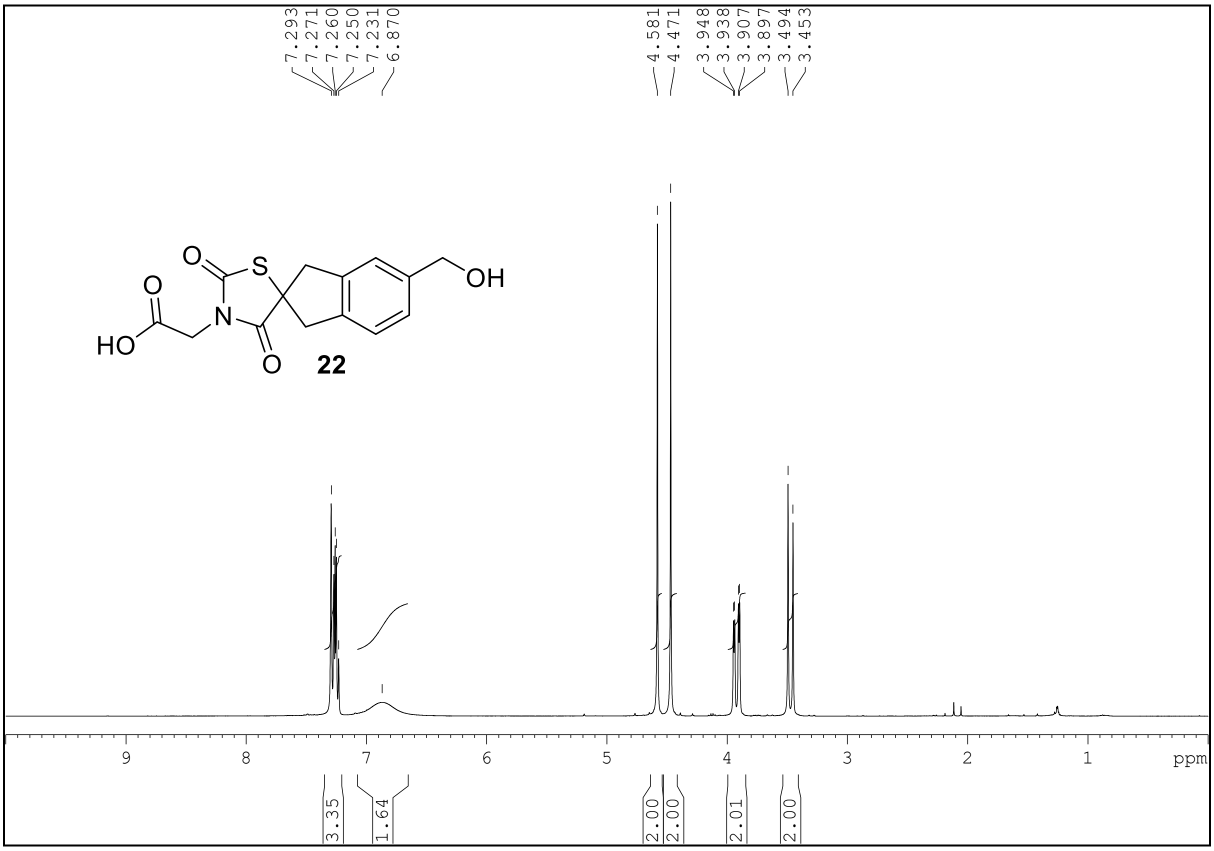

^{13}C NMR (125 MHz, $CDCl_3$) of compound **22**

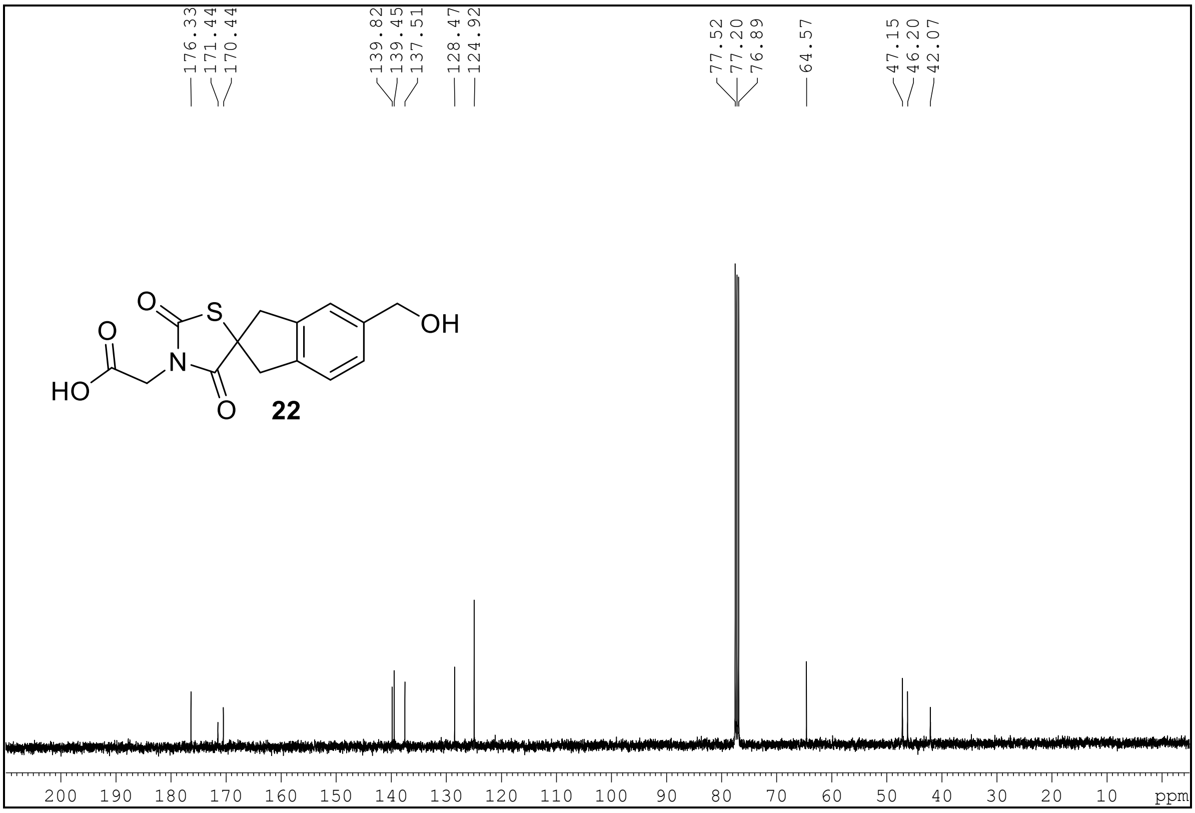

^{1}H NMR (500 MHz, $CDCl_3$) of compound **24**

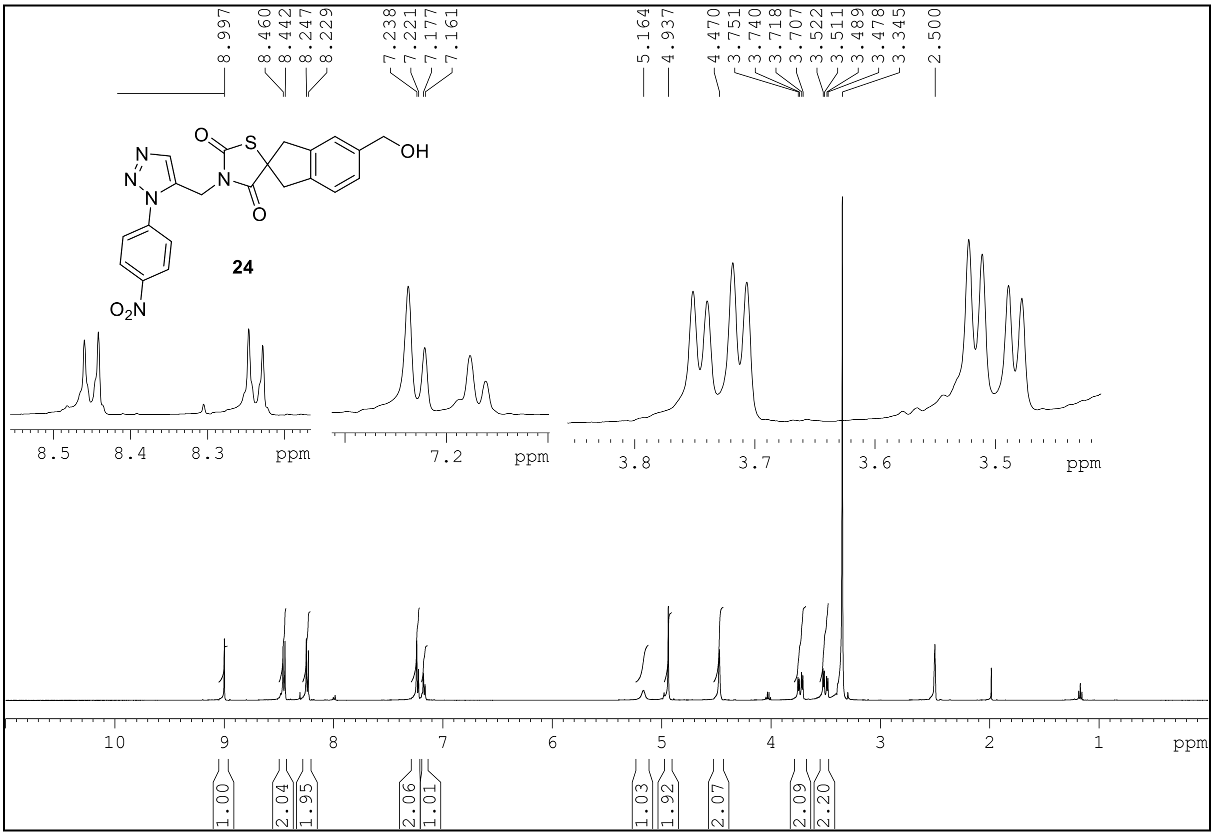

^{13}C NMR (125 MHz, $CDCl_3$) of compound **24**

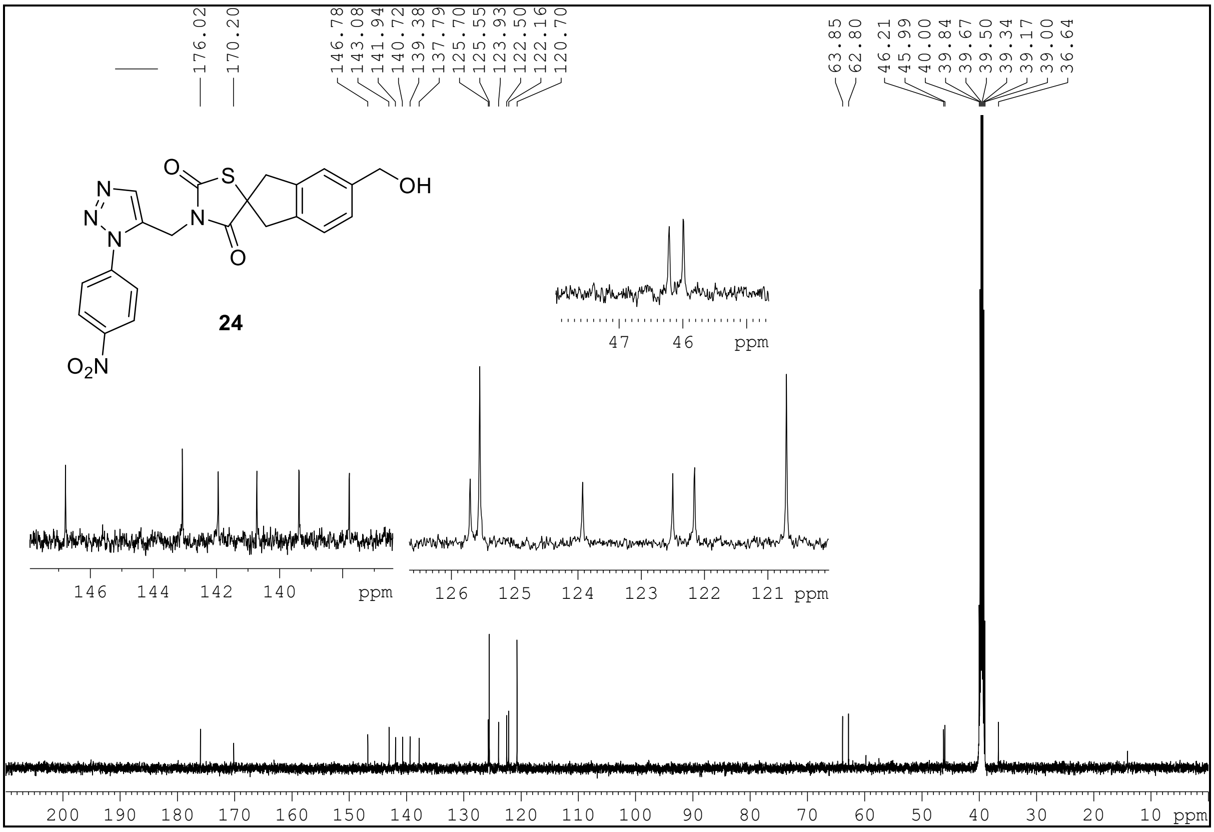

Biology

Cytotoxicity (IC50) and apoptosis

The cytotoxic activity of the 2,4-thiazolidinedione derivatives in comparison with camptothecin and etoposide was studied on Jurkat K562, HEK293, HeLa, and U937 cell lines. To assess the cytotoxicity of the test compounds, cells were incubated for 24 hours with the 2,4-thiazolidinedione derivatives. At the end of the incubation, the cells growing on 24-well plates were treated with trypsin (lines HEK293, HeLa), harvested, washed in culture medium, precipitated, and suspended at a ratio of 1 million in 100 μL. To study the cytotoxicity, a standard MTT test based on the use of (3-(4,5-dimethylthiazol-2-yl)-2,5-diphenyl-2*H*-tetrazolium bromide) was used. The IC50 value was determined from plots of % viability against the dose of the compound added. The percentage of cell growth inhibition was calculated as 100 minus (mean cell culture optical density in the test/mean cell culture optical density in the control) multiplied by 100. The value obtained for the first three wells without addition of the test compound (control triplet), which was measured in parallel for each test compound, was taken to be 100%. The mean value and the error of mean were calculated separately for each concentration of the test compound. The results were used to plot the cell viability (%) versus the test compound concentration, and the dose inhibiting the cell viability by 50% (IC50) and the standard error (SE) of IC50 were calculated using the GraphPad Prism 7.0 software (GraphPad Inc., USA). The data obtained in the experiment are presented in Table S1.

Table S1. Cytotoxic activities in vitro of synthesized spirothiazolidinedione derivatives **17–22** and **24** measured on tumor cell cultures (Jurkat, K562, Hek293, HeLa, U937) (IC50, nM).

Compound	IC_{50} (nM)				
	Jurkat	K562	HEK293	HeLa	U937
Etoposide	0.7±0.009	0.54±0.035	0.99±0.006	0.89±0.033	0.45±0.028
Camptothecin	1.1±0.050	1.2±0.048	0.85±0.067	0.74±0.002	0.54±0.004
**17**	0.57±0.039	0.99±0.084	1.06±0.067	0.99±0.093	0.36±0.013
**18**	0.99±0.006	1.34±0.04	1.43±0.06	0.96±0.056	1.07±0.028
**19**	0.97±0.018	1.15±0.076	1.29±0.045	0.89±0.034	1.17±0.087
**20**	0.47±0.051	0.26±0.034	0.49±0.056	0.54±0.076	0.29±0.065
**21**	0.87±0.045	1.29±0.074	1.08±0.023	1.43±0.032	0.87±0.016
**22**	0.34±0.078	0.57±0.006	0.68±0.011	0.65±0.034	1.17±0.087
**24**	1.11±0.018	1.09±0.091	1.11±0.073	1.01±0.089	1.55±0.019

The highest activity against cells of chronic myeloid leukemia (line K562) was observed with compound **20** (IC50 = 0.26 nM). The effect of compound **20** is practically comparable to the action of etoposide (IC50 = 0.54 nM) on this cell line. The lowest activity with respect to all

studied cell lines was shown by the derivatives of 2,4-thiazolidinedione **18**, **19**, **21** and **24** (IC50 cm in Table S1). Compounds **20** and **17** showed the highest activity (IC50 = 0.29 and 0.36 nM, respectively) for leukemic monocytic lymphoma cells (U937), and **22** was active against T-cell leukemia (Jurkat) (IC50 = 0.34 nM). Based on the obtained data, at the initial stages of the study of the ability of drugs to stimulate cell death, it was decided to use three compounds (**17**, **20** and **22**) to study apoptosis and the cell cycle in cells of the corresponding cell cultures.

Thus, we conducted an investigation of apoptosis and the effect on the cell cycle by above mentioned 2,4-thiazolidinedione derivatives. For the study of apoptosis and the cell cycle, the lines Jurkat, K562 and U937 were chosen.

Thus, the highest percentage of apoptosis when compound **22** was added to the culture of Jurkat cells was observed at a concentration of 0.75 nM and was 68.65% (Figure S1). A similar picture is observed for compounds **17** and **20**. The percentage of late apoptosis was about 95% in K562 cells (compound **20**) (Figure S2) and 30.28% for line U937 (compound **17**) (Figure S3).

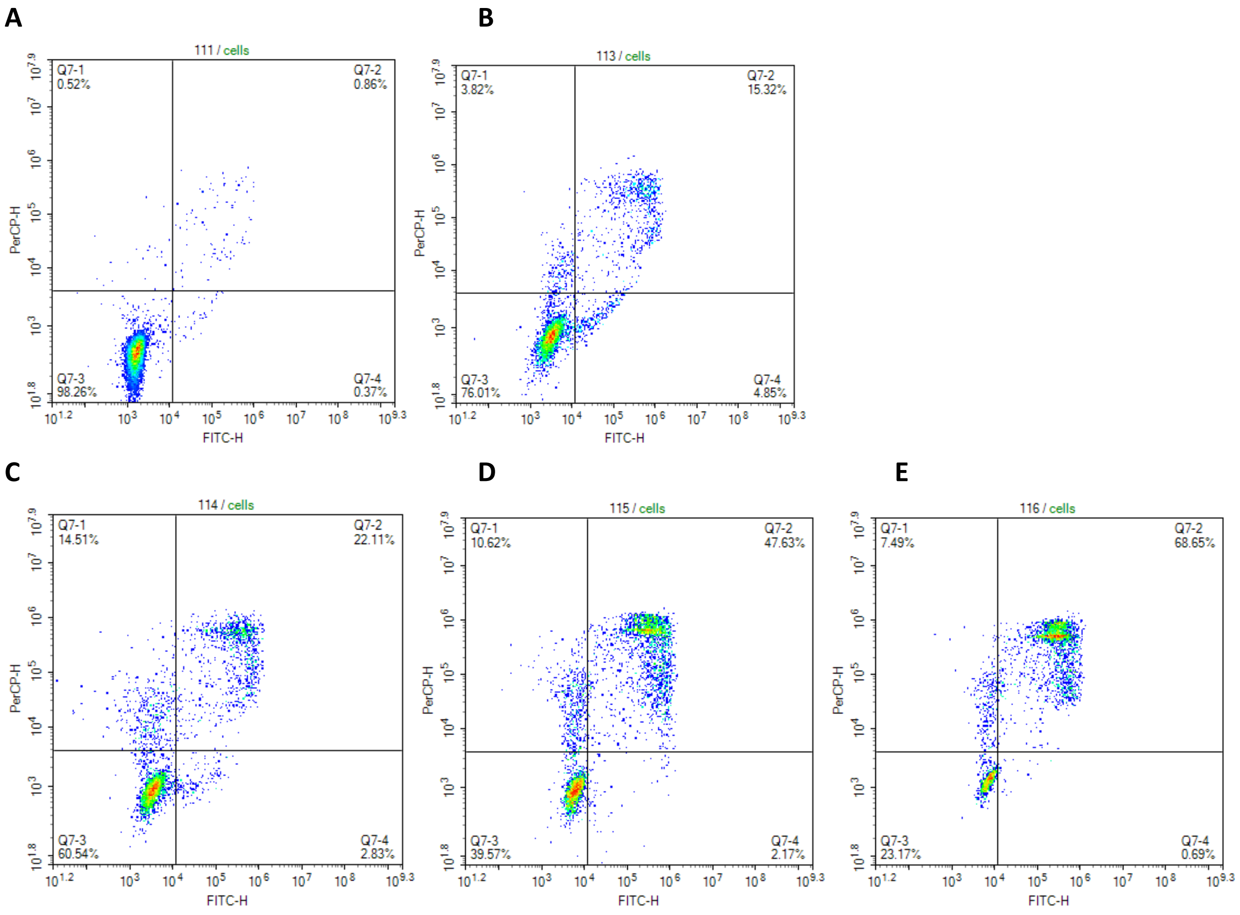

Figure S1. Tumor cell line Jurkat, treated with different concentrations of compound **22** and stained with annexineV / 7-AAD and analyzed using flow cytometry. (**A**) Control; (**B**) **22** (0.1 nM); (**C**) **22** (0.25 nM); (**D**) **22** (0.5 nM), (**E**) **22** (0.75 nM)

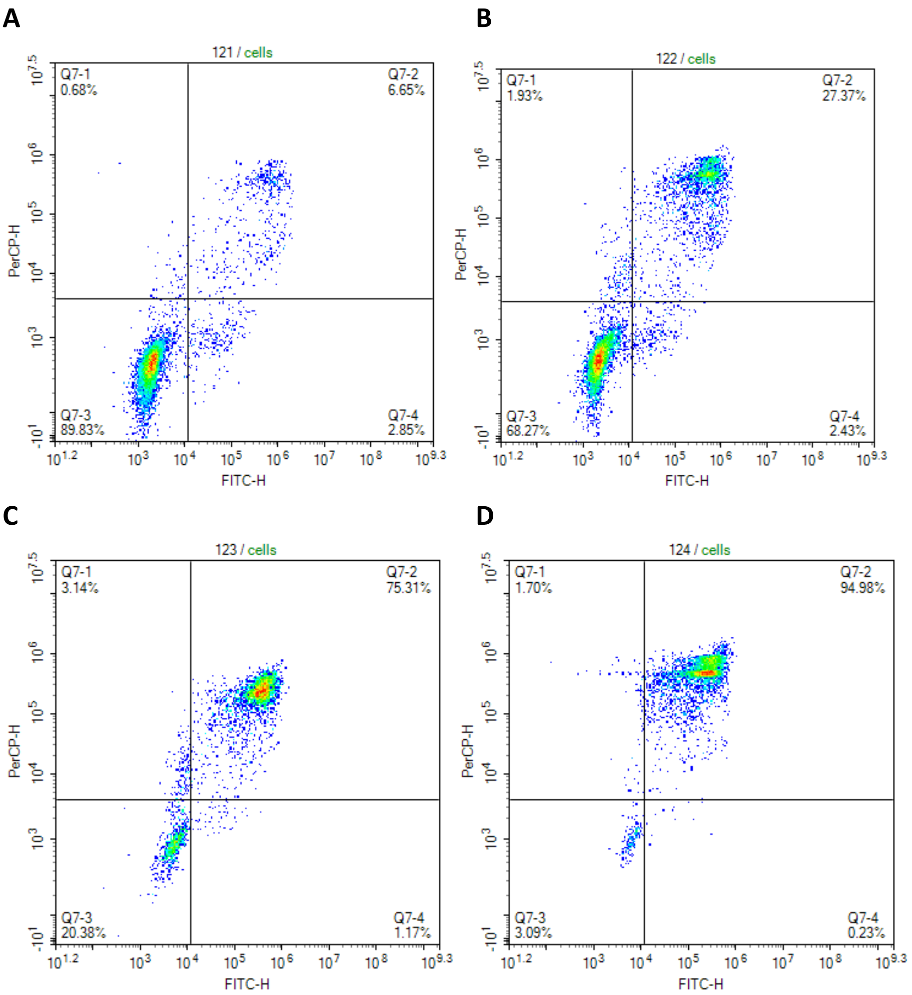

Figure S2. Tumor cell line K562, treated with different concentrations of compound **20** and stained with annexineV / 7-AAD and analyzed using flow cytometry. (A) **20** (0,1 nM) (B) **20** (0,25 nM); (C) **20** (0.5 nM); (D) **20** (0,75 nM).

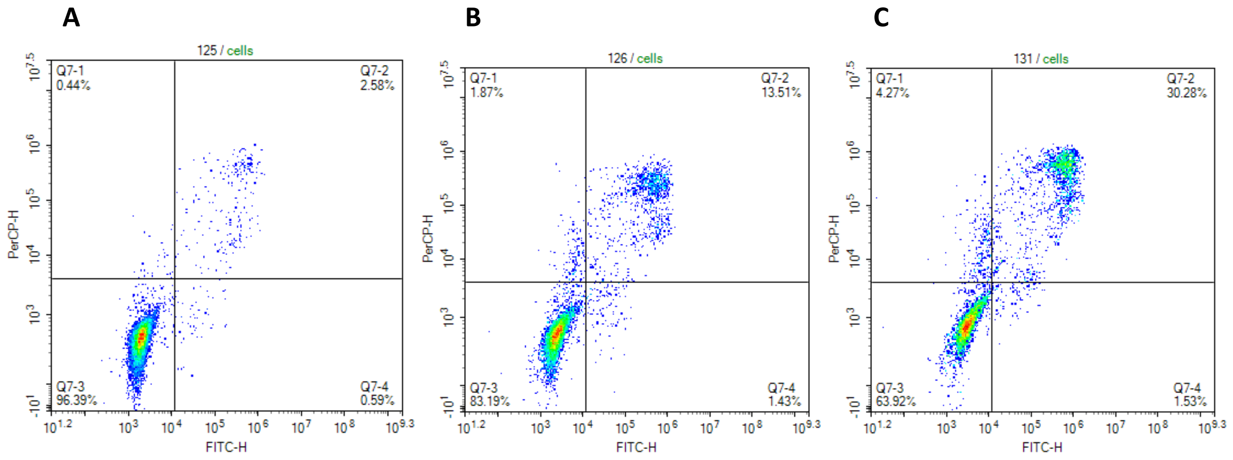

Figure S3. Tumor cell line U937, treated with different concentrations of compound **17** and stained with annexineV / 7-AAD and analyzed using flow cytometry. (A) **17** (0.1 nM) (B) **17** (0.2 nM); (C) **17** (0.4 nM),

Cell cycle analysis

Jurkat K562 and U937 cells at a density of 1 × 10^6 cells/mL were treated with different concentrations of compounds **17**, **20** or **22** for the indicated times. To investigate the irreversibility of the cell cycle unit, cells after incubation with the test substances were cultivated in fresh medium for additional 24–48 hours - a so-called "wash" was carried out. After treatment, cells were collected by trypsinization and centrifugation, washed with PBS, and fixed with ice-cold 70% ethanol at −20 °C overnight. Fixed cells were resuspended in 0.5 mL of PBS containing 50 μL of RNase (Sigma; 1 mg mL^{-1} in PBS) and 50 μL of propidium iodide (PI) (Sigma, 500 mg mL^{-1} in PBS) for 30 min at 37 °C in the dark. The cell cycle distribution was analyzed using the NovoCyte Flow Cytometry system (ACEA Biosciences, Inc., USA). The data were acquired and analyzed using the ACEA NovoExpress® Software.

Oncogenic transformation of cells is accompanied by disruption of the program of strict control over the integrity of the genome, which, in particular, manifests itself in the loss of the ability of transformed cells to block proliferation after DNA damage. Cells unable to stop the cell cycle for damage repair either start the apoptosis program, or proliferate with genetic defects, increasing the genetic heterogeneity of the population. From numerous studies, it is known that DNA damage in cells leads to the arrest of the cell cycle at control or checkpoints. In particular, the cytotoxic quinoline alkaloid camptothecin, a known DNA-damaging agent due to irreversible inhibition of topoisomerase, blocks the cell cycle in many tumor lines. The results of flow cytometry showed that in all three lines, Jurkat, K562 and U937, a hypodiploidal DNA peak appears after 24 hours after the action of compounds **17**, **20** and **22**, which shows the inability to stop the fission cycle at the checkpoints, and, ultimately, leads to cell death.

A **B** **C**

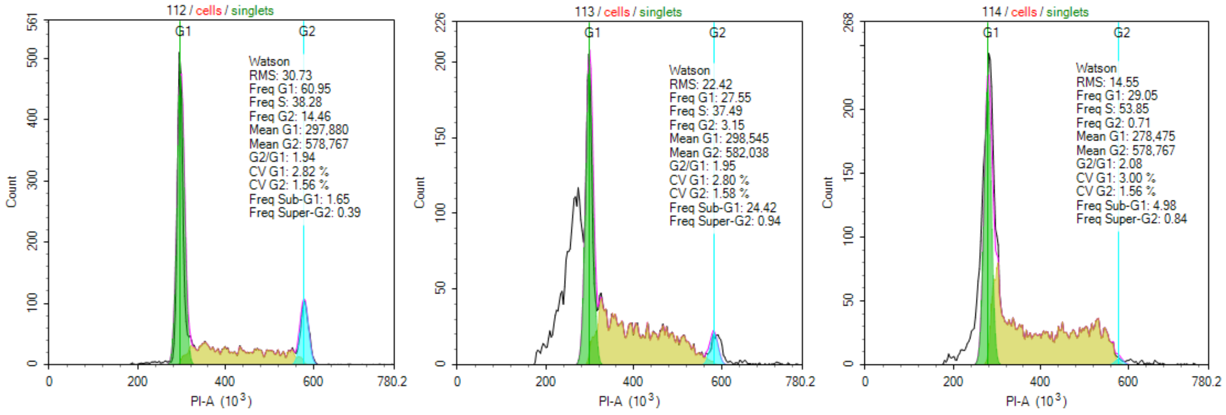

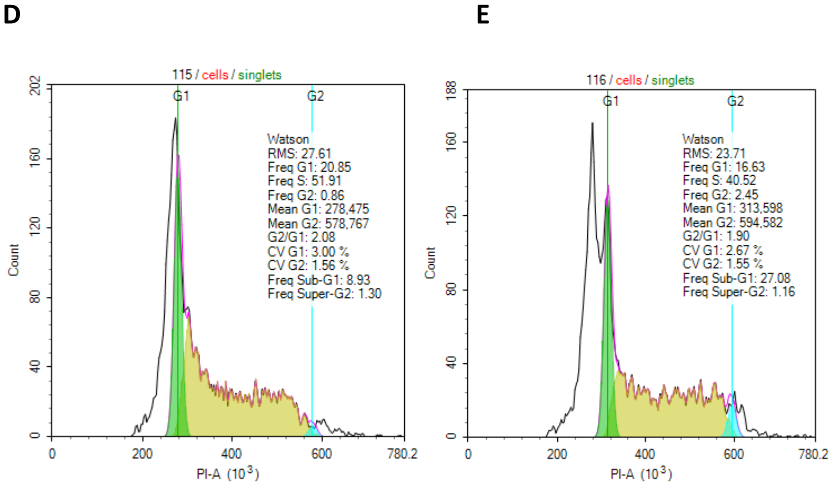

Figure S4. Cell cycle phases on Jurkat cells treated with compound **22**. Upper row: A - control, B - 0.1 nM, C - 0.25 nM, D - 0.5 nM, E - 0.75 nM. The time of incubation of substance **22** with cells is 24 hours.

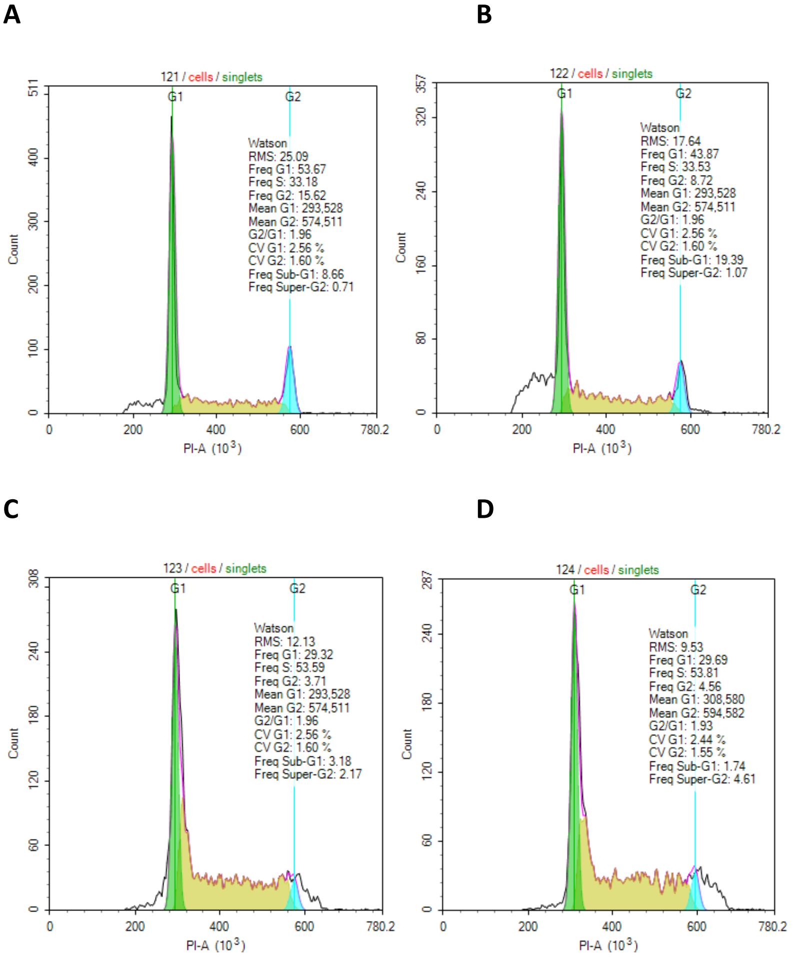

Figure S5. Cell cycle phases on K562 cells treated with compound **20**. Upper row: A - 0.1 nM, B - 0.25 nM, C - 0.5 nM, B - 0.75 nM. The incubation time of substance **20** with cells is 24 hours.

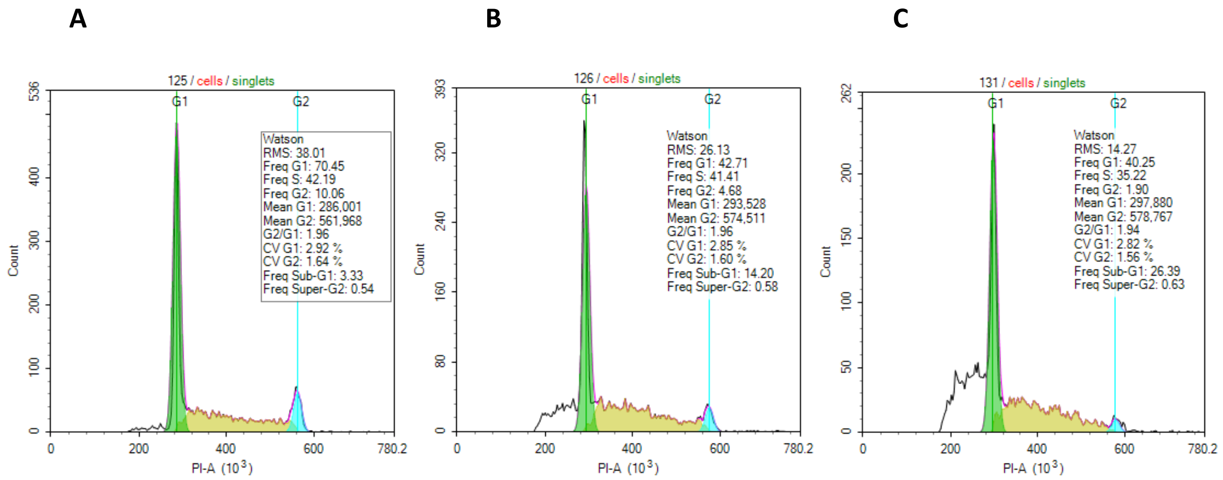

Figure S6. Cell cycle phases on U397 cells treated with compound **17**. A - 0.1 nM, B - 0.2 nM, C - 0.4 nM. The incubation time of substance **17** with cells is 24 hours.

References

1. Dhara, K.; Paladhi, S.; Midya, G. C.; Dash, J. *Org. Biomol. Chem.* **2011**, *9*, 3801–3807. doi: 10.1039/C0OB01248C.

2. Lima, A. B.; Behnam, M. A. M.; Sherif, Y. E.; Nitsche, C.; Vechi, S. M.; Klein, C. D. *Bioorg. Med. Chem.* **2015**, *23*, 5748–5755. doi: 10.1016/j.bmc.2015.07.012.
3. Kotha, S.; Sreevani, G. *Eur. J. Org. Chem.* **2018**, 5935–5941. doi: 10.1002/ejoc.201800775.
4. Kotha, S.; Ali, R. *Tetrahedron* **2015**, *71*, 1597–1603. doi: 10.1016/j.tet.2015.01.009.
